# Dorsoventral Arrangement of Lateral Hypothalamus Populations in the Mouse Hypothalamus: a Prosomeric Genoarchitectonic Analysis

**DOI:** 10.1007/s12035-022-03043-7

**Published:** 2022-11-11

**Authors:** Carmen Diaz, Margaret Martinez de la Torre, John L. R. Rubenstein, Luis Puelles

**Affiliations:** 1 Department of Medical Sciences, School of Medicine and Institute for Research in Neurological Disabilities, University of Castilla-La Mancha, 02006 Albacete, Spain; 2 Department of Human Anatomy and Psychobiology and IMIB-Arrixaca Institute, University of Murcia, 30100 Murcia, Spain; 3 Nina Ireland Laboratory of Developmental Neurobiology, Department of Psychiatry, UCSF Medical School, San Francisco, California USA

**Keywords:** Peduncular hypothalamus, Patterning, Prosomeres, Genoarchitecture

## Abstract

The lateral hypothalamus (LH) has a heterogeneous cytoarchitectonic organization that has not been elucidated in detail. In this work, we analyzed within the framework of the prosomeric model the differential expression pattern of 59 molecular markers along the ventrodorsal dimension of the medial forebrain bundle in the mouse, considering basal and alar plate subregions of the LH. We found five basal (LH1–LH5) and four alar (LH6–LH9) molecularly distinct sectors of the LH with neuronal cell groups that correlate in topography with previously postulated alar and basal hypothalamic progenitor domains. Most peptidergic populations were restricted to one of these LH sectors though some may have dispersed into a neighboring sector. For instance, histaminergic *Hdc*-positive neurons were mostly contained within the basal LH3, *Nts* (neurotensin)- and *Tac2* (tachykinin 2)-expressing cells lie strictly within LH4, *Hcrt* (hypocretin/orexin)-positive and *Pmch* (pro-melanin-concentrating hormone)-positive neurons appeared within separate LH5 subdivisions, *Pnoc* (prepronociceptin)-expressing cells were mainly restricted to LH6, and *Sst* (somatostatin)-positive cells were identified within the LH7 sector. The alar LH9 sector, a component of the *Foxg1*-positive telencephalo-opto-hypothalamic border region, selectively contained *Satb2*-expressing cells. Published studies of rodent LH subdivisions have not described the observed pattern. Our genoarchitectonic map should aid in systematic approaches to elucidate LH connectivity and function.

**Supplementary Information:**

The online version contains supplementary material available at 10.1007/s12035-022-03043-7.

## Introduction

The *lateral hypothalamus* (LH) is a heterogenous region that is conventionally related to regulation of metabolism, feeding behavior, sleep and wakefulness, inflammatory pain regulation, and motivation [1–11]. It was first recognized as a *feeding center* in the middle of the twentieth century by Hetherington and Ranson, and Anand and Brobeck [12, 13] after stimulation and lesion experiments. Later, the LH was also related to reinforcement learning [14].

Crosby and Woodburne [15] first proposed to divide the mammalian hypothalamus into periventricular, medial, and lateral formations; the limit between the cell-dense medial and the cell-sparse lateral hypothalamus interstitial to the medial forebrain bundle was marked roughly by the course of the fornix tract [16]. Puelles et al. [17] later suggested the possibility to exclude a superficial stratum from the LH that includes the cerebral peduncle (lateral forebrain bundle) and associated subthalamic, entopeduncular and subpial cell populations. These authors also proposed a bi-neuromeric schema of hypothalamic structure (peduncular (PHy) versus terminal (THy) hypothalamus as parts of hp1 and hp2 prosomeres, respectively), and concluded that the LH concept only applies to the peduncular hypothalamus. This notion results from noticing that the intrahypothalamic boundary separating PHy from THy curves around the ventromedial nucleus to reach radially the brain surface rostrally to the peduncle (a similar idea was employed de facto by Nieuwenhuys et al. [18]). Puelles et al. [17] also advanced the notion that the LH probably contains diverse alar and basal neuronal populations along its dorsoventral extent. It is to be noted that conventional columnar analysis of the hypothalamus regards the LH as a longitudinal continuum devoid of alar and basal subdivisions (e.g., [15, 19]), though more recently Swanson [20] conceives the whole hypothalamus as a part of the forebrain basal plate. The respective length axis of the alternative and inconciliable prosomeric and columnar models are at a right angle.

The LH is usually described as containing dispersed cell populations interstitial to the medial forebrain bundle (the latter runs selectively through the intermediate stratum of the peduncular hypothalamus, PHy); LH includes perifornical populations that accompany the fornix as it runs dorsoventrally through the PHy, next to the intrahypothalamic boundary [2, 17, 18, 21–26]. Described LH neuron populations comprise, apart from some dopaminergic neurons, diverse neuropeptidergic populations such as hypocretin and melanin-concentrating hormone producing neurons, implicated in sleeping, waking, feeding, drinking, stress, energy homeostasis, reward, and motivation [9, 27, 28]. Such remarkable functional heterogeneity suggests the possibility that the LH may hold a larger variety of cell types than is presently assumed, so that it might be subdivided into a number of distinct sectors [17].

Nieuwenhuys and collaborators mapped up to fifty descending or ascending fiber components of the rat medial forebrain bundle together with various related neuron populations [18, 23, 24, 29]. These authors proposed the so far most detailed parcellation of the LH, illustrating its cytoarchitectonic heterogeneity in contrast to previous studies that considered it a homogeneous territory [21, 22, 30–35].

Classical columnar studies assumed that the preoptic area (POA) belongs to the rostral hypothalamus, and thus it was held that the LH extends “rostrocaudally” from POA to the ventral tegmental area (VTA) (nowadays the POA is ascribed to the subpallial telencephalon on the basis of molecular evidence; [17, 36, 37]). The VTA was classically wrongly ascribed exclusively to the midbrain but is now interpreted as mesodiencephalic [17, 38]. The diencephalic tegmentum that is presently contemplated in the prosomeric model was associated in classic columnar descriptions to the now outdated concept of “posterior hypothalamus.”

**Fig. 1:**
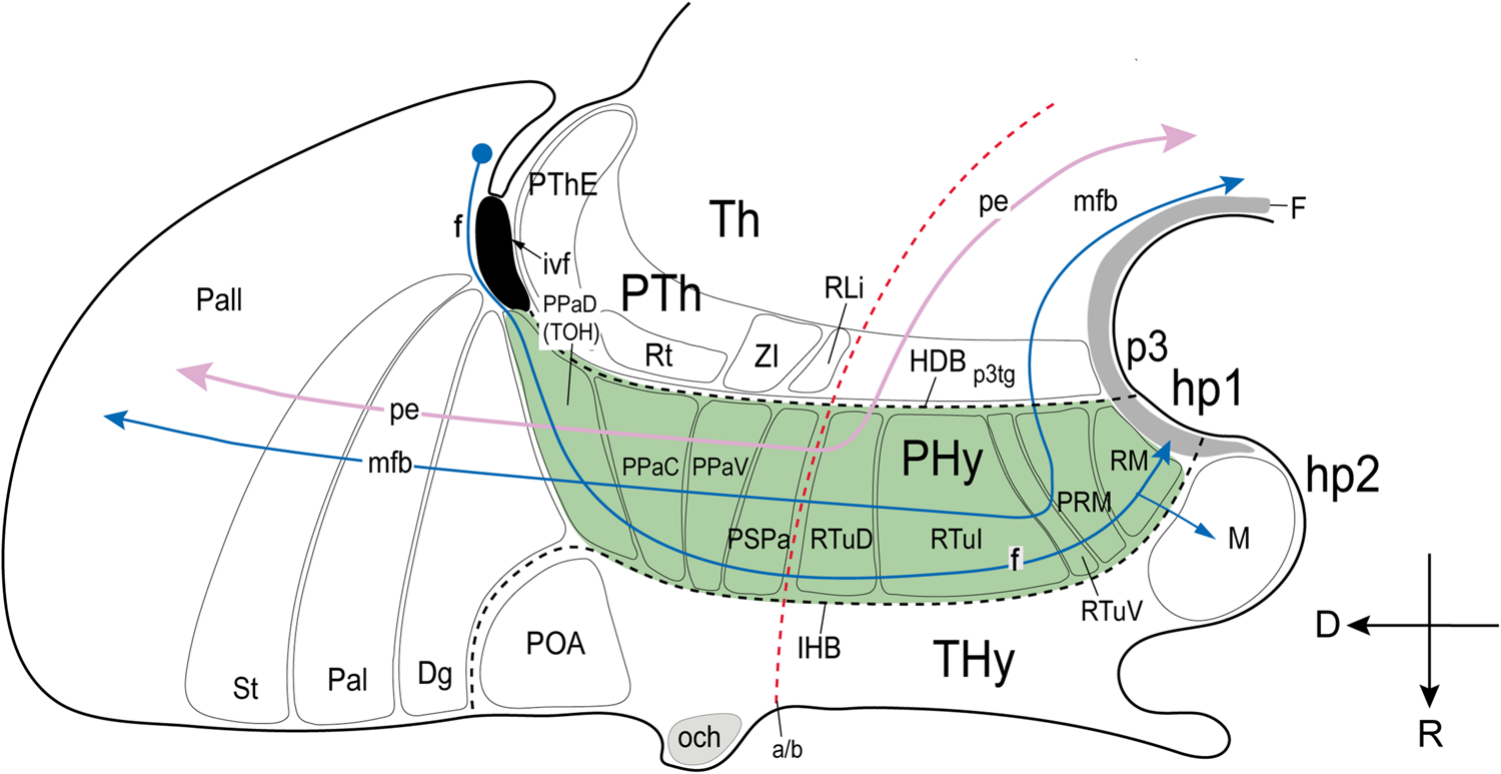
Prosomeric representation of the peduncular hypothalamus (PHy; background in light green) and its relationship with neighboring regions in a schematic sagittal view, following Puelles et al. [17]. The rostral (R) and dorsal (D) spatial directions are indicated. The red dash line indicates the alar/basal limit (a/b). Characteristically, the PHy is traversed dorsoventrally by the medial and lateral forebrain bundles (mfb in blue; lfb or peduncle, pe in pink), as well as by the fornix tract (f in blue). The ascending and descending trajectory of mfb/pe fibers is indicated with arrows. The PHy contacts caudally across the hypothalamic-diencephalic border (HDB) with the diencephalic prosomere p3, representing the prethalamus (PTh). Rostrally, it limits across the intrahypothalamic border (close in front of the fornix tract) with the terminal hypothalamus (IHB; THy). The HDB and IHB boundaries are indicated by black dash lines. The prosomere hp1 is formed jointly by the PHy and the evaginated telencephalon, of which we see subpallial and pallial parts (diagonal area [Dg], pallidum [Pal], striatum [St], pallium [Pall]). The PHy reaches dorsally up to the telencephalic diagonal area domain (Dg). The THy extends into the non-evaginated subpallial preoptic area (POA), representing jointly the prosomere hp2. The PHy is subdivided dorsoventrally in four alar and five basal longitudinal subdomains characterized as molecularly different progenitor areas (Puelles et al. [17]). The alar PHy contains dorsal, central, and ventral peduncular paraventricular (PPa) subregions (PPaD, PPaC, PPaV) as well as an underlying peduncular subparaventricular subdomain (PSPa). The peduncular and terminal parts of PaD have been recently identified as a transitional telencephalo-opto-hypothalamic region (TOH; Morales et al. [37]). The alar PHy subregions contact caudally prethalamic territories. The PPaD (or TOH) borders the prethalamic eminence (PThE), whereas the PPaC contacts the reticular nucleus (Rt), the PPaV limits with the zona incerta (ZI), and the PSPa contacts the rostral liminar area (RLi, [40]). The basal PHy is subdivided dorsoventrally in dorsal, intermediate, and ventral retrotuberal subdomains (RTuD, RTuI, RTuV), complemented more ventrally by the periretromamillary (PRM) and retromamillary (RM) subdomains; RM contacts the floor plate of PHy (F). These basal subdivisions are continuous caudally with the p3 tegmentum (p3tg)

**Fig. 2:**
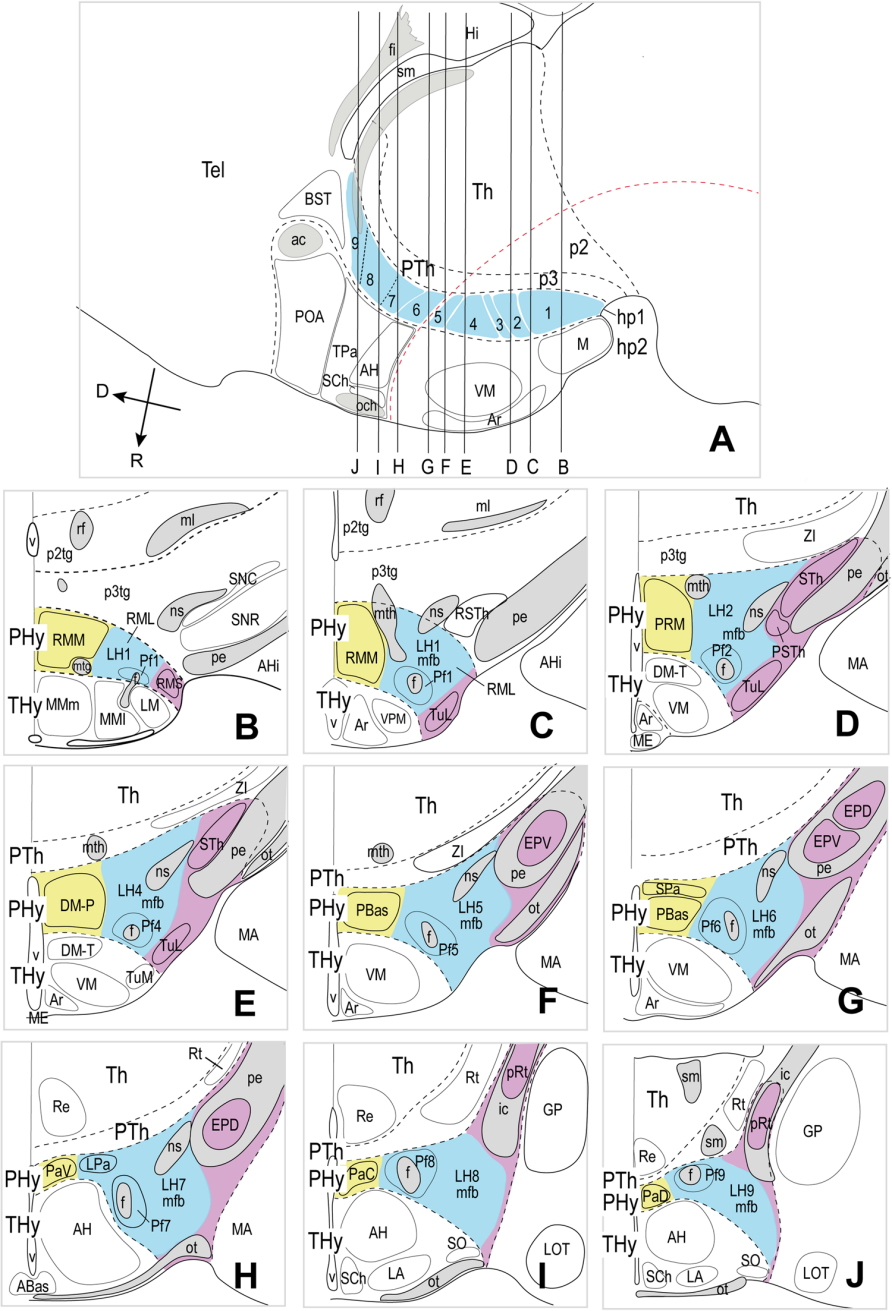
Representative schemata extracted from atlas sagittal and coronal sections (and adapted to the prosomeric model boundaries) showing the location of the lateral hypothalamus in the peduncular hypothalamus. (**A**) Atlas sagittal section through the adult mouse hypothalamus copied and modified from Franklin and Paxinos´s Mouse Brain Atlas [39] (their Fig. 105), in which we entered the dorsoventral positions of the 9 lateral hypothalamic (LH) sectors identified in this work, as well as the coronal section levels shown both in panels **B–J** and in the coronal sections contained in Figs. 3–10. (**B–J**) Conventional coronal schemata in entrodorsal order (representing topologically *horizontal* sections in the prosomeric model; i.e., roughly parallel to the length axis) through the adult hypothalamus, modified from the cited atlas as regards the prosomeric boundaries. The section levels were selected according to characteristic alar or basal anatomic landmarks represented in the atlas (including the tag “mfb” definitory for the LH). Medial (the ventricle) is to the left and rostral at the bottom (the caudal diencephalon lies at the top; compare PTh and Th in **A**). The lateral hypothalamus (LH) forms by conventional definition the radial intermediate stratum (highlighted in blue) found between the superficial (purple) and medial (yellow) hypothalamic strata. Note that while the classical LH in principle reaches the brain surface, Puelles et al. [17] postulated a separate superficial stratum including diverse formations not participating in the LH features: we follow this analysis (see the purple elements). The fornix (f) and nigrostriatal (ns) tracts are identified in gray; other visible tracts except the mfb are also highlighted in gray. The superficial stratum is associated with the peduncle (pe; in gray). Various ventrodorsal LH subdivisions to be documented in the following plates are identified (basal LH1–LH5 and alar LH6–LH9). The main prethalamic and terminal hypothalamic neighboring regions are illustrated as reference landmarks. For additional abbreviations, see “Abbreviations”

As mentioned above, we regard all LH neuronal populations as restricted to the peduncular hypothalamus (PHy), thus named precisely because it is traversed dorsoventrally by the lateral (peduncular) and medial forebrain bundles (pe, mfb; Figs. 1 and 2A; [17, 26]). PHy is bounded caudally by the diencephalic prosomere 3 that includes the characteristic alar *Dlx*-expressing prethalamus and its corresponding basal tegmentum, whereas PHy limits rostrally with the terminal hypothalamus (THy; Fig. 1; [17, 26, 40]). PHy extends dorsally into the subpallio-pallial telencephalic region through a *Foxg1*-positive “telencephalo-opto-hypothalamic” transitional region ([37]; TOH; Fig. 1; see “Discussion”).

Along the radial dimension of the whole alar and basal PHy, LH neurons are included in an outer intermediate stratum lying superficial to the inner intermediate stratum formed by the cell-dense “medial hypothalamic” formations and deep to the superficial stratum containing the peduncle, entopeduncular nuclei, and, at basal levels, the related subthalamic nucleus. This relationship of LH with the covering peduncle occurs particularly caudally, next to the hypothalamo-diencephalic boundary. However, the peduncle does not reach rostralwards the intrahypothalamic boundary (PHy/THy) that bends pialwards around the ventromedial nucleus, so that here, the LH seems to reach the brain surface in relationship with the parasubthalamic nucleus and the nigrostriatal tract (Fig. 2D, E; [17]). The alar part of PHy is divided into paraventricular and subparaventricular progenitor areas (PPa; PSPa; the prefix “P” means “peduncular”). Consequently, the alar LH is divided into paraventricular and subparaventricular subdomains; the PPa domain further displays three dorsoventral subdivisions (dorsal, central, ventral; Figs. 1 and 2A; [17]). The basal PHy is divided dorsoventrally into a retrotuberal domain (subdivided into distinct dorsal, intermediate, and ventral subdivisions), and subjacent periretromamillary and retromamillary progenitor domains (Fig. 1; [17]). The LH thus comprises five differentiable portions at basal levels (Fig. 2A).

The LH had been already subdivided tentatively by Puelles et al. [17] in three alar domains (named—according to the nearest prethalamic neighbor area—pre-eminential, pre-reticular, and pre-incertal) and two basal domains (dorsobasal and ventrobasal). The dorsalmost (pre-eminential) LH subregion was characterized by glutamatergic *Fezf2*- and *Tbr1*-positive cells; the underlying pre-reticular LH subregion showed glutamatergic *Tbr1*-expressing cells, and the ventral pre-incertal LH subregion contained GABAergic *Dlx5/6*- and *Nkx2.2/Lhx6*-labeled cells. In the basal plate, the dorsobasal LH subdomain was characterized by the presence of glutamatergic *hypocretin* (*Hcrt*)*-*expressing neurons, whereas the underlying ventrobasal LH subdomain displayed GABAergic and peptidergic *Cart-* and *Gal-*expressing cells. However, such potential alar and basal subregions related to the hypothalamic course of the medial forebrain bundle were not extensively mapped genoarchitectonically [17, 41].

In a recent single-cell transcriptomic study of the mouse LH, fifteen glutamatergic and fifteen GABAergic cell clusters were identified [42]. This analysis apparently focused only on the retrotuberal cell populations of the basal hypothalamus, which was the only LH subregion illustrated in three coronal section levels through the dorsomedial hypothalamic nucleus ([42]; their Fig. 1b). If this interpretation is accurate, these authors did not consider in their analysis various other alar and basal LH cell populations intermingled with the hypothalamic course of the medial forebrain bundle, as predicted by prosomeric analysis ([17, 26]; present data). The same occurs with another transcriptomic analysis by Wang et al. (2021). Other recent hypothalamus transcriptomic studies have referred only superficially to the LH, or not at all [ 43–48].

Our aim in the present work was to analyze systematically the diverse LH regions associated with different dorsoventral levels of the medial–lateral forebrain bundle along the alar and basal parts of PHy, describing characteristic aspects of their molecular profile and checking their number and topography. Each distinct genoarchitectonic level can be tentatively explained as representing cells derived from a distinct progenitor domain. This possibility implied examining the apparent topographic relationships of molecularly distinct adult LH populations with the alar and basal progenitor domains reported within PHy [17, 41]. Of course, it cannot be assumed that all neuron types remain within the radial domain where they were born, so that partial dispersion or even massive migrations into other domains are possible. Nevertheless, we hypothesized that each distinct dorsoventral PHy domain may originate one or more specific LH cell subpopulations having a particular molecular profile. These would appear in the mantle either intermixed in a salt and pepper manner or stratified in a temporal sequence pattern. To that end, we examined empirically identified selective lateral hypothalamic cell markers in images downloaded from in situ hybridization data of the Allen Brain platform (© 2008 Allen Mouse Brain Atlas; Allen Developing Mouse Brain Atlas). According to our analysis, nine molecularly distinct DV tiers of the LH can be distinguished within PHy, four of them in the alar part and five within the basal part.

## Materials and Methods

Most of our analysis was based on in situ hybridization (ISH) images downloaded from the Allen Developing Mouse Brain Atlas (https://developingmouse.brain-map.org) and Allen Mouse Brain Atlas (https://mouse.brain-map.org/). Our analysis was focused on late embryonic stages (E18.5) and postnatal stages from P4 to P56 in sagittal and coronal sections when available. According to documentation given by the Allen platform, male specimens of the C57BL/6 J mouse strain were used for the E15.5 through adult stages.

To select markers, we applied the AGEA informatic tool in the Allen Mouse Brain Atlas which is based on spatial correlations of gene expression data. We had no preconceived notion about how many molecularly different parts of the lateral hypothalamus existed. We just sampled unbiasedly genes detected by AGEA in 2–3 dorsoventral points at each of the areas identified in the Allen source as either “basal peduncular hypothalamus” or “alar peduncular hypothalamus.” We only assumed that the LH is restricted to the peduncular hypothalamus and that it extends through both the alar and basal regions (see concept in Puelles et al. 2012). The AGEA tool is not resolutive enough to place the search seed in a more precise way. Each search point provided a list of some 200 candidate genes. We screened each of these markers, selecting empirically those showing in sagittal sections clearcut differential expression along ventrodorsal levels of the lateral hypothalamus (LH). Two of the authors (CD, LP) analyzed and discussed the expression pattern of each gene to arrive at a consensus decision about the interest of the pattern for the present purpose. Genes with low ISH signal, widespread signal, or high background were discarded. In a later stage of the study, a few other genes of interest reported in published transcriptomic studies and present in the Allen database were added to our list (we checked all genes mentioned in transcriptomic studies).

**Fig. 3:**
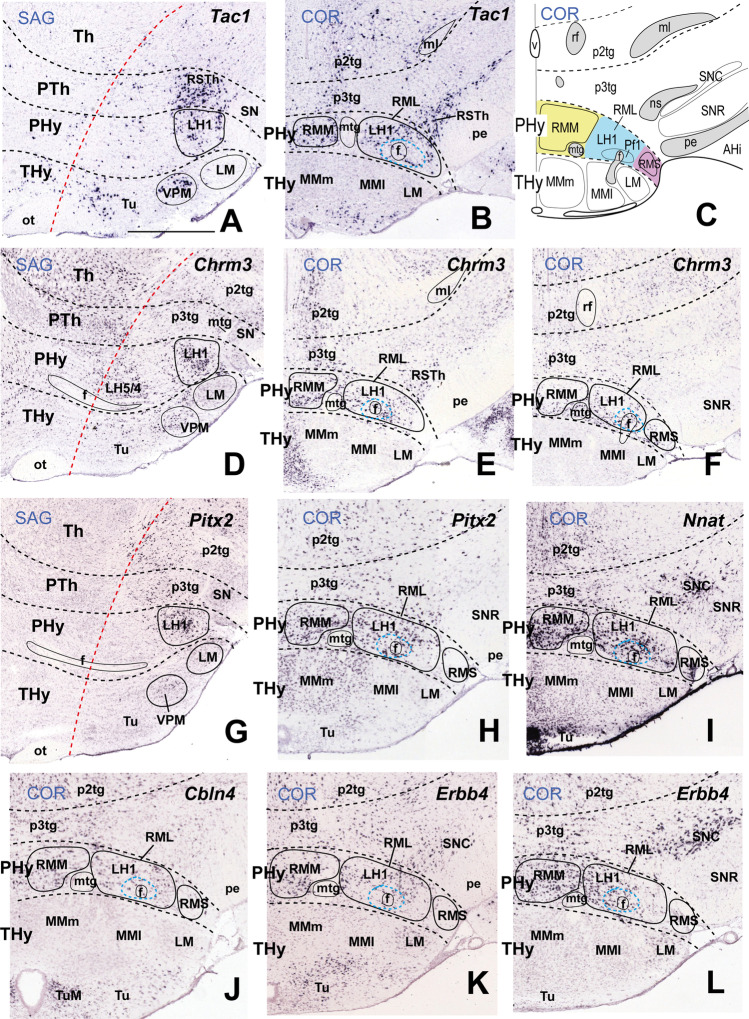
In situ hybridization expression mapping at P56 of various markers in sector 1 of the basal lateral hypothalamus (LH1; also known as “lateral retromamillary nucleus,” RML). Chosen sagittal and coronal section images (identified according to characteristic landmarks consistent with Fig. 2 atlas data) were downloaded from the Allen Developing Mouse Brain Atlas. The sagittal sections indicate the restricted dorsoventral topography of the labeled LH cells, whereas the coronal sections demonstrate the corresponding either diffuse or restricted topography within the illustrated LH level as defined in Fig. 2. Some markers are shown only in coronal sections (with corresponding level landmarks). Sagittal (**A**, **D**, **G**) and horizontal (conventional coronal) (**B**, **E**, **F**, **H–L**) sections are shown (marked as SAG or COR at the upper left corner), while (**C**) illustrates a schematic coronal section through the hypothalamus taken across LH1 (in blue), extracted from Fig. 2; corresponding medial and superficial retromamillary strata relating to LH1 (yellow and purple, respectively) are also shown. In sagittal sections, dorsal direction points to the left, and rostral direction to the bottom; in horizontal/coronal sections, medial direction points to the left, and rostral direction to the bottom. LH1 is characterized by cells expressing *Tac1* (**A–C**), *Chrm3* (**D–F**), *Pitx2* (**G**, **H**), and *Nnat* (**I**). The *Cbln4* (**J**) and *Erbb4* (**K**, **L**) markers also appear expressed in LH1 cells, though they are detected likewise in cells belonging to the overlying LH2 sector (Table 2). LH1 is part of the retromamillary *basal hypothalamic* domain (Fig. 1). Black dash lines indicate the thalamo-prethalamic, hypothalamo-prethalamic, and intrahypothalamic *transversal* interprosomeric boundaries. A red dash line denotes the alar-basal limit. The LH1 area is surrounded with a black line, similarly as neighboring structures of the same radial domain (medial and superficial retromamillary nuclei, RMM, RMS). The mamillo-tegmental tract (mtg) separates the LH1 and RMM areas. The fornix (f) courses dorsoventrally (transected) next to the intrahypothalamic boundary; the LH subpopulation identified as “perifornical 1” (Pf1) is surrounded by a blue dash line (due to the dorsoventral course of f, perifornical populations exist at all LH subdivisions, thus needing an alphanumeric identification). Scale bar for all images, indicated in (**A**): 730 µm. For additional abbreviations, see “Abbreviations”

**Fig. 4:**
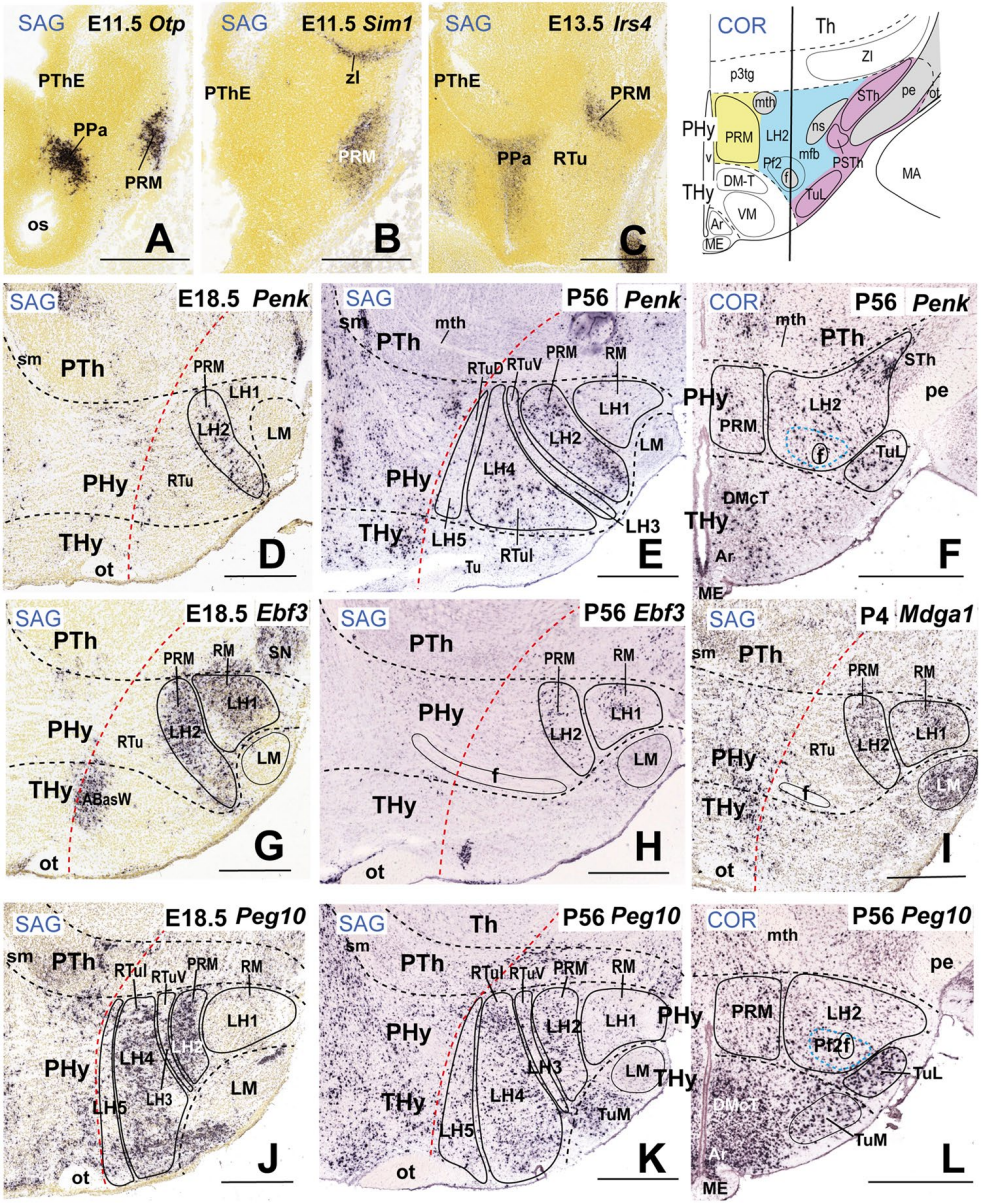
Molecular characterization of sector 2 of the lateral hypothalamus (LH2). LH2 is part of the periretromamillary basal hypothalamic peduncular subdomain (PRM). All sagittal or coronal (topologically horizontal) section images (marked as SAG or COR at the upper left corner) were downloaded from the Allen Developing Mouse Brain Atlas. The sagittal sections indicate a restricted dorsoventral topography of the labeled LH2 cells, whereas the coronal sections demonstrate the corresponding either diffuse or restricted topography within the illustrated LH2 level as defined in Fig. 2. Some markers are shown only in coronal sections (with corresponding level landmarks). (**A–C**) Identification of the PRM/LH2 domain by selective expression of *Otp*, *Sim1*, and *Irs4* in three sagittal sections at the indicated embryonic stages. A schematic horizontal/coronal section through the LH2 level (extracted from Fig. 2) is illustrated in the upper right inset. The LH2 area is in blue and the medial and superficial PRM strata in yellow and purple, respectively; the latter stratum contains the subthalamic and lateral tuberal nuclei (STh, TuL) besides the peduncle. The vertical black line in the inset indicates roughly the sagittal section levels shown in (**D**), (**E**), (**G**), (**H**), (**J**), and (**K**). The black dash lines in **D–L** indicate the thalamo-prethalamic, hypothalamo-prethalamic, and intrahypothalamic transverse interprosomeric boundaries, whereas the red dash line indicates the alar-basal limit. The black lines delimit LH2 and other basal LH subregions. Cells surrounding the fornix tract (f) within LH2 are classified as the perifornical subpopulation 2 (Pf2; blue dash line). There is restricted expression of *Penk* signal in LH2 cells at E18.5 (**D**; this cell population was slightly more distinct at this stage than at P56; **E**, **F**). Note adult *Penk* signal appears in basal LH2 as well as in LH4 cells, and in scattered alar LH cells (**E**). *Ebf3* expression appears in LH2 cells and subjacent LH1 cells both at E18.5 (**G**) and at P56 (**H**; less intense signal). *Mdga1* expression is present in the LH2 and LH1 sectors (**I**). *Peg10* signal appears largely restricted to basal LH2 and LH4 cells at both E18.5 and P56 (**J**, **K**, **L**). Scale bars: (**A–D**, **G**, **I**, **J**) = 500 µm; (**E**, **F**, **H**, **K**, **L**) = 700 µm. For additional abbreviations, see the “Abbreviations”

**Fig. 5:**
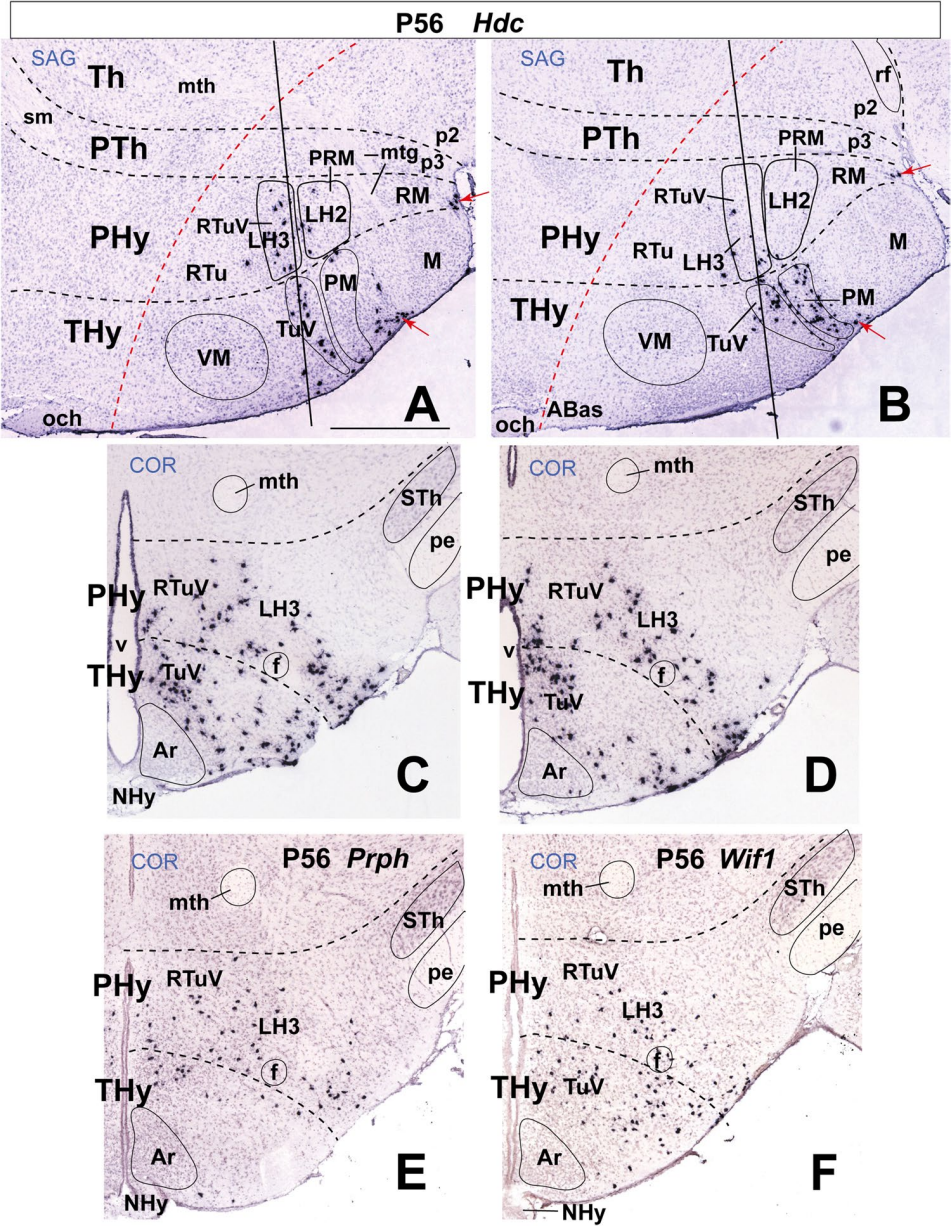
Large-sized *Hdc*-positive histaminergic cells develop in the ventral retrotuberal (RTuV) domain of the basal peduncular hypothalamus at P56 (this feature also extends into terminal hypothalamus; TuV in **A**, **B**). The RTuV contains as its intermediate stratum sector 3 of the lateral hypothalamus (LH3) which lies dorsal to the LH2 or periretromamillary domain (PRM). Sagittal and coronal (topologically horizontal) section images (marked as SAG or COR at the upper left corner) were downloaded from the Allen Developing Mouse Brain Atlas. The sagittal sections indicate a restricted dorsoventral topography of the labeled LH3 cells, whereas the coronal sections demonstrate the corresponding either diffuse or restricted topography within the illustrated LH3. The black dash lines indicate the thalamo-prethalamic, hypothalamo-prethalamic, and intrahypothalamic transverse prosomeric boundaries. The red dash line indicates the alar-basal limit. (**A**, **B**) Two sagittal sections (**A** lateral to **B**) showing *Hdc* expression in the RTuV (including positive LH3 cells). Note *Hdc-*positive cells appear in RTuV and TuV jointly to other positive cells in the premamillary area (PM) and scarce positive cells in subpial position (red arrows). The black lines indicate the coronal levels shown in (**C**) and (**D**). There is significant radial dispersion of *Hdc* cells from periventricular to superficial strata in both RTuV and TuV domains. (**E**) Coronal section through LH3 showing *Prph*-expressing cells mainly restricted to the RTuV area. (**F**) Coronal section through LH3 showing *Wif1*-positive cells. Note the distribution of *Wif1* cells is analogous to that of *Hdc* cells (compare **C**, **D** to **F**). *Prph* and *Wif1* are known markers expressed in *Hdc* cells (see text). Scale bars = 700 µm. For additional abbreviations, see “Abbreviations”

**Fig. 6:**
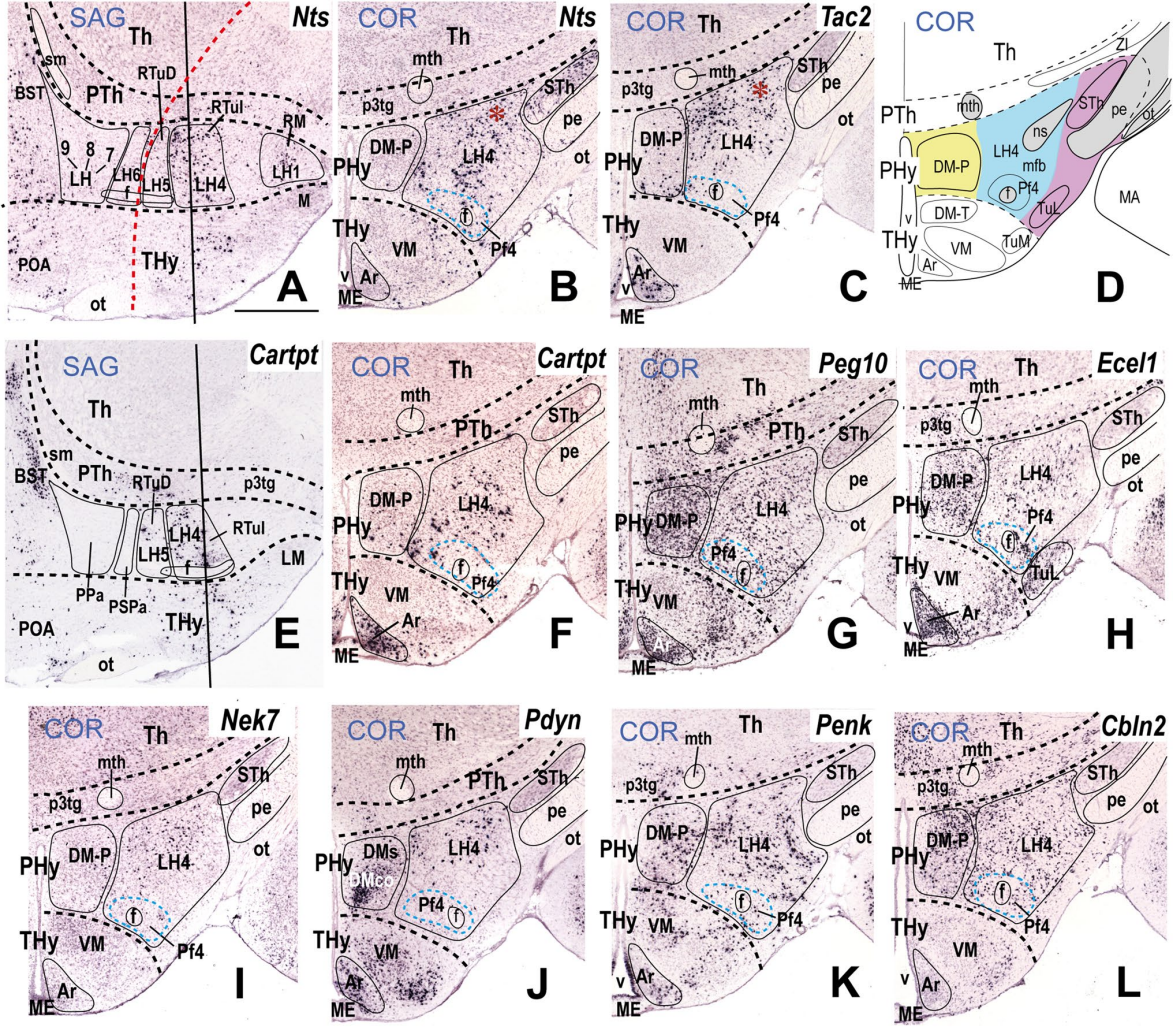
Expression of selected markers in sector 4 of the lateral hypothalamus (LH4) in sagittal and coronal (topologically horizontal) sections (marked as SAG or COR at the upper left corner) downloaded from the Allen Developing Mouse Brain Atlas at P56. LH4 is the intermediate stratum of the intermediate retrotuberal domain (RTuI) as shown in the section schema (**D**; LH4 in blue; schema extracted from Fig. 2). The sagittal sections reveal restricted dorsoventral topography of some labeled LH4 cells, whereas the coronal sections demonstrate the corresponding either diffuse or restricted topography within the illustrated LH4 level as defined in **D**. Some markers are shown only in coronal sections (with corresponding level landmarks). The black dash lines indicate the thalamo-prethalamic, hypothalamo-prethalamic, and intrahypothalamic transverse prosomeric boundaries. The red dash line indicates the alar-basal limit. The vertical black lines in the sagittal sections (**A**), (**E**), and (**K**) indicate coronal section levels in (**B**), (**F**), and (**L**), respectively. Cells around the fornix tract (f) comprise the perifornical subpopulation 4 (Pf4; blue dash line) inside the LH4 sector. (**A**, **B**) Sagittal and coronal sections, respectively, showing *Nts* (neurotensin) signal in LH4 neurons. Note *Nts*-positive cells are mainly restricted to LH4, though the migrated subthalamic nucleus (STh) seen in **B** also contains such cells. (**C**) A coronal section through LH4 showing localized *Tac2* (tachykinin 2) neurons. Red asterisks in (**B**) and (**C**) indicate *Tac2-* and *Nts-*expressing cells in a similar distribution. (**D**) Schematic coronal section identifying LH4 (in blue) relative to local medial (e.g., the peduncular dorsomedial nucleus DM-P) and superficial (e.g. subthalamic and lateral tuberal nuclei) strata are in yellow and purple, respectively. Sagittal and coronal sections (**E**, **F**) show peptidergic *Cartpt*-positive cells restricted mainly to LH4. LH4 cells show restricted expression of *Peg10* (**G**), *Ecel1* (**H**), *Nek7* (**I**), and *Pdyn* (**J**). Peptidergic *Penk*-expressing cells are also localized in LH4 (**K**). (**L**) *Cbln2*-expressing cells are present in the intermediate LH4 stratum as well as in the local medial stratum, the dorsomedial hypothalamic nucleus (DM-P). Scale bar for all images, indicated in (**A**): 700 µm. For additional abbreviations, see “Abbreviations”

**Fig. 7:**
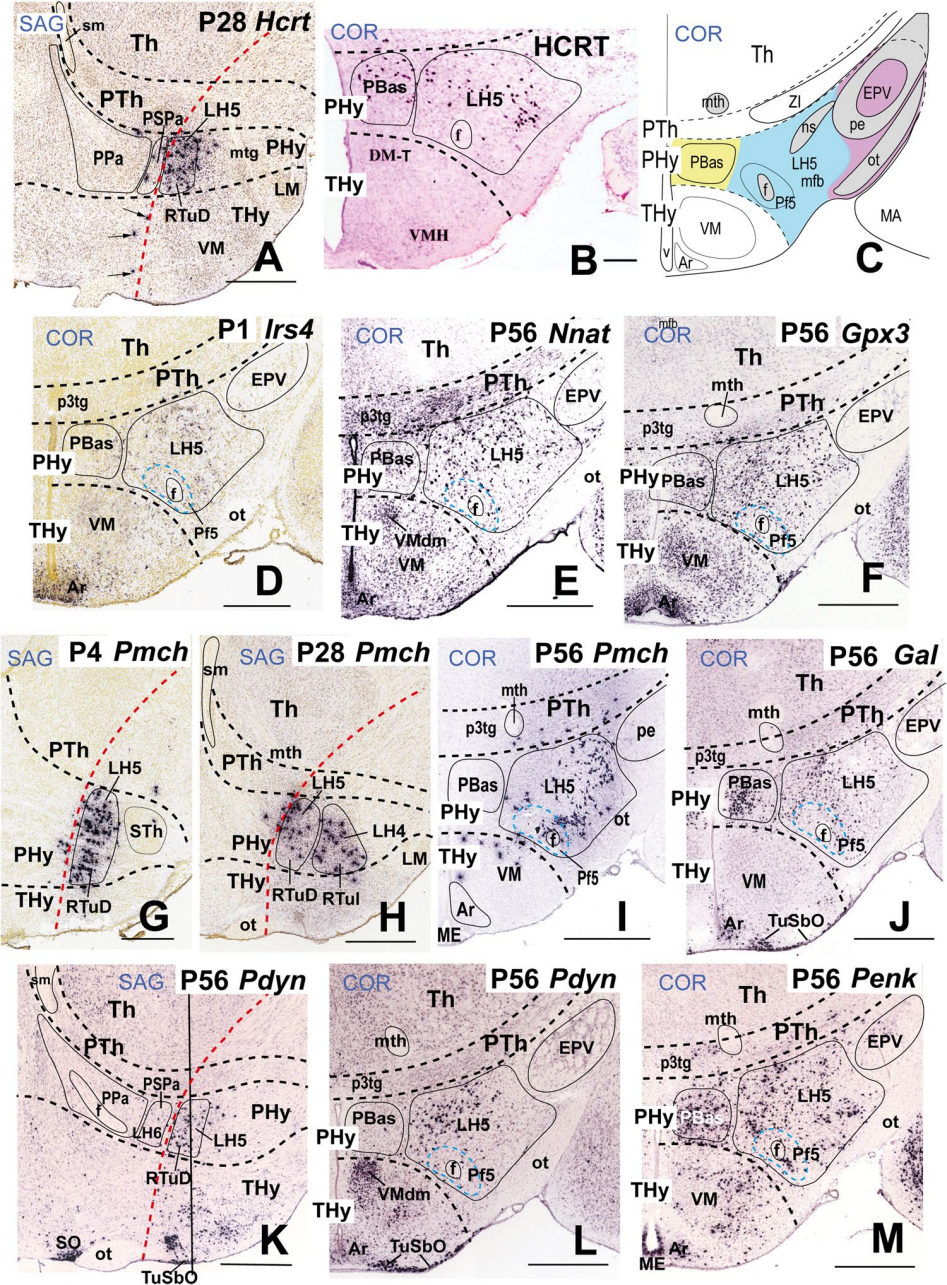
Expression of selected markers in the basal sector 5 of the lateral hypothalamus (LH5) in sagittal (**A**, **G**, **H**, **K**) and coronal (topologically horizontal; **D**–**F**, **I**, **J**, **L**, **M**) sections (marked as SAG or COR at the upper left corner) at indicated stages. The LH5 sector is the intermediate stratum of the dorsal retrotuberal domain (RTuD) of the basal peduncular hypothalamus. All these sections were downloaded from the Allen Developing Mouse Brain Atlas. The sagittal sections indicate a restricted dorsoventral topography of the labeled LH5 cells, whereas the coronal sections demonstrate the corresponding either diffuse or restricted topography within the illustrated LH5 level as defined in **C**. Some markers are shown only in coronal sections (with corresponding level landmarks). The black dash lines indicate the thalamo-prethalamic, hypothalamo-prethalamic, and intrahypothalamic transverse prosomeric boundaries. The red dash line indicates the alar-basal limit. A black vertical line indicates the approximate horizontal (conventional coronal) level in (**L**). Cells surrounding the fornix tract (f) comprise the perifornical subpopulation 5 (Pf5; blue dash line) within the LH5 sector. (**A**) Sagittal section showing hypocretin *Hrct*-expressing LH5 cells at P28; black arrows indicate some apparently displaced *Hcrt*-positive cells in the overlying alar hypothalamus. (**B**) Adult HCRT-immunoreactive cells in a coronal section through LH5. This image is modified from Peyron et al. ([75]; their Fig. 3C; Copyright [1998] Society for Neuroscience). (**C**) Schema representing LH5 (in blue) as an intermediate stratum intercalated between the medial (in yellow) and superficial (in purple) RTuD strata. Various characteristic anatomic landmarks are seen at this level, such as the hypothalamic peduncular ventral entopeduncular and posterobasal nuclei (EPV, PBas), the terminal ventromedial and arcuate nuclei (VM, Ar), and the prethalamic incertal complex (ZI). Cells expressing *Irs4* (**D**), *Nnat* (**E**), and *Gpx3* (**F**) appear within LH5. (**G**, **H**) Sagittal sections at P4 and P28 stages showing *Pmch* positive cells within LH5; the comparison suggests a progressive dispersion. (**I**) Coronal distribution of adult LH5 peptidergic *Pmch*-expressing cells. (**J**) Adult *Gal* cell subpopulation of LH5 in coronal section. Adult *Pdyn*-expressing cells in sagittal and coronal sections (**K**, **L)**. (**M**) *Penk* signal in a coronal section through LH5. Scale bars: (**A**, **E**, **F**, **H–M**) = 700 µm; (**B**) = 275 µm; (**D**, **G**) = 500 µm. For additional abbreviations, see “Abbreviations”

**Fig. 8:**
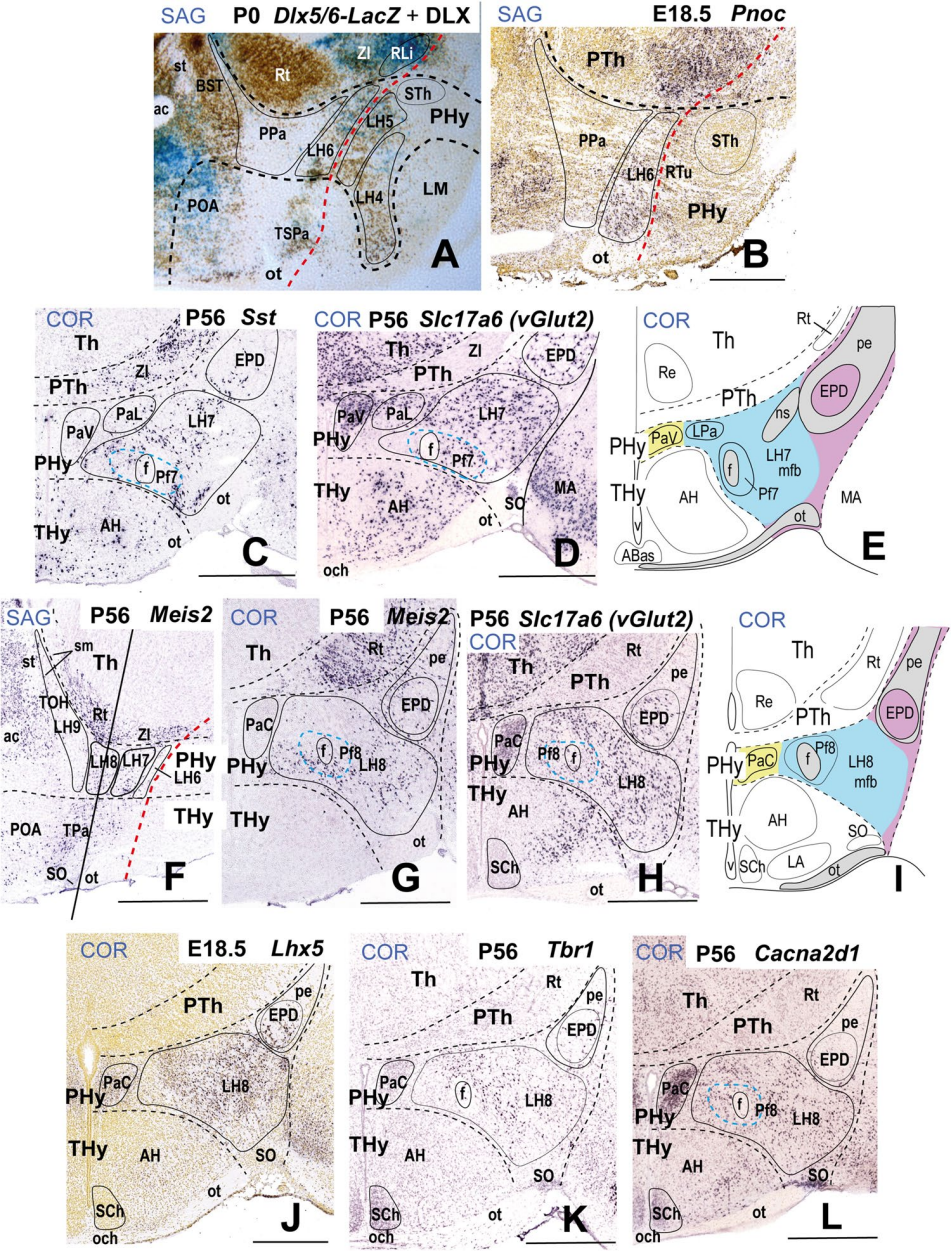
This figure shows expression of selected markers in the LH6–8 sectors of the *alar peduncular hypothalamus*. The black dash lines indicate the thalamo-prethalamic, hypothalamo-prethalamic, and intrahypothalamic transverse prosomeric boundaries. The red dash line indicates the *longitudinal* alar-basal limit. Sagittal and coronal (topologically horizontal) sections are marked as SAG or COR at the upper left corner). The sagittal sections indicate a restricted dorsoventral topography of the labeled LH cells at indicated levels, whereas the coronal sections demonstrate the corresponding either diffuse or restricted topography within the illustrated LH levels as defined in Fig. 2 and panels 8 **E**, **I**. Some markers are shown only in coronal sections (with corresponding level landmarks). (**B–D**, **F–H**, **J–L**) Images of sections downloaded from the Allen Developing Mouse Brain Atlas. (**A**) A sagittal section through the P0 mouse hypothalamus showing β-galactosidase activity (blue) controlled by a *Dlx5/6-LacZ* transgenic construct and a pan-Distalless (DLX) immunoreaction (polyclonal antibody) that recognizes all forms of DLX (brown; modified from Puelles et al. [17]; their Fig. 8.15E). The alar sector 6 of the lateral hypothalamus (LH6) corresponds to the intermediate stratum of the peduncular subparaventricular area (PSPa) and contains a mixture of blue and brown *Dlx*-expressing cells. LH6 contacts caudally the equally strongly *LacZ*-labeled prethalamic rostral liminar band (RLi). (**B**) Sagittal section showing LH6 *Pnoc*-expressing cells at E18.5; note *Pnoc* cells appear intermingled with unlabeled medial forebrain tract fibers, particularly where the peduncular hypothalamus (PHy) bends laterally around the terminal hypothalamus (see Fig. 2G). (**C**) *Sst* expression in sector 7 of the lateral hypothalamus (LH7) in a coronal section at P56. The LH7 sector belongs to the ventral subdivision of the peduncular paraventricular area (PPaV) and contacts caudally the prethalamic incertal complex (ZI). (**D**) The whole peduncular paraventricular radial region contains glutamatergic *Slc17a6* (*vGlut2*)-positive cells. The ventral and lateral paraventricular nuclei (PaV, PaL), as well as the dorsal entopeduncular nucleus (EPD) appear as landmarks at the LH7 level; the neighboring prethalamus (PTh) contains mainly *Slc17a6* (*vGlut2*)-negative GABAergic cells. (**E**) Schema of a coronal section through LH7 (in blue) at the level of the ventral paraventricular subarea, comparable to the section illustrated in (**D**). Note the medial stratum comprises characteristically the ventral paraventricular nucleus (PaV; in yellow) whereas the superficial stratum contains the dorsal entopeduncular nucleus (EPD; in purple). (**F-L**) These figures illustrate expression of several markers in the sector 8 of the lateral hypothalamus (LH8). LH8 forms the intermediate stratum of the central subdivision of the peduncular paraventricular area (PPaC) and contacts caudally the prethalamic reticular complex (Rt), as illustrated in (**I**). LH8 *Meis2*-positive cells are shown in sagittal and coronal sections at P56 (**F**, **G**). (**H**) A coronal section through the peduncular paraventricular area showing widespread *Slc17a6* (*vGlut2*)-positive glutamatergic cells; relatively abundant *Slc17a6* (*vGlut2*)-positive cells are also observed in the thalamus (Th), contrasting with their absence in the PTh. (**I**) Schema of a coronal section through the LH8 sector with the same color-code as in (**E**). (**J**) Abundant *Lhx5*-expressing cells are present in the LH8 sector at E18.5; note the dorsal entopeduncular nucleus (EPD) and central paraventricular nucleus (PaC) contain *Lhx5-*positive populations. (**K**) Disperse *Tbr1*-positive cells also appear in LH8, as well as in EPD and PaC populations at P56. (**L**) A coronal section shows LH8 *Cacna2d1*-positive cells at P56. Scale bars: (**B**, **I**) = 500 µm; (**C–H**, **K**) = 900 µm. For additional abbreviations, see “Abbreviations”

**Fig. 9:**
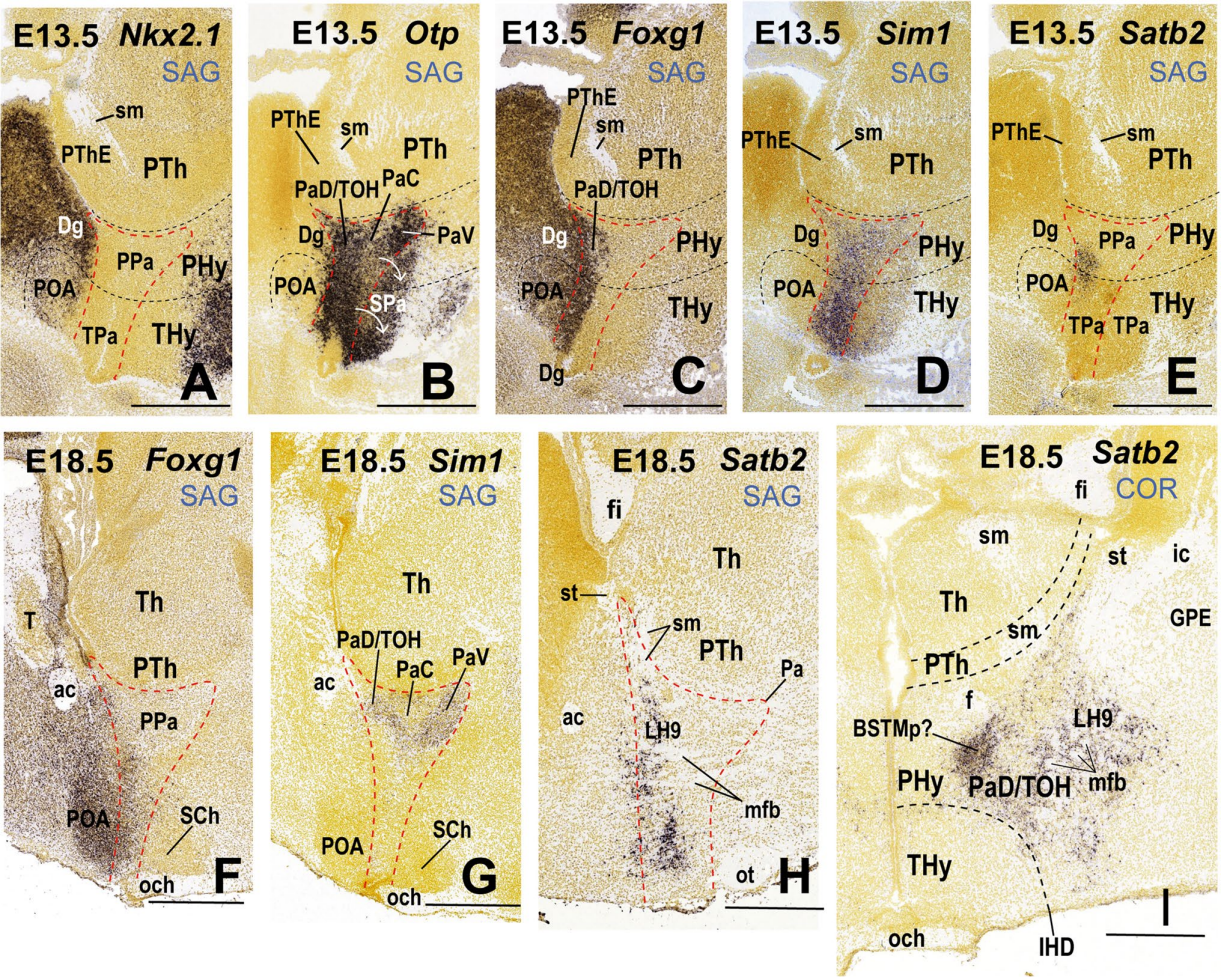
Differential expression of characteristic subpallial (*Nkx2.1*, *Foxg1*) and alar hypothalamic (*Sim1*, *Otp*) markers, compared to LH9 *Satb2* expression in the dorsal paraventricular hypothalamic subdomain (PaD), which apparently coincides with the transitional “telencephalo-opto-hypothalamic area” (TOH) of Morales et al. [37]. These images are presented to clarify this still little-understood forebrain region. Sagittal and coronal sections are marked as SAG or COR at the upper right corner. A red dashed line outlines the paraventricular domain (Pa). The black dash lines indicate the thalamo-prethalamic, hypothalamo-prethalamic, and intrahypothalamic transverse prosomeric boundaries. (**A–E**) Comparison of the expression of *Nkx2.1*, *Otp*, *Foxg1*, *Sim-*, and *Satb2* in sagittal sections at approximately the same level at E13.5 (less morphogenetic deformation of the relevant boundaries). Note PaD/TOH is a narrow area at the hypothalamo-telencephalic border where *Otp*/*Sim1* signals (**B**, **D**) overlap *Foxg1* expression (**C**), in the absence of *Nkx2.1* expression (**A**). White arrows indicate apparent *Otp* cells migrated to the subjacent subparaventricular domain (**B**; SPa). A *Satb2*-positive subpopulation correlates topographically with the PaD/TOH area (**E**). (**F**, **G**) Two mutually equivalent sagittal sections showing *Foxg1* and *Sim1*expression in this area at E18.5. (**H**) A sagittal section lateral to sections (**F**) and (**G**) shows *Satb2*-expressing cells intermingled with unlabeled medial forebrain tract fibers (mfb) within LH9. (**I**) LH9 *Satb2*-positive cells in a coronal section at E18.5. Note the LH9 sector is the intermediate stratum of the PaD/TOH subdomain found at the peduncular alar hypothalamus. Medial periventricular *Satb2*-positive cells may correspond to the posterior subdivision of the bed nucleus of the stria medullaris (BSTMp; Morales et al. [37]). Scale bars: (**B–E**) = 500 µm; (**F–I**) = 600 µm. For additional abbreviations, see “Abbreviations”

We identified 59 genes, classified according to protein type and related functions (Table 1). They were differentially expressed along various ventrodorsal LH levels (Table 2). The list of the selected genes illustrated in Figs. 2, 3, 4, 5, 6, 7, 8 and 9, together with the references of their respective experiments and images downloaded from the Allen database, are indicated in Supplementary Table 1.

**Table 1:** Classification of analyzed gene markers according to type of protein, and their associated functions

Gene	Protein	Type of protein	Function	References/links
*Calb2*	Calretinin (CR)	Calcium binding protein	Modulation of neuronal excitability (calcium signaling)	https://www.ncbi.nlm.nih.gov/gene/794/ortholog/?scope=7776
*S100a10 (calpatin)*	S100 calcium-binding protein A10 (calpactin)	Calcium binding protein	Intracellular proteases activated by calcium. Modulator of cellular processes such as cell cycle progression and differentiation	https://www.ncbi.nlm.nih.gov/gene/6281/ortholog/?scope=32523
*Cacna2d1*	Calcium channel, voltage-dependent, alpha2/delta subunit 1	Calcium chanel	It regulates calcium current density and activation/inactivation kinetics of the calcium channel	https://www.uniprot.org/uniprot/O08532
*Cbln2*	Cerebellin 2 precursor protein	Cerebellin family	Secreted neuronal glycoprotein. Formation and/or function of synapses	[1]; https://www.uniprot.org/uniprot/Q8IUK8
*Cbln4*	Cerebellin 4 precursor protein	Cerebellin family	Secreted neuronal glycoprotein; formation and/or function of synapses	[1]; https://www.uniprot.org/uniprot/Q8IUK8
*Parm1 (9130213B05RIK)*	Prostate androgen-regulated mucin-like protein 1	Endoplasmic reticulum molecule	Cell differentiation Involved potentially in positive regulation of telomerase activity. Unknown function in the brain	https://www.ncbi.nlm.nih.gov/gene/231440
*Ecel1*	Endothelin converting enzyme-like 1	Enzyme (endopeptidase)	Associated with nerve development and regeneration	[2, 49]; https://www.ncbi.nlm.nih.gov/gene/9427/ortholog/?scope=89593
*Nek7*	NIMA (never in mitosis gene a)-related kinase	Enzyme (serine/threonine protein kinase)	Role in mitotic cell cycle progression. Microtubule regulator	https://www.uniprot.org/uniprot/Q9ES74
*Gad1 (Gad67)*	Glutamate decarboxylase 1	Enzyme; GABAergic neuron marker	Synthesis of GABA	[3, 50]; https://www.ncbi.nlm.nih.gov/gene/2571/ortholog/?scope=89593
*Gad2 (Gad65)*	Glutamate decarboxylase 2	Enzyme; GABAergic neuron marker	Systhesis of GABA	[3, 50]; https://www.ncbi.nlm.nih.gov/gene/2572/ortholog/?scope=7776
*Gpx3*	Glutathione peroxidase 3	Enzyme	Detoxification of hydrogen peroxide	https://www.ncbi.nlm.nih.gov/gene/2878/ortholog/?scope=7776
*Hdc*	Histidine decarboxylase	Enzyme; histaminergic cell marker	Synthesis of histamine; neurotransmission	https://www.ncbi.nlm.nih.gov/gene/3067/ortholog/?scope=7776
*Nos1*	Nitric oxide synthase 1	Enzyme	It catalyzes the production of nitric oxide (NO) from l-arginine	https://www.ncbi.nlm.nih.gov/gene/4842/ortholog/?scope=7776
*Mdga1*	MAM domain containing glycosylphosphatidylinositol anchor 1 protein	Immunoglobulin family	Cell adhesion; formation or maintenance of inhibitory synapses; radial migration of cortical neurons	[4]: https://www.ncbi.nlm.nih.gov/gene/266727/ortholog/?scope=117570
*Wif1*	WNT inhibitory factor 1	Lipid-binding protein	Implicated in WNT signaling during embryonic development	https://www.ncbi.nlm.nih.gov/gene/11197/ortholog/?scope=89593
*Slc17a6 (vGlut2)*	Solute carrier family 17 (sodium-dependent inorganic phosphate cotransporter), member 6	Membrane transport protein (vGLUT2); glutamatergic cell marker	Vesicular glutamate transporter. Involved in glutamatergic neurotransmission	[5, 51]; https://www.omim.org/entry/607563?search=Slc17a6&highlight=slc17a6
*Slc32a1*	Solute carrier family 32 (GABA vesicular transporter), member 1	Membrane transport protein; GABAergic cell marker	Vesicular inhibitory amino acid transporter, GABA and glycine transporter	[6]; https://www.ncbi.nlm.nih.gov/gene/11197/ortholog/?scope=89593
*Cartpt*	CART (cocaine- and amphetamine-regulated transcript) prepropeptide	Neuropeptide	Role in feeding, drug reward, stress, cardiovascular function, and bone remodeling	https://www.ncbi.nlm.nih.gov/gene/9607/ortholog/?scope=89593; https://www.omim.org/entry/602606?search=Cartpt&highlight=cartpt
*Crh*	Corticotropin-releasing hormone	Neuropeptide	Central driver of the stress hormone system	https://www.ncbi.nlm.nih.gov/gene/1392/ortholog/?scope=7776
*Gal*	Galanin	Neuropeptide	Role in nociception, feeding and energy homeostasis, osmotic regulation, and water balance	https://www.ncbi.nlm.nih.gov/gene/51083/ortholog/?scope=89593
*Hcrt*	Hypocretin (orexin)	Neuropeptide	Role in arousal, feeding, and energy metabolism	[7]
*Igf1*	Insulin-like growth factor 1	Neuropeptide	Neuroplasticity	[8, 9]
*Nts*	Neurotensin	Neuropeptide	Role in arousal and regulation of hyperthermia. Involved in regulation of dopamine pathways	[10, 52]
*Pdyn*	Prodynorphin or Proenkephalin B	Neuropeptide (opioid polypeptide hormone)	Leu-enkephalins compete with and mimic the effects of opiate drugs. Role in pain perception and responses to stress	https://www.uniprot.org/uniprot/O35417
*Penk*	Preproenkephalin	Neuropeptide (endogenous opioid polypeptide hormone)	Met- and leu-enkephalins have a role in a number of physiologic functions, including pain perception and responses to stress	https://www.uniprot.org/uniprot/P22005
*Pmch*	Pro-melanin concentrating hormone	Neuropeptide	Regulation of food intake and arousal	[11, 12]; https://www.omim.org/entry/176795?search=Pmch&highlight=pmch
*Pnoc*	Prepronociceptin	Neuropeptide; precursor of neuropeptides nociceptin, nocistatin, and orphanin	Nociceptin may act as a transmitter in the brain by modulating nociceptive and locomotor behavior. May be involved in neuronal differentiation and development	https://www.uniprot.org/uniprot/Q64387
*Sst*	Somatostatin	Neuropeptide; protein marker of inhibitory interneurons	It inhibits the secretion of pituitary hormones and affects the neurotransmission and cell proliferation by interacting with G-protein coupled receptors	https://www.ncbi.nlm.nih.gov/gene/6750/ortholog/?scope=89593
*Tac1*	Tachykinin 1	Neuropeptide	Modulation of neuronal activity	[13, 53]
*Tac2*	Tachykinin 2	Neuropeptide	Modulation of neuronal activity	[13, 53]
*Trh*	Thyrotropin-releasing hormone	Neuropeptide	Role in the regulation and release of thyroid-stimulating hormone, as well as prolactin Neurotransmitter/ neuromodulator in the central and peripheral nervous systems	https://www.uniprot.org/uniprot/P01150; https://www.ncbi.nlm.nih.gov/gene/7200/ortholog/?scope=7776
*Dlk1 (Peg9; Pref1)*	Delta-like protein 1	Non-canonical Notch ligand	Regulator of proliferation/differentiation processes	[14–16]
*Nptx2*	Neuronal pentraxin-2	Pentraxin family	Synaptic plasticity; glutamatergic signaling	[17, 54]; https://www.ncbi.nlm.nih.gov/gene/4885/ortholog/?scope=7776
*Nnat*	Neuronatin	Proteolipid	It may be involved in the regulation of ion channels during brain development	https://www.ncbi.nlm.nih.gov/gene/4826/ortholog/?scope=9347
*Chrm3*	Cholinergic receptor, muscarinic 3	Receptor (cholinergic receptor)	Regulation of acetylcholine in the central and peripheral nervous system	https://www.ncbi.nlm.nih.gov/gene/1131/ortholog/?scope=7776
*Gpr101*	G protein-coupled receptor 101	Receptor	Unknown function	https://www.ncbi.nlm.nih.gov/gene/83550/ortholog/?scope=7776
*Erbb4*	Erb-b2 receptor tyrosine kinase 4	Receptor of neuregulin	Role in neuronal development (migration of GABAergic neurons, anon navigation)	https://www.omim.org/entry/600543?search=Erbb4&highlight=erbb4#13
*Irs4*	Insulin receptor substrate 4	Receptor	Role in induline/IGF1 signaling pathways; role in growth, reproduction, and glucose homeostasis	[18, 55]; https://www.uniprot.org/uniprot/O14654
*Trhr*	Thyrotropin-releasing hormone receptor	Receptor (hormone receptor)	Receptor for thyrotropin-releasing hormone	https://www.ncbi.nlm.nih.gov/gene/7201/ortholog/?scope=89593
*Peg10*	Paternally expressed 10 protein	RNA binding protein	Role in cell proliferation, differentiation, and apoptosis	https://www.ncbi.nlm.nih.gov/gene/23089/ortholog/?scope=89593
*Ntng2*	Netrin G2	Subfamily of synaptic cell adhesion proteins	Role in neuronal migration and axonal guidance, as well as in synaptic development and transmission	https://www.omim.org/entry/618689
*Barhl2*	BarH-like 2 homeobox protein	Transcription factor (BarH family of homeodomain proteins)	DNA binding activity. Potential role in regulation of transcription by RNA polymerase II. Role in neuronal differentiation	[19, 56]; https://www.ncbi.nlm.nih.gov/gene/343472
*Bcl11b*	B cell leukemia/lymphoma 11B	Transcription factor (zinc-finger transcription factor family)	Involved in central nervous system development and adult neurogenesis	[20, 57]
*Ebf3*	Early B cell factor 3	Transcription factor (early B cell factor (EBF) family of DNA binding transcription factors)	Role in neuronal differentiation and brain patterning	[21]
*Fezf2*	Fez family zinc finger 2	Transcription factor	Role in nervous system development (e.g., rostro-caudal patterning). Regulation of neuron differentiation	[22, 58]; https://beta.uniprot.org/uniprotkb/Q9ESP5/entry; https://www.ncbi.nlm.nih.gov/gene/55079
*Foxg1*	Forkhead box G1	Transcription factor (fork-head transcription factor family)	Development of telencephalon	[23, 59]
*Foxp2*	Forkhead box P2	Transcription factor (forkhead box P (FOXP) subfamily)	Putative roles in the nervous system such as modulation of synaptic plasticity, neurodevelopment, or neural transmission. Involved in language and speech development	[24, 60]
*Lhx5*	LIM homeobox protein 5	Trascription factor	Implicated in forebrain development	[25, 61]
*Lmx1a*	LIM homeobox transcription factor 1 alpha	Transcription factor (LIM homeobox family)	Positive regulator of insulin gene transcription. Implicated in the formation of roof plate and roof plate-derived structures. Role in the development of dopaminergic neurons	[26, 62]; https://www.ncbi.nlm.nih.gov/gene/4009/ortholog/?scope=7776
*Meis2*	Myeloid ecotropic insertion site 2	Trascription factor (TALE “three amino acid loop extension” family of homeodomain-containing proteins)	Brain patterning; adult neurogenesis	[27, 63]; https://www.ncbi.nlm.nih.gov/gene/17536
*Otp*	Orthopedia homeobox	Transcription factor (homeodomain family)	Implicated in differentiation of specific neuroendocrine lineages within the hypothalamus	[28, 29]
*Pitx2*	Paired-like homeodomain transcription factor 2	Transcription factor (RIEG/PITX homeobox family)	Implicated in neuronal differentiation, migration, and axon outgrowth	[30, 31, 64, 65]; https://www.ncbi.nlm.nih.gov/gene/5308/ortholog/?scope=7776
*Plagl1 (Zac1)*	PLAG (pleomorphic adenoma gene-like) zinc finger 1	Transcription factor (zinc finger protein)	Regulation of telencephalic development	[32, 66]
*Rfx4*	Regulatory factor X, 4	Transcription factor (helix-turn-helix)	Role in early brain development	https://www.uniprot.org/uniprot/Q33E94; https://www.ncbi.nlm.nih.gov/gene/71137/
*Satb2*	Special AT-rich sequence binding protein 2	Trascription factor (special AT-rich binding protein family)	Cortical neuron differentiation	[33, 67]
*Sim1*	Single-minded family bHLH transcription factor 1	Transcription factor (basic helix-loop-helix-PAS domain transcription factor)	Implicated in differentiation of specific neuroendocrine lineages within the hypothalamus	[34–36]
*Tbr1*	T-box brain transcription factor 1	Transcription factor (T-box transcription factor family)	Involved in multiple aspects of cortical development, including neuronal migration, laminar and areal identity, and axonal projection	https://www.ncbi.nlm.nih.gov/gene/10716
*Zic5*	Zinc finger protein 5	Transcription factor (Zic family)	Role in neural crest development and neural tube closure	[37, 68]
*Prph*	Peripherin	Type III intermediate filament protein	Potential role in neurite elongation during development and axonal regeneration after injury, but its exact function is unknown; associated with susceptibility to amyotrophic lateral sclerosis	https://www.genecards.org/cgi-bin/carddisp.pl?gene=PRPH#function; https://www.ncbi.nlm.nih.gov/gtr/genes/5630/

**Table 2:**
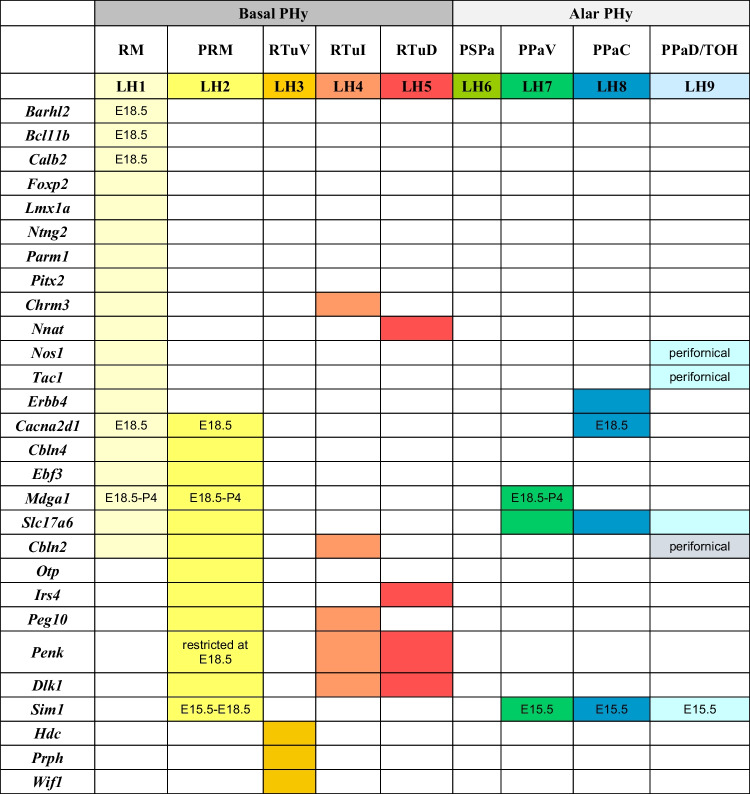

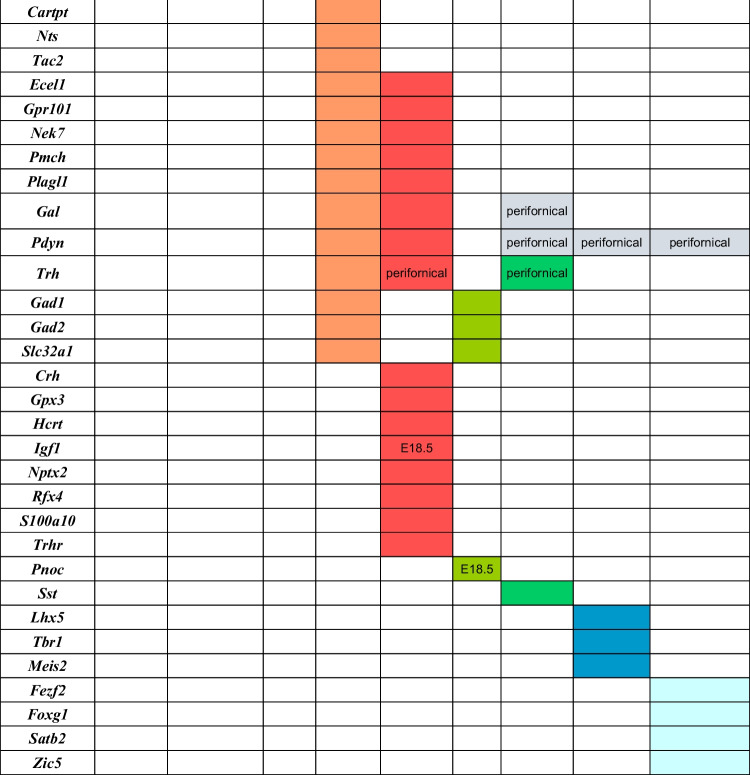
Adult expression of 59 gene markers across the ventrodorsal subdivisions of the lateral hypothalamus (LH) and their correlation with basal and alar subdivisions of the peduncular hypothalamus (PHy). Color-coded entries represent large subpopulations of positive cells for the indicated markers at the specified LH sectors. The gray boxes represent less numerous positive perifornical subpopulations at the indicated loci. Sparse positive cells, such as Otp-positive cells within adult sectors LH7–LH9, are not shown. Some markers are selectively expressed at the indicated embryonic stages rather than in the adult. Correlation of LH sectors to ventrodorsal progenitor areas of PHy is marked at the top. *PPaD/TOH*, dorsal peduncular paraventricular/telencephalo-opto-hypothalamic domain; *PPaC*, central peduncular paraventricular domain; *PPaV*, ventral peduncular paraventricular domain; *PSPa*, peduncular subparaventricular domain; *PRM*, periretromamillary domain; *RM*, retromamillary domain; *RTuD*, dorsal retrotuberal domain; *RTuI*, intermediate retrotuberal domain; *RTuV*, ventral retrotuberal domain

Moreover, we include some mouse brains treated by ISH followed by immunohistochemistry (IHC) from our histological collection. A series of transverse sections through the peduncular hypothalamus were treated by ISH for *Nkx2.2* and immunoreaction against tyrosine hydroxylase (TH) antibody at P4 postnatal stage. Other series of horizontal sections through the adult hypothalamus (conventional coronal sections) were hybridized for *vGlut2* (*Slc17a6*; potentially glutamatergic cells) and for *Gad67* (*Gad1*; potentially GABAergic cells) and further immunoreacted with a TH antibody.

### Animals

All experimental procedures, experimental protocols, handling use, and care of mice were conducted in compliance with the current normative standards of the European Community (86/609/EEC), the Spanish Government (Royal Decree, 1201/2005; Law 32/2007) and the approval of University of Murcia Committee for Animal Experimental Ethics.

### Preparation of Tissue

Mice were anesthetized with 2% chloral hydrate/PBS, and perfused intracardially with cold 4% paraformaldehyde/PBS. Following dissection, brains were postfixed in the same solution for 3 h to overnight. All tissues were either sectioned directly or stored at – 20 °C in a solution of 30% vol/vol ethylene glycol, 30% vol/vol glycerol, 0.4 × PBS. Brains stored in this way were rinsed overnight in PBS prior to sectioning.

### Sectioning of Tissue

For sectioning, the embedded tissue was oriented to obtain either sagittal, horizontal, or transverse sections across the hypothalamus, and cut into series of 120-µm-thick vibratome sections obtained from 6% agarose blocks. Sections were processed for ISH (*Nkx2.2*, *vGlut2*, *Gad67*) followed by IHC detection of tyrosine hydroxylase (TH).

#### Immunohistochemistry

The immunohistochemical reaction protocol was described in detail by Ferran, et al. [69]. A tyrosine hydroxylase antibody was used (TH; Novus, Cat# NB300-109 RRID: AB 10,077,691, dilution 1:1000). The primary antibody was incubated overnight at 4 °C. After washes, the sections were incubated with biotinylated goat anti-rabbit antibody (Vector Laboratories, CA; used at 1:200 dilution) followed by a streptavidin–peroxidase complex (Vectastain-ABC kit; Vector Laboratories; 0.001% dilution), applied for 1 h at room temperature. Peroxidase activity was developed with 0.03% 3,30-diaminobenzidine (Sigma; St Louis, MO), plus 0.003% hydrogen peroxidase. After immunohistochemical and hybridization labeling, the slides were washed several times in PBS, air-dried, and cover slipped with Cytoseal 60 (Thermo Scientific, Ref. 8310–16) or Mowiol (Calbiochem, Bad Soden, Germany, Ref. 475,904). We verified the specificity of the antibodies by performing parallel control experiments that omitted the primary antibody, checking that no residual immunostaining was detected (data not shown). Immunoreaction shown in Fig. 8A was obtained with a pan-distalless (DLX) polyclonal antibody whose specifications are described by Puelles et al. [40]; this figure corresponds to their Fig. 8.15E.

### Reverse Transcriptase-Polymerase Chain Reaction

*Gad67* (*Gad1*), *Nkx2.2*, and *vGlut2* (*Slc17a6*) cDNA fragments were obtained by reverse transcription (RT). RNA was individually extracted with Trizol reagent (Invitrogen, Carlsbad, CA, Cat. 10,296–028) from freshly dissected brains of *Mus musculus*. The RNA was treated with DNase I (Invitrogen, Cat. 18,068–015) for 15 min at room temperature (RT), and the enzyme was then inactivated at 65 °C. Afterwards, RNA samples were converted to single-stranded cDNA with Superscript III reverse transcriptase (Invitrogen, Cat. 18,080–044) and oligo-dT-anchored primers.

The PCR conditions used were an initial denaturation step at 94 °C for 5 min, then 35 cycles (30 s at 94 °C, plus 1 min at Tm temperature (58 °C), and 1 min at 72 °C), followed by 20 min at 72 °C. The PCR products were cloned into the pGEM-T Easy Vector (Promega, Cat. A1360), and sequenced (SAI, University of Murcia).

### In Situ* Hybridization*

Brain sections were processed for in situ hybridization with digoxigenin-UTP-labeled antisense riboprobes. Sense and antisense digoxigenin-labeled riboprobes for mouse *Gad67*, *Nkx2.2*, and *vGlut2* were synthesized following the manufacture’s recommendations (Roche Diagnostics S.L., Applied Science, Barcelona, Spain), and applying specific polymerases (Fermentas, Madrid, Spain). Probe sequence information is: *Nkx2.2* (NCBI accession number U31566.1, bp size 2,018, position 1–2018 J from Rubenstein’s lab); *Gad67* (bp size 2,000, full coding sequence from Bryan Condi; see Long et al. 2009; their Table 1) and *vGlut2* (bp size 305, 305 from 3′ end, from Robert Edwards; see Long et al. 2009; their Table 1).

In situ hybridization (ISH) was performed as described by Ferran et al. [69]. After hybridization, all sections were washed and incubated in a solution containing alkaline phosphatase coupled anti-digoxigenin antibody (diluted 1:3.500; Roche Diagnostics). Nitro blue tetrazolium/5-bromo-4-chloro-3-indolyl phosphate (NBT/BCIP; Roche) solution was then used as chromogenic substrate for the final alkaline phosphatase reaction (Boehringer, Mannheim, Germany). No specific signal was obtained with sense probes (data not shown).

## Results

Our description of the diverse LH cell populations found associated with the medial forebrain bundle is based on material fixed at an advanced embryonic stage (E18.5) or at postnatal to adult stages (P4–P56). Data on earlier embryonic stages were included exceptionally to clarify the potential sources of given cell populations. The 59 genes selected from the Allen Developing or Adult Mouse Brain Atlases are listed in Table 1, where protein type and associated functions are specified. Table 2 shows the markers grouped by shared combinatorial properties among the ventrodorsal histogenetic subdivisions defined along the length of the medial forebrain bundle.

The conventional LH, defined as lying interstitial to the medial forebrain bundle, is in fact restricted to the peduncular hypothalamus (PHy), as was previously suggested implicitly by Nieuwenhuys et al. [18], without referring to prosomeres. As shown in Fig. 1, in the prosomeric model the PHy is the hypothalamic portion of the hypothalamo-telencephalic prosomere hp1, constituting the caudal anteroposterior part of the hypothalamus, lying next to its caudal neighbor, the prethalamic diencephalon. The PHy is notoriously traversed (dorsoventrally, according to prosomeric analysis) by numerous ascending and descending non-myelinated or poorly myelinated fiber packets that interconnect the telencephalon and hypothalamus, as well as other diencephalic, mesencephalic and brainstem regions [18]. This abundant set of heterogeneous components constitutes the medial forebrain bundle (mfb), which courses deep to the cerebral peduncle, or lateral forebrain bundle (pe/lfb; Fig. 1) and finally largely enters the basis and the medial wall of the hemisphere.

The PHy, and therefore the LH, is bounded caudally by the prethalamus and rostrally by the terminal hypothalamus (THy); the latter represents the hypothalamic portion of the hypothalamo-telencephalic prosomere hp2 (Figs. 1 and 2A; [17, 41]). The PHy/THy intrahypothalamic border (IHB) courses in front of the hypothalamic trajectory of the fornix tract (f), a myelinated tract that courses dorsoventrally (transversally) through the rostral part of PHy (Fig. 1). The fornix tract originates in the hippocampal commissural area, crosses the septum, and enters the PHy behind the anterior commissure (in front of the interventricular foramen), to course dorsoventrally through its alar and basal regions, reaching finally the retromamillary commissure in the PHy floor, after giving collaterals or terminals to the mamillary region, a basal component of THy [26]. Dorsally the PHy (including the LH) contacts the substantia innominata of the evaginated telencephalon, apparently through the recently identified transitional *telencephalo-opto-hypothalamic* longitudinal domain (TOH; Fig. 1; [37, 70]).

Figure 2 shows the radial organization of the PHy in a series of topological *horizontal* sections parallel to the prosomeric forebrain axis (note these are conventionally described as “coronal sections” under the columnar paradigm; see also the corresponding radial glial structure in Puelles et al. [17]; their Fig. 8.12). Following the standard developmental mantle radial stratification schema of Senn [71], the PHy appears subdivided radially into periventricular, intermediate, and superficial strata (Fig. 2B–J); the intermediate stratum is habitually divided into cell-dense medial hypothalamus (MH) and cell-sparse LH [15, 17] (the MH/LH limit roughly coincides with the fornix tract). The pial surface of PHy is oriented ventrolaterally (due to bending of its radial glial processes around the VM nucleus) and is larger than the ventricular surface. This radial bending of PHy around the VM is the reason why the classic LH is actually restricted to the PHy. It should be noticed that the cerebral peduncle (jointly with the underlying subthalamic nucleus at retrotuberal levels) only covers in horizontal sections the caudal two-thirds or so of the LH; the rostral third of LH (which contains the fornix and the nigrostriatal tract) extends into the brain surface rostral to the peduncle, in the superficial area where the parasubthalamic nucleus is found (purple area in Fig. 2B–J). The subpial optic tract crosses orthogonally the subparaventricular alar domain of PHy, close to the alar-basal boundary [72], so that its topologically ventral rim serves as an approximate landmark of the alar-basal transition, relevantly for the topologic analysis of LH components.

Our description of LH will be thus restricted to the outer intermediate stratum of PHy, which contains by definition the LH populations of interest, adding the rostral portion where the bent LH seems to extend into the superficial stratum. The standard LH populations that lie interstitial to the mfb at various horizontal section levels are highlighted in blue in Fig. 2B–J, including various alar and basal perifornical cell groups (labeled Pf), whereas the associated medial and superficial populations are highlighted in yellow and purple, respectively.

For simplicity, each LH population was named with the acronym LH and numbered consecutively in ventrodorsal order (Fig. 2A). According to our analysis, the LH extends through five basal regions (LH1-LH5, corresponding to the progenitor domains RM, PRM, RTuV, RTuI, RTuD; [17]) and four alar regions (LH6-LH9 corresponding to the progenitor regions PSPa, PPaV, PPaC, PPaD; *loc. cit*.; PPaD is equivalent to the TOH of Morales et al. [37]. Multiple molecularly different neuronal populations can be thus distinguished and classified in an embryologically relevant DV pattern [73, 74].

### Basal Lateral Hypothalamic Populations

Selected markers expressed in the basal LH subregions were downloaded from ISH data at the Allen Developing Mouse Brain and Allen Mouse Brain atlases, which are illustrated in Figures 3, 4, 5, 6 and 7 and summarized in Table 2. Our description follows a ventrodorsal order, that is, from LH subregion 1 (LH1) upwards to LH subregion 5 (LH5) (see Fig. 2A, B–F).

### LH1: the Retromamillary Subdomain

We estimate that the ventralmost LH region, or LH1, lies within the basal RM area, the ventralmost subregion of basal PHy. The fornix tract, the landmark of both the intrahypothalamic boundary and the limit between medial and lateral hypothalamus, reaches in its dorsoventral course through the rostral part of PHy the retromamillary decussation in the local floor plate [26]. The RM area lies ventral to the PRM subdomain, and both are rostral to the diencephalic p3 tegmentum occupied by the rostralmost part of the substantia nigra complex (p3teg, SNC, SNR; Figs. 1 and 2B). The RM contacts rostrally with the mamillary area, the topologically equivalent basal area within THy (M; Figs. 1, 2B, and 3). Conventionally, the RM region displays cytoarchitectonically medial, lateral, and superficial RM populations (RMM, RML, RMS; Figs. 2B and 3C, L, F, H–L), according to the stratigraphic schema of Puelles et al. [17]. The mamillotegmental tract courses caudalwards roughly through the boundary between RMM and RML (mtg; e.g. Figs. 2B and 3B). Our LH1 thus corresponds to RML, irrespective that classical neuroanatomic studies did not visualize it as a part of LH. At some section levels the RML/LH1 extends caudally into a molecularly analogous *retro-subthalamic cell population* (RSTh) ([24]; their Fig. 36-T15, T16, 37-S2, S3, 38-H4-H6) which might belong to the prethalamic tegmentum (Figs. 2C and 3A, B, E).

The whole RM including its LH1 component contains mainly *Slc17a6*-expressing cells reflecting the glutamatergic character of its population (Tables 1 and 2; Puelles et al., 2012); the *Slc17a6* gene encodes the vGLUT2 transporter. Several other adult transcripts such as *Tac1* (encodes tachykinin 1), *Chrm3* (encodes the muscarinic 3 cholinergic receptor), *Pitx2* (encodes the paired-like homeodomain transcription factor 2), *Nnat* (encodes neuronatin), *Cbln4* (encodes cerebellin 2 precursor protein), and *Erbb4* (encodes Erb-B2 receptor tyrosine kinase 4) are expressed in LH1 (Fig. 3). LH1 also contains retromamillary perifornical cells expressing the same markers. The neighboring mamillary area largely lacks these cell phenotypes (Fig. 3A, B, D–F, J–L). *Tac1*- and *Chrm3-*positive cells are found also in the neighboring possibly prethalamic retro-subthalamic nucleus (RSTh; Fig. 3A, B, E). *Tac1*-, *Chrm3*-, *Pitx2*-, *Nnat*-, and *Erbb4*-expressing cells are likewise distinguished in the adjacent p3 tegmentum (p3tg; Fig. 3). These coincidences might be due to early embryonic migratory translocation of RM cells into p3tg. Other non-illustrated molecular markers also selectively expressed in LH1 cell subpopulations (Table 2), encode transcription factors (*Barhl2*, *Foxp2*, *Lmx1a*), calretinin (*Calb2*), netrin (*Ntng2*), and an endoplasmic reticulum molecule (*Parm1*), whereas other transcripts apart of *Slc17a6* are expressed both in LH1 and in neighboring LH2 (e.g., *Cacna2d1*, *Cbln2*, *Cbln4*, *Ebf3*, *Mdga1*; Table 2; Fig. 4G–I).

Interestingly, numerous LH1 markers which encode transcription factors (*Bcl11b, Lmx1a*, *Foxp2*, *Pitx2*), calretinin (*Calb2*), a calcium channel (*Cacna2d1*), cerebellins (*Cbln2*, *Cbln4*), a cholinergic receptor (*Chrm3*), nitric oxide synthase 1 (*Nos1*), netrin (*Ntng2*), a vesicular glutamate transporter (*Slc17a6*) and tachykinin 1 (*Tac1*), are found in the ventral premamillary nucleus (see Fig. 3A, D, G) and/or the subthalamic nucleus (not shown). Cells of these tangentially migrated nuclei reportedly originate at the RM area according to several experimental studies [76–82].

### LH2: the Periretromamillary Subdomain

LH2 is the LH subregion associated with the periretromamillary (PRM) basal subdomain. This subdomain was first defined molecularly by Puelles et al. [17] as associated with selective *Otp* and *Sim1* basal plate signals, unrelated to alar expression domains of the same markers (see also [82–85].

At early stages, *Irs4* expression (encoding insulin receptor substrate 4) accompanies *Otp* and *Sim1* transcripts in the characterization of the PRM (Fig. 4A–C); *Otp*-expressing cells continue to be detected in LH2 at least until P28 (see Allen Developing Mouse Brain Atlas). Classically the PRM was wrongly lumped with the neighboring diencephalic tegmentum (largely devoid of *Otp* expression) under the now outdated concept of “posterior hypothalamus” (e.g., [86]; his Figs. 13–18, reproduced in Puelles et al. [17]; their Fig. 8.3). The PRM appears divided into the main PRM mass (representing the medial hypothalamic stratum), and the LH2 domain interstitial to the fornix and nigrostriatal tracts among other medial forebrain bundle components (f, ns; Fig. 4; right upper part). The LH2 ends superficially rostrally to the subthalamic nucleus and the peduncle, at levels coincident with the parasubthalamic nucleus under the lateral tuberal nucleus (LH2 in blue; STh, PSTh, and TuL in purple; pe (in gray); see schema inserted at the upper right corner of Fig. 4; note also the curved radial boundary between THy and PHy).

The PRM subdomain as a whole contains mainly *Slc17a6*-expressing glutamatergic cells, like the subjacent RM domain (Table 2; [17]. *Penk-*expressing cells are also present within the periretromamillary LH2, with additional positive cells in suprajacent LH cell subpopulations of the basal RTu domains and dispersed LH cells at the alar PSPa/PPa domains (LH4, LH5; Fig. 4D–F; Table 2; low expression at LH6–LH8 was not included in Table 2). Whereas a *Penk* population appears restricted to LH2 at E18.5 (Fig. 4D), in the adult, many *Penk-*positive cells are dispersed within other LH subregions, particularly LH4 (Fig. 4E; Table 2). In contrast, the subjacent LH1 has few *Penk*-expressing cells; *Penk* encodes the neuropeptide preproenkephalin. *Ebf3* and *Mdga1*, jointly with other markers such as *Cacna2d1*, *Cbln2*, and *Cbln4* are also expressed in LH2 and LH1 cells (Table 2); the suprajacent RTu region encompassing LH3–LH5 largely lacks *Ebf3* (encoding early B cell transcription factor 3) and *Mdga1* (encoding MAM domain containing glycosylphosphatidylinositol anchor 1) signals (Fig. 4G–I). Moreover, at both E18.5 and P56, numerous *Peg10-*positive cells (*Peg10* encodes paternally expressed gene 10) are found at the LH2, a pattern shared also with LH4, but not with LH1 (Fig. 4J–L). Various other markers such as *Dlk1* and *Irs4* (not shown) are mainly restricted to LH2 (Table 2).

### LH3: the Ventral Retrotuberal Subdomain

Puelles et al. [17] identified the basal PHy territory stretching from the PRM to the alar-basal boundary as a retrotuberal domain (RTu), referring to the larger THy tuberal (Tu) domain counterpart (Fig. 1). Both Tu and RTu were subdivided dorsoventrally into parallel ventral, intermediate, and dorsal subdomains (RTuV, RTuI, RTuD in the case of RTu; [17]). These three subdomains contain respectively our LH3–LH5 basal components of the LH. LH3 represents the ventral retrotuberal element. RTuV is a thin subdomain limiting dorsally the PRM. Jointly with its TuV counterpart, it was distinguished by Puelles et al. [17] as the apparent source of histaminergic *Hdc*-expressing neurons, which later spread partially into neighboring tuberomamillary, retromamillary, and mamillary areas (Fig. 5A–D; Table 2; Allen Developing Mouse Brain Atlas). *Hdc* cells are first scattered from periventricular to subpial surfaces within the ventral retrotuberal PHy (including our LH3 at intermediate radial levels) as well as in the correlative TuV parts of THy (Fig. 5C, D). Contrarily, the intermediate and dorsal components of the RTu/Tu domains, as well as the PRM, largely lack *Hdc* mRNA expression. However, migrated *Hdc*-positive cells later appear in the terminal perimamillary area rostral to PRM, and subpially in the RM/M domains (Fig. 5A, B). Both RTuV and TuV domains otherwise produce mainly GABAergic cells, probably due to local expression of the *Dlx* genes, which contrasts with the underlying PRM/PM domain that displays a glutamatergic profile [17].

According to previous descriptions, histaminergic cells express the genes *Prph* (encoding peripherin) and *Wif1* (encoding WNT inhibitory factor 1) [43, 87, 88], two markers absent in our AGEA analysis of ISH Allen data, though they do appear in that database. We confirmed that *Prph and Wif1* are expressed in LH2 cells with a similar distribution as *Hdc* cells (Fig. 5E, F), and they were added to Table 2.

### LH4: the Intermediate Retrotuberal Subdomain

LH4 is the LH component of the intermediate RTu subdomain (RTuI). In horizontal sections through the adult hypothalamus (conventional coronal sections), the LH4 level is recognized by landmarks such as the subthalamic and dorsomedial hypothalamic nuclei (in PHy) and the ventromedial and arcuate complexes (in THy; Fig. 6D). There are abundant *Gad1* (*Gad67*)- and *Gad2* (*Gad65*)*-*expressing GABAergic cells in LH4 (Table 2; see below). LH4 subpopulations express neuropeptides such as *Cartpt*, *Nts*, *Penk*, *Pdyn*, and *Tac2*, and other markers such as *Cbln2*, *Ecel1*, *Nek7*, and *Peg10* (see Fig. 6), as well as other non-illustrated molecular markers encoding transcription factors (*Plagl1*), neuropeptides (*Gal*, *Pmch*, *Trh*), receptors (*Chrm3*, *Gpr101*), and other markers such as *Dlk1* (Table 2).

*Nts* and *Tac2* encode the neuropeptides neurotensin and tachykinin 1, respectively. *Nts-*positive cells mainly localize in the central part of LH4, contrasting with *Tac2*-expressing cells, which appear in more medial and lateral locations (compare Fig. 6A–C). However, a subgroup of *Nts*-positive cells overlaps or intermixes with *Tac2*-positive cells next to the hypothalamo-diencephalic border (red asterisks; Fig. 6B, C). Neuropeptidergic *Cartpt*-expressing cells are largely restricted to LH4, though a few positive cells are found in the suprajacent LH5 (Fig. 6E), as occurs also with *Peg10* cells (not shown). *Cartpt* cells are mainly located in the central part of the LH4 (Fig. 6F) whereas *Peg10* cells are more numerous and distribute widely throughout LH4 (Fig. 6G). *Ecel1*-, *Nek7*-, and *Pdyn*-expressing cells are present in LH4 (Fig. 6H–J) but are found also in the suprajacent LH5 subregion (*Pdyn* in Fig. 7K, L; Table 2). The *Pdyn* gene encodes the precursor prodynorphin neuropeptide. *Penk*-expressing cells represent other significant neuropeptidergic subpopulations of LH4 (Fig. 6K). As noted previously, in the adult *Penk* cells appear at different basal and alar LH sectors (see Table 2), though at E18.5 they are restricted to LH2 (Fig. 4D). The cerebellin family member *Cbln2* is widely expressed in LH4 (Fig. 6L).

### LH5: the Dorsal Retrotuberal Subdomain

This is the dorsalmost component of the basal lateral hypothalamus, located at the intermediate stratum of the dorsal retrotuberal hypothalamic subdomain (RTuD). The local medial hypothalamic stratum contains the posterobasal nucleus (PBas) and the A13 dopaminergic cell group [17, 89]. Horizontal sections through LH5 no longer intersect the peduncular dorsomedial hypothalamic nucleus (DM-P) or the subthalamic nucleus (STh), while the alar ventral entopeduncular nucleus (EPV) may start to be visible within the peduncle (Fig. 7C). LH5 characteristically contains large-size *Hcrt*- and *Pmch*-positive cells produced at this level (Fig. 7A, B, G–I). *Hcrt* encodes the neuropeptide hypocretin HCRT (also named orexin). Some *Hcrt* cells apparently disperse into both peduncular and terminal parts of the SPa area (PSPa and black arrows in TSPa; Fig. 7A). *Crh*-, *Gpx3*-, *Igf1*, *Irs4-*, *Nnat-*, *Rfx4*, *S100a10-*, and *Trhr-*expressing neurons are mainly restricted to LH5 (Table 2; Fig. 7D–F); other molecular markers of LH5 that spread variously out of it include *Dlk1*, *Gal*, *Pdyn*, *Penk*, *Plagl1*, and *Trh* (Table 2). At perinatal stages, an important subpopulation of RTuD-originated cells express *Pmch*, which encodes pro-melanin-concentrating hormone. These large-sized *Pmch* elements intermix with *Hcrt* cells and populate the PBas nucleus (medial hypothalamus) and the LH5, and partly disperse into the subjacent LH4 and even the neighboring prethalamic zona incerta at later stages (Fig. 7G–I; Table 2). These cells are accompanied by peptidergic *Gal*, *Pdyn*, and *Penk* cells (Fig. 7J–M; Table 2) or *Cartpt* cells (Fig. 6E). Other transcripts expressed in LH5 cells encode transcription factors (*Plagl1*), neuropeptides (*Trh*), receptors (*Gpr101*, *Trhr*), enzymes (*Ecel1*), and other molecules (*Dlk1*, *Nek7*) (Table 2).

### Alar Lateral Hypothalamic Populations

**Fig. 10:**
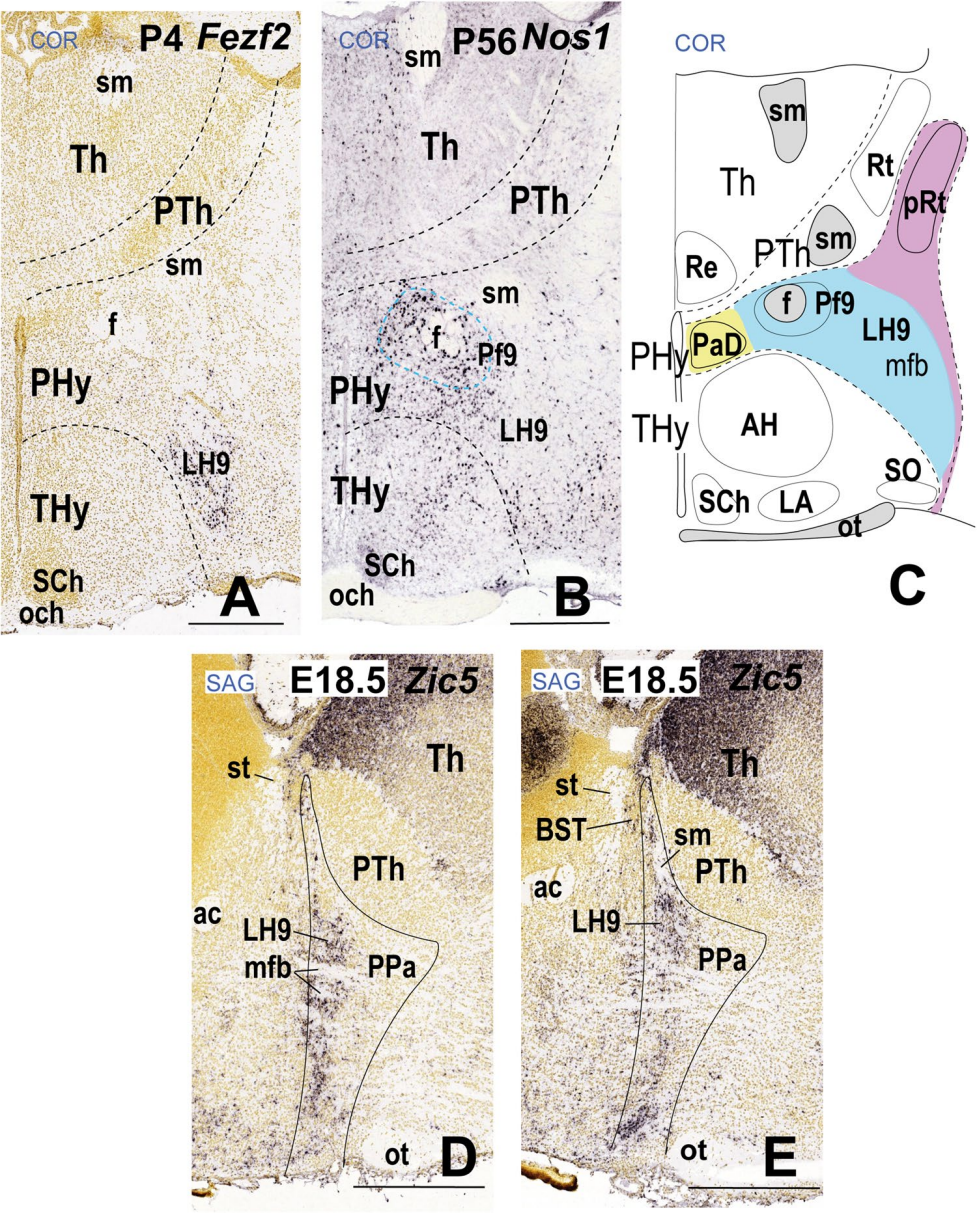
Panel illustrating other markers expressed at the LH9 at various stages. LH9 is part of the dorsal peduncular paraventricular domain (PaD), which apparently coincides with the newly defined *Foxg1*-positive telencephalo-opto-hypothalamic area (TOH; Morales et al. [37]). Sagittal and coronal sections (marked as SAG or COR at the upper left corner) were downloaded from the Allen Developing Mouse Brain Atlas. The sagittal sections show a restricted dorsoventral topography of the labeled LH9 cells, whereas the coronal sections demonstrate the corresponding either diffuse or restricted topography within the LH9 level as defined in Fig. 2 and panel **C**. Some markers are shown only in coronal sections (with corresponding level landmarks). (**A**) Coronal section illustrating a *Fezf2*-positive LH9 subpopulation that lies adjacent to the intrahypothalamic boundary (PHy/THy) at P4. (**B**) Coronal section showing a perifornical *Nos1*-positive LH9 subpopulation at P56. (**C**) Schematic representation of the LH9 sector (in blue) in a coronal/horizontal section (extracted from Fig. 2) through the dorsalmost alar peduncular hypothalamus, transitional into telencephalon; the corresponding atlas section clearly was oblique, so that paraventricular structures are seen only in PHy, whereas underlying subparaventricular structures represent the THy. The local medial and superficial peduncular paraventricular components are highlighted in yellow and purple, respectively. (**D**, **E**) Two sagittal sections showing LH9 *Zic5*-positive cells at E18.5. Scale bars: (**A**) = 500 µm; (**B**, **D**, **E**) = 600 µm. For additional abbreviations, see “Abbreviations”

The following analysis of alar LH subpopulations (Fig. 1 and 2G–J) is also based mainly on ISH data from the Allen Mouse Brain Atlas. Table 2 summarizes the cell phenotypes found in each alar LH subregion. The expression of selected markers in alar LH subregions is illustrated in Figs. 8, 9, and 10.

### LH6: the Subparaventricular Subdomain

The LH6 subpopulation belongs to the peduncular alar SPa domain (PSPa), which is continuous caudalwards with the prethalamic rostral liminar band (RLi; Fig. 8A; Puelles et al., 2021). Both PSPa (including LH6 at the appropriate radial level) and its rostral counterpart TSPa contain GABAergic *Dlx5/6*- and *Arx*-expressing cells, as shown here in a sagittal section from a postnatal *Dlx5/6-LacZ* transgenic mouse whose brain was secondarily immunoreacted with a Pan-Distalless antibody (Fig. 8A). In the LH, *Dlx5/6*-expressing cells appear in the alar LH6, as well as in the underlying basal LH5 and LH4 subdivisions (possible tangential migration). LH6 contains a mixture of blue (*Dlx5/6-LacZ* β-galactosidase reaction product) and brown (DLX protein) labeled cells, as occurs partially at the neighboring basal LH5, whereas LH4 contains mainly brown immunoreacted cells. According to the analysis of such signals performed by Puelles et al. [40], the blue reaction implies persistent *LacZ* reaction possibly maintained by low levels of *Dlx5/6* enhancer activity and/or perdurance of the β-galactosidase enzyme, with low or undetectable DLX protein levels; the brown reaction represents ongoing DLX1/2/5/6 expression, and no postnatal *Dlx5/6-LacZ* enhancer activity. *Gad1* (*Gad67*), *Gad2* (*Gad65*), and *Slc32a1*, three markers of GABAergic neurons, are also expressed in LH6 cells (Table 2). At E18.5, LH6 selectively contains *Pnoc*-positive cells, mostly located superficially, at the place where the PHy bends pialwards around the THy (Fig. 8B); however, some *Pnoc*-positive cells appear dispersed within other LH subregions at postnatal mouse stages (not shown).

### LH7: the Ventral Paraventricular Subdomain

Puelles et al. [17] proposed a dorsoventral division of PPa in dorsal, central, and ventral subareas (PPaD, PPaC, PPaV) with subtly variant molecular identities (see also [89]). We found that the whole hypothalamic PPa subdomain is characterized as a *Dlx*-negative (Fig. 8B) and *Otp/Sim1-*positive region (Fig. 9B) lying above the SPa band. Consistently with the postulated PPa subareas, we identified three alar LH subregions (LH7, LH8, LH9; Figs. 1 and 2A, H-J). Some markers appear restricted to cell subpopulations located selectively at one of these dorsoventral levels.

LH7 cells are located at the ventral part of the PPa subdomain (PPaV), coinciding with the ventral and lateral paraventricular nuclei (PaV; PaL) and related superficially with the dorsal entopeduncular nucleus (EPD; interstitial to the peduncle) (see Fig. 8E). This LH part contacts caudally the prethalamic incertal complex (ZI; Fig. 8C–E). LH7 includes a subpopulation that expresses the *Sst* gene, which encodes the neuropeptide somatostatin. *Sst-*expressing cells are restricted to LH7 (Fig. 7C) whereas other markers such as *Gal*, *Mdga1*, *Pdyn*, *Sim1*, *Slc17a6*, and *Trh* are shared with neighboring LH subpopulations (see Table 2); *Gal*-, *Pdyn*- and *Trh*-positive cells are found in perifornical position, of which *Gal*-, and *Trh*-positive cells appear only at LH7 level (Table 2; see Allen Developing Mouse Brain Atlas). The whole PPa subdomain and its terminal counterpart TPa (e.g., anterior hypothalamic area, AH), have abundant *Slc17a6/vGlut2*-positive glutamatergic cells (Fig. 8D).

### LH8: the Central Paraventricular Subdomain

The LH8 subdomain corresponds to the central PPa hypothalamic area (PPaC) and is a level with the (medial) PaC nucleus and the upper part of the (superficial) EPD nucleus (Fig. 8I). LH8 contacts caudally the prethalamic reticular nucleus (Rt; Fig. 8I). *Meis2-*positive cells appear as a subpopulation of LH8. We noted that at prenatal stages *Meis2* is expressed through all radial levels of the PPa subdomain but is mainly restricted to LH8 in the adult (Fig. 8F, G; Table 2). *Meis2* encodes a homeobox transcription factor (myeloid ecotropic insertion site 2 transcription factor). The whole PPaC area essentially produces *Slc17a6* (*vGlut2*)-positive glutamatergic cells with diverse peptides (Fig. 8H; see [17, 90]). LH8 also contains a large subpopulation of cells expressing *Lhx5* and relatively less numerous *Tbr1* and *Cacna2d1* subpopulations (Fig. 8J, K, L; Table 2). The *Tbr1* subpopulation may be intrinsic or migrated early on from the prethalamic eminence, as was suggested in chick studies [91, 92]. *Lhx5* and *Tbr1* encode transcription factors whereas *Cacna2d1* encodes a voltage-dependent calcium channel. *Erbb4*-expressing cells are mainly restricted to the LH8 sector whereas perifornical *Pdyn*-positive cells are also found in LH7 and LH9 (Table 2).

### LH9: the Dorsal Paraventricular Subdomain

LH9 is the dorsal LH component within the alar PPa domain; it is noteworthy that at these section levels the fornix tract abandons its standard rostral position close to the intrahypothalamic boundary (THy/PHy) and moves progressively caudalwards within PHy until it reaches the backside of the anterior commissure and passes into the septum. As happens generally in other parts of Pa, the PPa contains glutamatergic cells expressing the *Slc17a6* (*vGlut2*) gene (Fig. 8D, H; Table 2), supposedly jointly with some peptidergic markers, and its cells express *Otp* and/or *Sim1* transcription factors (Fig. 9B, D, G). Recently this longitudinal band at the hypothalamo-telencephalic border has been identified with a hypothalamo-amygdalar corridor (HyA) vehiculating dorsal and central paraventricular cells that migrate into the medial amygdala (*Otp* cells) and pallial amygdala (*Sim1* cells) [93]. In addition, Morales et al. [37] postulated this corridor as a novel *Foxg1*-positive telencephalo-opto-hypothalamic (TOH) histogenetic area, with abundant glutamatergic neurons; the TOH is interpreted by us as a molecularly differential dorsal part of the hypothalamic paraventricular area rather than as 'telencephalic' (see “Discussion”).

We found that LH9 contains cells expressing seven transcription factors (*Fezf2*, *Foxg1*, *Otp*, *Satb2*, *Sim1*, *Zic5*; Table 2), and other markers (*Pdyn*, *Nos1*, *Tac1*; Table 2). In Fig. 9, we analyze the correlation between the hypothalamic Pa area defined by *Sim1*- and *Otp*-expressing cells, the dorsal *Nkx2.1-*negative TOH subdomain of Pa that co-expresses *Foxg1*, and the overlying diagonal/POA subpallial domains, characterized by *Nkx2.1*- and *Dlx5/6*-expressing cells (see also Fig. 8A). We found that the transcription factor *Satb2* is expressed specifically in a distinct neuronal subpopulation within the TOH area, which participates also of the *Foxg1* signal, and lacks *Nkx2.1* signal (Fig. 9A–H). As observed in sagittal and coronal sections at E18.5, *Satb2* signal appears both in a dense periventricular island and in an ampler population dispersed widely within the LH9 traversed by the medial forebrain bundle (Fig. 9H, I). The dense periventricular island may correspond to a TOH subpopulation identified as the posterior BSTM (BSTMp) by Morales et al. [37].

*Fezf2*, *Nos1*, and *Tac1* are also selective markers distinguishing LH9 cells from the neighboring LH populations at postnatal stages (Table 2). *Fezf2*-expressing cells localize next to the intrahypothalamic boundary (Fig. 10A) whereas *Nos1-*expressing cells appear in a dorsocaudal perifornical LH9 location, close to the anterior commissure (Fig. 10B; compare the schematic horizontal section through LH9 level in Fig. 10C). Curiously, the same perifornical LH9 subpopulation lacks *Fezf2*-positive cells. *Fezf 2* encodes a Fez-like zinc finger family member and *Nos1* encodes the enzyme nitric oxide synthase 1. The transcription factor *Zic5* (Fig. 10D, E) is restricted also to the LH9 sector.

### Nkx2.2-Positive Band Derivatives in the Lateral Hypothalamus

**Fig. 11:**
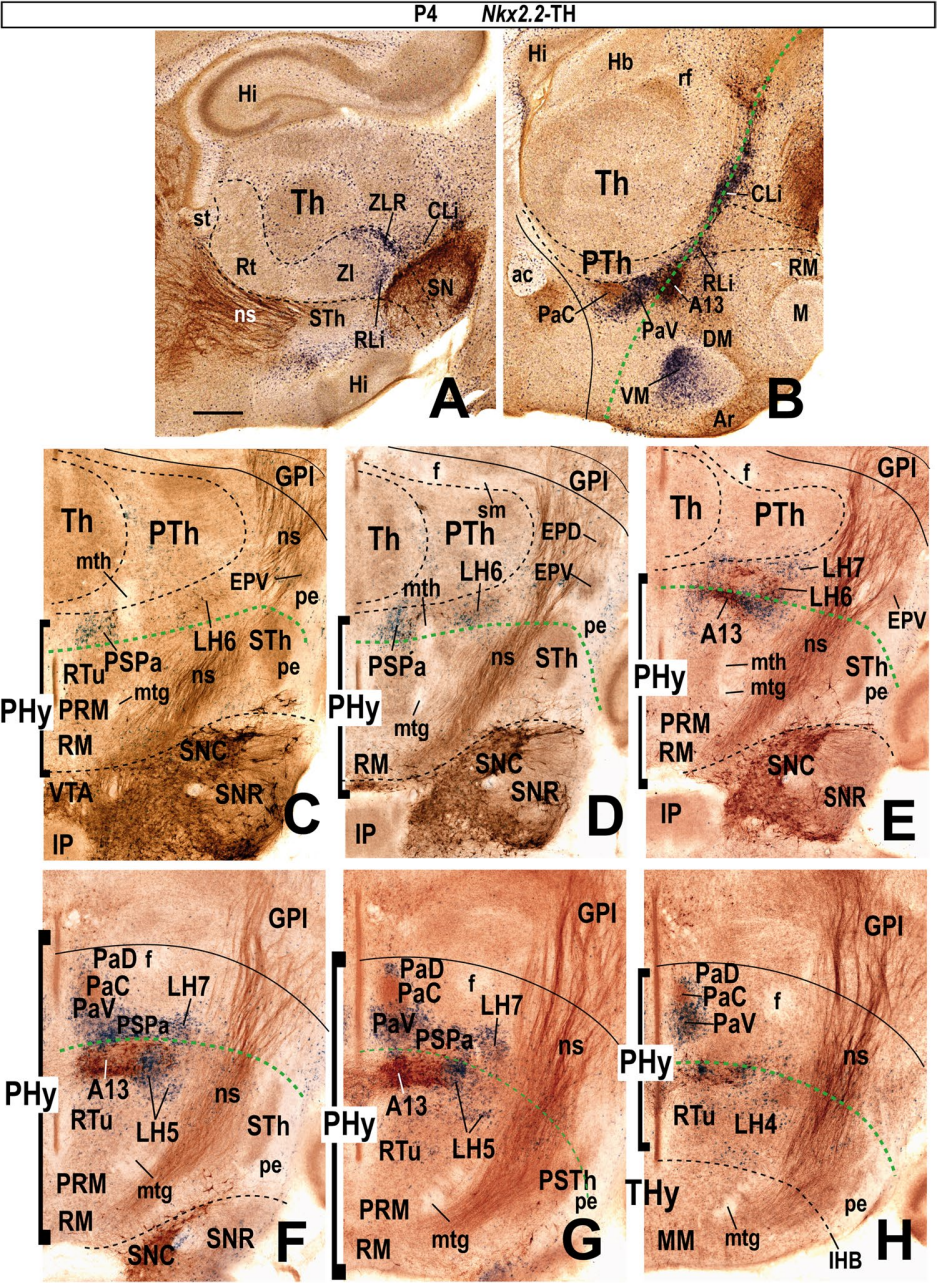
*Nkx2.2* expression in the lateral hypothalamus (LH) and other hypothalamic regions in two P4 mouse brains sectioned either sagittally (**A**, **B**) or transversally to the hypothalamus (**C–H**; that is, parallel to the peduncle). The *Nkx2.2* hybridization (dark blue) was followed by an immunoreaction with a tyrosine hydroxylase (TH) antibody (brown). The TH-positive dopaminergic fibers of the nigrostriatal tract (ns), a component of the medial forebrain bundle, contributes to the identification of LH subpopulations. The dash black lines indicate thalamo-prethalamic, prethalamo-hypothalamic, and intrahypothalamic transverse interprosomeric boundaries. The green dash line indicates the alar/basal limit. The black line marks the hypothalamo-telencephalic boundary. (**A**, **B**) *Nkx2.2*-positive cells appear in sagittal sections both in the alar paraventricular area (Pa) and the basal ventromedial nucleus (VM); in between these populations, there is a thin longitudinal periventricular *Nkx2.2*-positive population identified as the rostral liminar band (RLi; modified from Puelles et al. [40]; see their Fig. 8f, g). Reported embryonic observations suggest that both the alar and basal *Nkx2.2*-positive derivatives originate via early tangential migrations from the primary RLi locus, placed roughly along the alar-basal boundary (Puelles et al. [17]). (**C–H**) Selected transverse sections through the peduncular hypothalamus ordered from caudal to rostral. *Nkx2.2*-negative/TH-immunoreactive neurons appear in the alar LH6 subregion (**C–E**) whereas *Nkx2.2*-positive/TH-negative cells are present in the alar LH7 subregion (**E–G**); LH7 and LH6 are respectively components of the peduncular ventral paraventricular subarea (PPaV) and the peduncular subparaventricular area (PSPa). Note the basal TH-positive dopaminergic cells of the A13 group lie next to the alar/basal boundary (green dash line); *Nkx2.2*-expressing cells appear laterally to A13 within the basal LH5 (**F**, **G**); LH5 is a component of the dorsal retrotuberal area (RTuD). Disperse *Nkx2.2*-positive cells also appear in the basal LH4 subregion (**H**). Note there are also numerous *Nkx2.2*-positive cells in the dorsal and ventral paraventricular subnuclei (PaD, PaV; these are part of the medial hypothalamic stratum, not of LH). Scale bar for all images, indicated in (**A**): 500 µm. For additional abbreviations, see “Abbreviations”

The *Nkx2.2* gene is expressed early on in a longitudinal liminar band along the forebrain alar/basal limit [17, 26, 94, 95] (“liminar” comes from the Latin term “limen,” rim or border). Note this pattern is restricted to the extended forebrain tagma (including the midbrain; [96]). This gene appears expressed instead at the floor/basal border of the hindbrain and spinal cord, after an isthmic discontinuity. We examined recently the *Nkx2.2* signal at the interthalamic zona limitans shell domains related to the interprosomeric p2/p3 border, as a continuation of which the caudal and rostral longitudinal “liminar” parts of the original band can be followed (CLi, RLi; [40, 97]). These features are also recognizable at pre- or postnatal stages in other vertebrate species studied (Fig. 11A, B; [ 98–101]). *Nkx2.2*-expressing neurons derived from RLi contribute differentially to THy and PHy, including some alar and basal LH subregions (Fig. 11; [17]). The rostral liminar band (RLi) overlaps the hypothalamic alar/basal limit, thus being composed in theory of alar and basal parts (if the boundary is linear, adimensional). At early stages, different *Nkx2.2*-positive cell populations migrate tangentially from this liminar band either dorsalward (presumably the alar ones) or ventralward (the basal ones), depending on the hypothalamic prosomere. Puelles et al. ([17]; their Fig. 26) showed embryonic mouse images suggesting that the basal ventromedial hypothalamic nucleus within THy receives ventrally migrating RLi cells, while the alar paraventricular nucleus within PHy is invaded by dorsally migrating RLi cells; these enter massively the PaV subdivision, while a minor contingent moves past the PaC nucleus into the PaD formation (VM; PaV; PaD; see Fig. 11B, F–H). Analysis of a P4 series of caudorostral transverse sections through the PHy processed for *Nkx2.2* in situ hybridization (blue) followed by tyrosine-hydroxylase immunoreaction (TH; brown) (Fig. 11C–H) discloses a partial dispersion of cells expressing *Nkx2.2* into the lateral hypothalamic course of the TH-immunoreacted nigrostriatal tract (ns) coursing ventrodorsally through the medial forebrain bundle. These LH cells are observed either in basal LH4/LH5 and alar LH7 components (Fig. 11E–H; note *Nkx2.2* is expressed also by differentiating oligodendrocytes—thus some of the disperse blue cells may be oligos).

Among alar LH territories, the subparaventricular LH6 subdomain is largely *Nkx2.2-*negative and contains some TH cells (Fig. 11C–E). In contrast, the overlying LH7 (ventral paraventricular) subdomain contains *Nkx2.2*-expressing cells (Fig. 11E–G), possibly associated with the RLi elements migrated dorsally that populate the PaV and PaD subnuclei (Fig. 11F–H)*.* In the basal region of PHy, *Nkx2.2*-expressing cells were found in the LH5 and LH4 subdomains corresponding to the RTuD and RTuI areas (Fig. 11F–H); these positive cells seem to have migrated ventralwards from the *Nkx2.2-*expressing liminar RLi band; LH5 *Nkx2.2*-positive cells appear lateral to the TH-immunolabeled dopaminergic A13 population characteristically present at this dorsoventral level of medial basal PHy (Fig. 11F, G).

### GABAergic and Glutamatergic Populations in the Lateral Hypothalamus

**Fig. 12:**
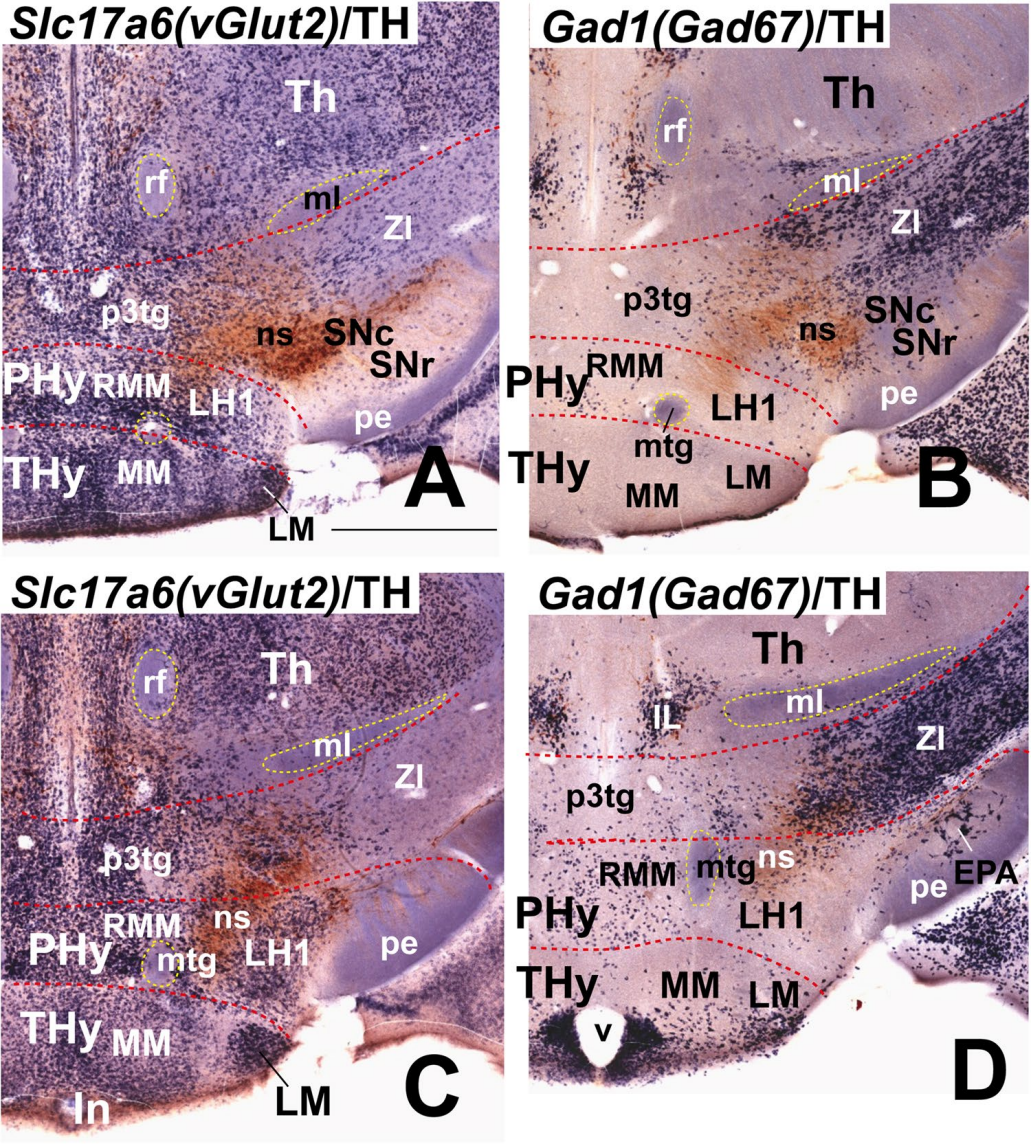
First panel of a series of four, showing the distribution of either *Slc17a6* (*vGlut2*) or *Gad1* (*Gad67*) transcripts in adjacent coronal (topologically horizontal) sections through an adult mouse hypothalamus, ordered from ventral to dorsal levels; these mappings are useful to correlate positionally glutamatergic (*Slc17a6-*) and GABAergic (*Gad1*)*-*positive cell populations. All sections were counterstained with a tyrosine hydroxylase (TH) antibody (brown reaction). The red dashed lines indicate the thalamo-prethalamic, prethalamo-hypothalamic, and intrahypothalamic transverse interprosomeric boundaries. Yellow dash lines surround some identified tracts. (**A–D**) The lateral hypothalamic subregion 1 (LH1) is illustrated in coronal sections through the retromamillary domain (RM). LH1 comprises mostly *Slc17a6-*positive glutamatergic cells. Scale bar for all images, indicated in (**A**): 730 µm. For additional abbreviations, see “Abbreviations”

**Fig. 13:**
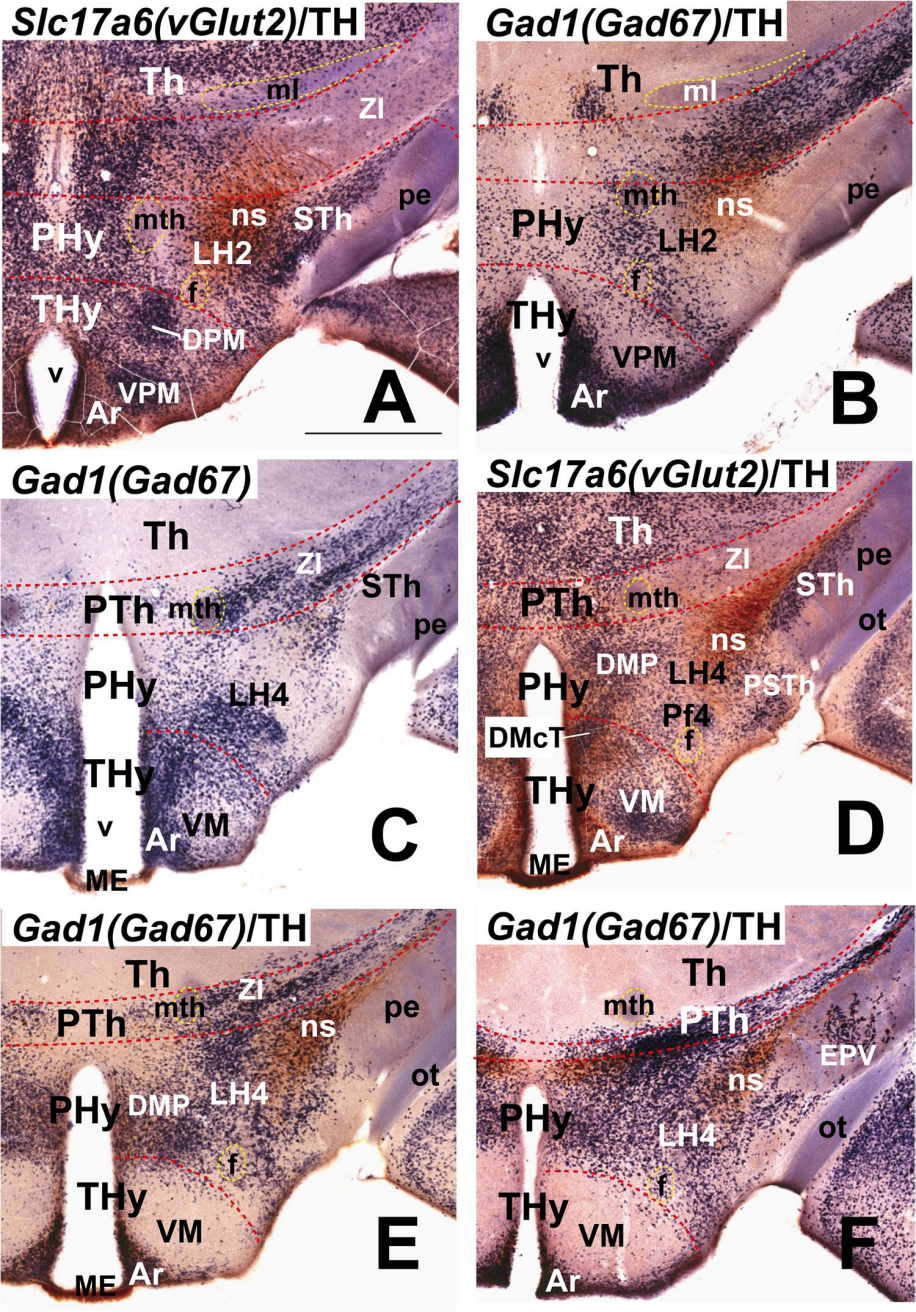
Second panel continuing a series of four, showing the distribution of either *Slc17a6* (*vGlut2*) or *Gad1* (*Gad67*) in adjacent coronal/horizontal sections through an adult mouse hypothalamus, ordered from ventral to dorsal levels. The sections were counterstained with a tyrosine hydroxylase (TH) antibody (brown reaction). Red dash lines indicate the thalamo-prethalamic, prethalamo-hypothalamic, and intrahypothalamic transverse interprosomeric boundaries. Yellow dash lines surround some identified tracts. (**A**, **B**) Coronal sections through the peduncular basal periretromamillary area, containing the lateral hypothalamic subregion 2 (LH2). LH2 cells mostly express *Slc17a6*, a glutamatergic cell marker. (**C–F**) Sections through the basal intermediate retrotuberal area, containing the lateral hypothalamic subregion 4 (LH4). Some LH4 cells in patches express *Gad1* (a GABAergic cell marker; **C**, **E**, **F**) whereas disperse cells express *Slc17a6* (a glutamatergic marker; **D**). Scale bar for all images, indicated in (**A**): 700 µm. For additional abbreviations, see “Abbreviations”

**Fig. 14:**
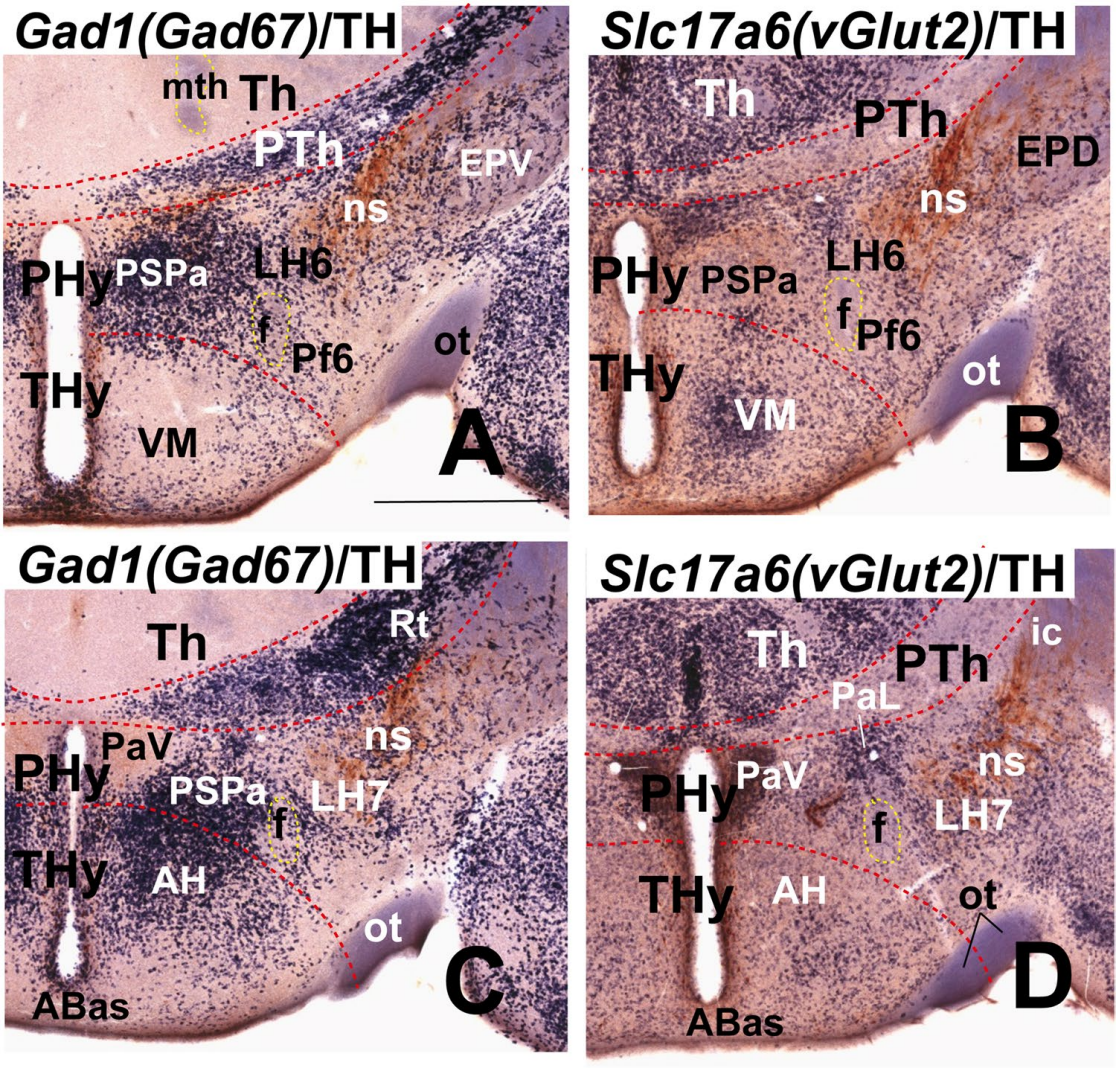
Third panel continuing a series of four, showing the distribution of either *Slc17a6* (*vGlut2*) or *Gad1* (*Gad67*) in adjacent coronal/horizontal sections through an adult mouse hypothalamus, ordered from ventral to dorsal. Sections were counterstained with a tyrosine hydroxylase (TH) antibody (brown). Red dash lines indicate the thalamo-prethalamic, prethalamo-hypothalamic, and intrahypothalamic transverse interprosomeric boundaries. Yellow dash lines surround some identified tracts. (**A**, **B**) Coronal sections through the alar subparaventricular area, containing the lateral hypothalamic subregion 6 (LH6). At this level, most cells express *Gad1*, a GABAergic cell marker. (**C**, **D**) Sections through the alar ventral paraventricular subarea, containing the lateral hypothalamic subregion 7 (LH7). Most cells at this level express *Slc17a6*, a glutamatergic cell marker, and are mixed with dispersed *Gad1*-positive GABAergic cells. Scale bar for all images, indicated in (**A**): 900 µm. For additional abbreviations, see “Abbreviations”

**Fig. 15:**
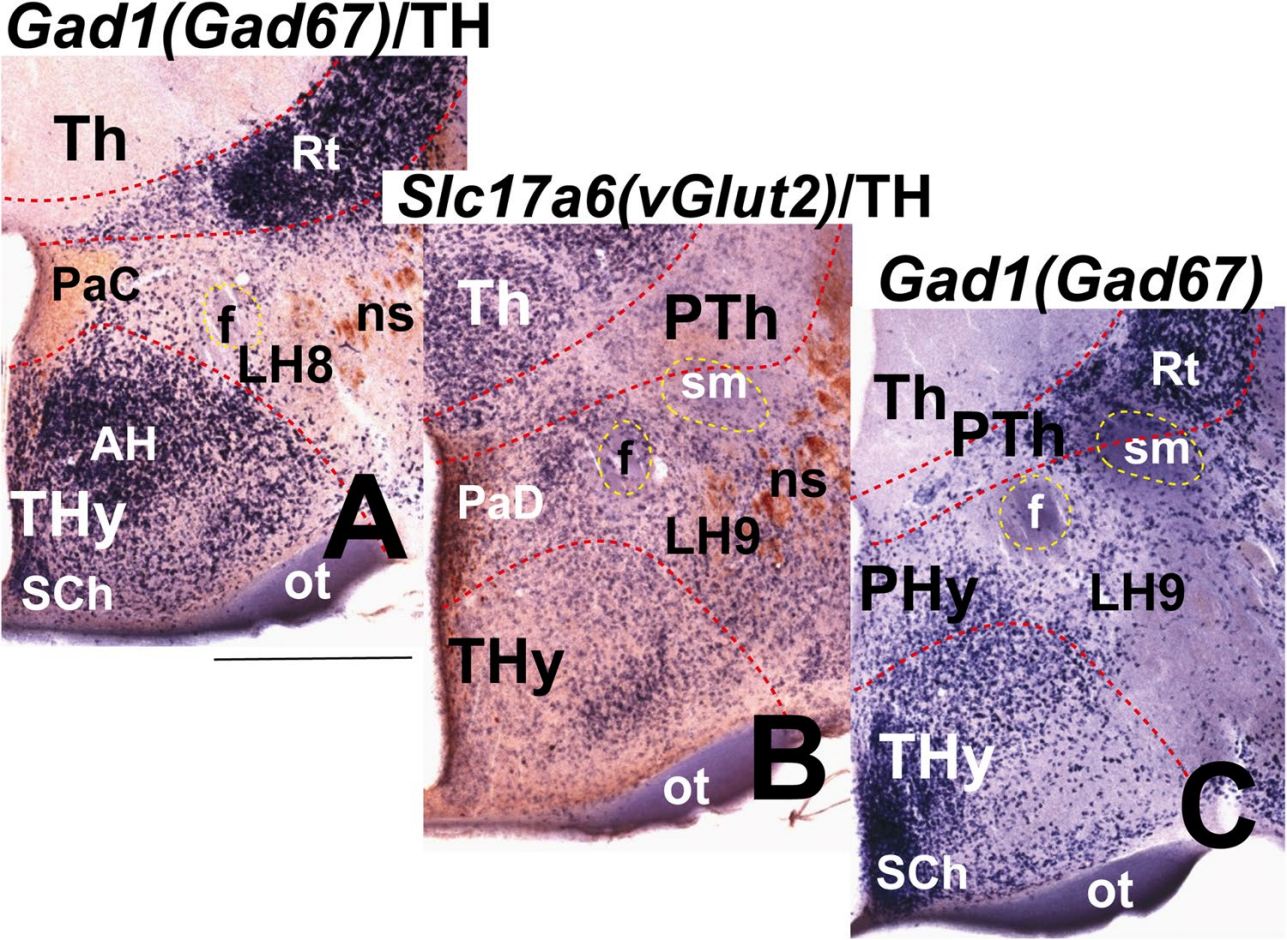
Fourth panel of a series of four, showing the distribution of either *Slc17a6* (*vGlut2*) or *Gad1* (*Gad67*) in adjacent coronal/horizontal sections through the adult mouse hypothalamus, ordered from ventral to dorsal levels. Sections were counterstained with a tyrosine hydroxylase (TH) antibody (brown reaction) except in (**C**). Red dash lines indicate the thalamo-prethalamic, prethalamo-hypothalamic, and intrahypothalamic transverse interprosomeric boundaries. Yellow dash lines surround some identified tracts. (**A**) Section reacted for *Gad1* through the alar central paraventricular area, containing the lateral hypothalamic subregion 8 (LH8); there are very few GABAergic neurons in LH8, consistently with a majority of glutamatergic ones in the whole paraventricular area (see Fig. 8H; see also Puelles et al. [17]). (**B**, **C**) Sections through the alar dorsal paraventricular area, containing the lateral hypothalamic subregion 9 (LH9). Most cells in LH9 express *Slc17a6*, a glutamatergic cell marker, a few GABAergic *Gad1-*expressing cells appearing intermingled. Scale bar for all images, indicated in (**A**): 900 µm. For additional abbreviations, see “Abbreviations”

We analyzed adult lateral hypothalamic GABAergic and glutamatergic populations labeled by *vGlut2* (*Slc17a6*) and *Gad67* (*Gad1*) ISH, respectively, checking their correspondence with the postulated LH sectors. Figures 12–15 show a series of adjacent sections ordered from ventral to dorsal; this material was secondarily immunoreacted for TH. Hypothalamic GABAergic cells are usually related to *Dlx*-expressing progenitor domains whereas glutamatergic cells correlate with *Dlx*-negative domains, as is observable in *Dlx5/6-LacZ* brains (Fig. 8A; see also [102]). GABAergic and glutamatergic cell populations present in adult radial domains in the hypothalamus generally are produced separately (often distinct neighboring domains) though they may intermix partially via tangential migration. We assume that the major cell population is locally originated whereas the minor cell population is migrated [17].

In the basal hypothalamus, LH1- and LH2-cells associated respectively with the RM and PRM subdomains express massively *Slc17a6* (*vGlut2*) transcripts and show few *Gad1* (*Gad67*)-positive GABAergic cells (Figs. 12A–D and 13A, B); the STh and VPM nuclei migrated out of the retromamillary area also are predominantly *Slc17a6* (*vGlut2*) positive (Fig. 13A–D). LH3 cells are largely histaminergic (expressing *Hdc* and other selective markers, as shown in Fig. 5). In contrast, the basal LH4 population found lateral to the DM-P nucleus contains both GABAergic cells, expressing *Gad1* (*Gad67*; Fig. 13C, E, F) and glutamatergic cells, expressing *Slc17a6* (Fig. 13D). The same pattern characterizes the DM nucleus, where glutamatergic cells predominate in its dense core portion and GABA cells mainly populate the surrounding shell area; the glutamatergic cells are thought to be migrated (see comments and additional data in Puelles et al. [17]. Finally, most of the dorsal retrotuberal LH5 cells are glutamatergic with only sparse GABAergic cells intermixed (not shown; see [17]).

In the alar hypothalamus, LH6 cells corresponding to the subparaventricular subdomain are mostly GABAergic *Gad1*/*Gad67*-positive cells, showing scattered *Slc17a6*/*vGlut2* cells (Fig. 14A, B). The alar LH7–LH9 subdomains, corresponding to the ventral, central, and dorsal subdivisions of the Pa domain, display instead mainly *vGlut2* (*Slc17a6*)-expressing cells (Fig. 14C, D and Fig. 15A–C) with scarce *Gad1*/*Gad67-*positive cells. This pattern contrasts sharply with the massively GABAergic substantia innominata subpallial domain found above the Pa complex (not shown).

**Fig. 16:**
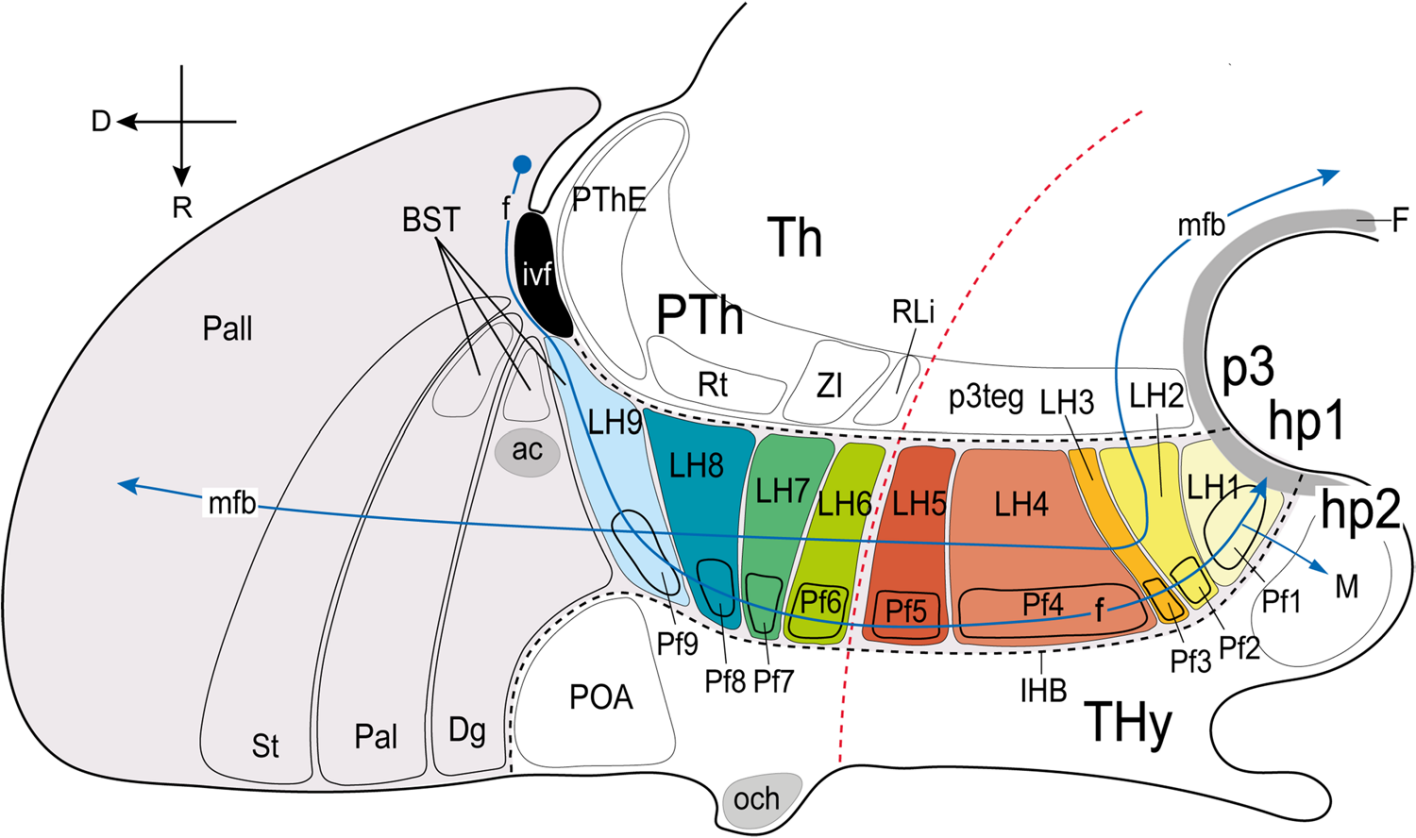
Color-coded prosomeric schema summarizing the postulated ventrodorsal subdivisions of the mouse lateral hypothalamus (LH) in five basal LH subregions (LH1-LH5) and four alar LH subregions (LH6–LH9), and their topographic correlations with neighboring terminal hypothalamic, diencephalic prethalamic, and telencephalic structures. The LH is traversed dorsoventrally by ascending and descending fibers of the medial forebrain bundle (mfb; blue line), and the fornix tract (f: blue line). A ventrodorsal series of perifornical cell subgroups (Pf1-9) is associated with the dorsoventral fornix course, mostly lying next to the intrahypothalamic interprosomeric border (IHB); they are each considered a component of the corresponding LH1-9 subregions. The basal LH1–LH5 sectors limit caudalwards with the nigral tegmentum of prosomere 3 (p3teg); the alar LH6–LH9 subregions contact caudally various prethalamic entities (identified in the schema). The dash black lines identify the prethalamo-hypothalamic and intrahypothalamic transverse interprosomeric boundaries. The red dash line indicates the alar/basal limit. The floorplate (F) is drawn in light gray. The rostral (R) and dorsal (D) spatial directions are indicated in the upper left corner

We summarize in Fig. 16 our schema of ventrodorsal subdivisions of the LH in five basal (LH1–LH5) and four alar (LH6–LH9) cell subregions. All these molecularly distinct subregions are interstitial to the course of the mfb and typically contain at most levels perifornical cell subpopulations associated with the peduncular course of the fornix tract, close to and parallel to the intrahypothalamic boundary.

## Discussion

Prosomeric analysis of 59 markers selected from the ISH database of the Allen Mouse Brain Atlas [103] and the Allen Developing Mouse Brain Atlas [96] leads us to propose a ventrodorsal regionalization of the lateral hypothalamus (LH) according to molecular criteria. This investigation arose from previous examination of dorsoventral divisions of the hypothalamic alar and basal plates in terms of molecularly differentially defined progenitor domains [17, 74, 84, 90, 104]. The fact that the conventional lateral hypothalamus (i.e., as conceived by Nieuwenhuys et al. [18] or Swanson [19]) stretches from dorsal to ventral (in prosomeric terms) orthogonally to a series of longitudinal progenitor domains [17] already suggested that it probably was molecularly heterogeneous and, accordingly, was being treated simplistically as a unit in the literature. Swanson [105] followed the columnar approach and visualized the LH as a longitudinal hypothalamic structure; he thus distinguished a number of anteroposterior (AP) parts of LH neighboring diverse nuclear landmarks; his AP divisions of LH apparently coincide partly with our DV components (see below). Note columnar theory lacks a developmental causal explanation for AP subdivisions within a longitudinal column, so that Swanson’s [105] concept of LH cannot be explained developmentally.

Among the markers used in the present study, chosen empirically for their partial labeling of the LH (see Table 2), there are neuropeptides and neurotransmitters, as well as transcription factors, synaptic proteins, vesicular transporters, and receptors (Table 1). The transmitters are not very selective, except perhaps histamine and dopamine. Fifteen glutamatergic and 15 GABAergic otherwise different neuronal cell clusters were identified in a recent single-cell RNAseq transcriptomic classification of LH cell populations in postnatal mouse brains; each cluster was distinguished by differential expression of transcription factors and neuropeptides, among other markers [42]. However, the cited analysis was focused exclusively on what we regard as basal LH subpopulations, described columnarly as “caudal to the retrochiasmatic area and rostral to the tuberomammillary nucleus in the posterior hypothalamus.” A regional differential location of the identified cell clusters was not investigated. Since several of those cell markers were included in the present study, we will try to correlate both sets of data. Another scRNA-seq analysis of the same group was centered on the mamillary/retromamillary region, also relevant for our approach [87]. Hypothalamic single-cell RNAseq transcriptomic data were reported likewise by Kim et al. [46], who included in their Supplementary data (their Supplementary Fig. 10) an analysis of the LH in E11 to E16 embryonic mice; they identified 11 glutamatergic and 2 GABAergic cell clusters. The analogous study of Chen et al. [43] did not mention LH cell populations, although some of the cell clusters identified may match with LH cell subpopulations found in the present work.

Our detailed prosomeric genoarchitectonic analysis thus reexamines cytoarchitectonic studies of LH cell populations associated topographically with the medial forebrain bundle [18, 23, 24] and expands a tentative genoarchitectonic dorsoventral LH subdivision proposed initially by Puelles et al. [17]. These authors argued that, as classically defined, the LH belongs strictly to the peduncular hypothalamus, understood by them to derive from the hp1 hypothalamic prosomere, which expands dorsally into the evaginated telencephalon (Fig. 16). The related transverse *intrahypothalamic* boundary limiting hp1 from hp2 (or peduncular from terminal hypothalamus territories; PHy/THy) passes roughly in front of the fornix tract, thus including a dorsoventral series of perifornical cell groups within the LH (Figs. 1 and 16). Tridimensional morphogenetic deformation of the PHy as marked by radial glia causes its superficial part to bend at basal levels around the VM mass to reach the lateral tuberal surface rostrally to the peduncle ([17]; their Fig. 8.12). Moreover, Puelles et al. [17] also updated the LH concept by distinguishing the LH, understood as an intermediate stratum within PHy, from a covering *superficial* stratum occupied by the peduncle (lateral forebrain bundle) and associated superficial cell groups such as the STh, parasubthalamic, tuberal, and entopeduncular nuclei. Superficial populations lying rostral to the peduncle, such as the lateral tuberal and parasubthalamic nuclei, which were included by Geeraedts et al. [24] as LH cell populations, are thus ascribed by us to the superficial hypothalamic stratum and not included in our study of LH. Correspondence of cytoarchitectonic LH subdivisions [23, 24] with genoarchitectonic LH subregions (present results) should be regarded as merely tentative.

### Phenotype of LH Basal Subpopulations

Previously, Puelles et al. [17] subdivided the basal LH in a ventrobasal LH subdomain containing *Cartpt-* and *Gal-*positive GABAergic cells and a dorsobasal LH subdomain with *Hcrt-*expressing glutamatergic neurons; periretromamillary and retromamillary LH subpopulations presently introduced by us were not contemplated. According to our present schema the basal LH is divided in five molecularly distinguishable subregions—LH1–LH5—superposed in ventrodorsal order one upon another. These are associated causally with the fundamental ventrodorsal molecular regionalization of the basal peduncular hypothalamus (basal PHy) in retromamillary, periretromamillary, and ventral, intermediate, and dorsal retrotuberal progenitor subdomains (RM, PRM, RTuV, RTuI, RTuD; [17]), representing in each case the respective LH intermediate stratum. Though it may be unclear how far the RM and PRM domains are traversed by the medial forebrain bundle (the latter is usually taken as an auxiliary criterium to define the LH, and we believe it traverses at least the PRM), we regard the structural and developmental (outside-in neurogenetic layering) classification into periventricular, medial, lateral, and superficial strata strictly applicable to these ventralmost DV tiers. The RM, PRM, and RTuD subdomains contain predominantly glutamatergic cells, as observed also in LH1, LH2, and LH5, respectively, while RTuI shows mainly GABAergic cells (as does LH4), and RTuV displays histaminergic neurons within a singular molecular profile (like LH3) ([17]; present results; Table 1). LH5 cells are distinctly characterized by *Hcrt*-expression and clearly correspond to the (now obsolete) dorsobasal LH subdomain proposed by Puelles et al. [17], whereas LH4-LH3 jointly correspond to the (likewise obsolete) old ventrobasal LH subdomain. We think that primary ventrodorsal basal plate regionalization within PHy and a diversity of local LH cell subpopulations originated differentially in each DV basal subregion is probably generated by ventralizing SHH gradiental effects stemming from the floorplate (F in Fig. 16) and the notochord and by secondarily added WNT8 or other effects released from the likewise longitudinal circumventricular ventricular hypothalamic organ differentiated at RTuV level (HVO; [17, 73, 74]). Antagonistic dorsalizing BMP/WNT effects spreading from the roof plate are possibly involved as well at initial stages, but may be weaker afterwards, due to the interposed mass of the telencephalon (see [74]).

With respect to cytoarchitectonic LH subdivisions proposed in Geeraedts et al. [24] their lateral supramamillary nucleus (our RML) clearly matches our LH1, whereas the sum of our LH2-LH3 may correspond to their part 3 and caudal part 4 of the lateral hypothalamic nucleus. In their turn, our LH4-LH5 corresponds to their part 2 and rostral part 4 of the lateral hypothalamic nucleus.

LH1 corresponds to the lateral retromamillary nucleus or RML (classical lateral “supramamillary” nucleus of columnar accounts; [19, 24]); Paxinos and Watson [106] and Paxinos and Franklin [107] already use the prosomeric “retromamillary” terminology in their Rat and Mouse Brain Atlases. Indeed, LH1/RML is part of the RM hypothalamic subdomain, found topologically *caudal* to the mamillary subdomain along the cephalic flexure (Fig. 16) [17, 106, 107]. Radially, LH1/RML is located between the medial retromamillary and the superficial retromamillary nuclei [17], thus agreeing with the stratigraphic LH concept.

We found expression of at least twenty genes in LH1 cell subpopulations, eight of which are selective of this LH subregion (see Table 2). Most LH1 cells are glutamatergic and express *Slc17a6* (also named *vGlut2*; [17]; present results); this gene encodes a vesicular glutamate transporter. LH1 may correlate with cluster 4 of Mickelsen et al. [42] that contained *Tac1-* and *Pitx2*-expressing glutamatergic cells. *Tac1* encodes a precursor protein that produces the peptides substance P and neurokinin A of the tachykinin family, whereas *Pitx2* encodes a paired homeodomain transcription factor (PITX2). Navarro et al. [108] illustrated mouse *Tac1* positive cells in what we regard as the LH1/RML locus (not identified by the authors; see their Fig. 4). *Pitx2* expression was detected in rat RML cells (identified as supramamillary) and in migrated subthalamic nucleus cells ([109]; their Fig. 3). This migration is severely altered in *Pitx2* null mice mutants [80]. It is not yet understood what causes some RM cells to migrate tangentially away in different directions while others stay in place (see [82] on the ventral premamillary nucleus).

According to present results, LH2 cells may be discriminated by the expression of five markers (*Dlk1*, *Irs4*, *Otp*, *Peg10*, and *Penk*) which are not expressed in either LH1 or LH3 cells (Table 2). LH2 cell subpopulations are restricted to the dorsoventral level of the periretromamillary subdomain (PRM; [17]) within basal PHy, whose cells were previously characterized by massive *Sim1*/*Otp* expression from early embryonic stages onwards ([17, 82, 84]; present results). In columnar accounts, the PRM area was included in the classical “posterior hypothalamus” (see [86]; his Figs. 16–18, reproduced in [17]; their Fig. 8.3). However, the columnar “posterior hypothalamus” included also a strictly non-hypothalamic *diencephalic tegmental* part that reaches the retroflex tract in most accounts (see historical review of this concept in [17]). Expression of *Otp* and *Sim1* dissociates cleanly the hypothalamic and diencephalic parts of the old “posterior hypothalamus.’ Both *Otp* and *Sim1* genes have an essential role in the differentiation of hypothalamic neuroendocrine glutamatergic subpopulations such an the paraventricular, supraoptic, and arcuate nuclei [110–115], but their function in the differentiation of LH2 cells has not been analyzed. In the adult rat brain *Irs4*-expressing cells were illustrated at what we identify as the LH2 locus ([116]; their Fig. 2F).

The glutamatergic cell clusters 8 (with *Otp*/*Cbln2*/*Ebf3*-positive cells) and 11 (with *Otp*/*Mdga1-*positive cells) of Mickelsen et al. [42] may also correspond topographically with LH2. All the additional markers cited of these phenotypes that co-express *Otp* are present in LH2 cells (present results; some of them also appear within LH1, but devoid of *Otp* co-expression). *Ebf3* and *Cbln2* cells were previously illustrated during mouse development in hypothalamic retromamillary and periretromamillary subdomains [117, 118]. Moreover, Litwack et al. [119] cited the presence of *Mdga1-*positive cells in the adult rat hypothalamus (without showing them). The *Ebf3* gene encodes the “early B cell factor 3,” a member of the EBF transcription factor family, which has a role in neuronal differentiation and maturation [117]; the *Mdga1* gene encodes a glycosylphosphatidylinositol (GPI)-anchored membrane protein, a cell surface glycoprotein; and the *Cbln2* gen encodes a cerebellin 2 precursor protein implicated, as other cerebellins, in the formation and/or function of synapses [120–125].

Production of histaminergic *Hdc*-expressing neurons characterizes the thin LH3 sector, which is part of the ventral retrotuberal subdomain (RTuV), as defined by Puelles et al. [17]. This cell type is also produced in the continuous ventral tuberal subdomain of THy (TuV), and both coincide with the hypothalamic ventricular organ (HVO), a potential secondary organizer [17, 73, 74]. In recent single-cell transcriptomic classifications of hypothalamic populations, a separate histaminergic cell cluster was identified, distinct from glutamatergic and GABAergic cell clusters [43, 87]. Consistently, the LH3 cells do not express either vesicular glutamate (vGLTU2) or GABA transporters (*Slc17a6* and *Slc32a1* genes; [17, 43]; present results). *Prph* and *Wif1* are selectively co-expressed in *Hdc* cells ([43]; their Fig. S5C; [87]; their Fig. 5). *Prph* encodes peripherin, a type-III intermediate filament. Erickson et al. [88] showed *Prph* expression in mouse histaminergic neurons. Hu et al. [126] detected in the postnatal hypothalamus *Wif1* signal (encoding the WNT inhibitory factor 1; see also [127]) but did not show its topography. These coincident findings may imply that the thin LH3 subdomain produces a single cell type, though, of course, many more markers would need to be examined to fully establish this point. Note that the unlabeled cells seen intermixed with the labeled ones may derive by dispersion from molecularly different neighboring LH subdomains.

The larger LH4 subdomain is a component of the intermediate RTu subdomain (RTuI), characterized medially by the peduncular dorsomedial nucleus, which displays a glutamatergic dense core surrounded by a dispersed shell population of GABAergic cells (present results; [17]; the latter authors deduced that the GABAergic neurons of DM are endogenously produced, whereas the glutamatergic ones in the core possibly are migrated tangentially from a different source). There is also in THy a continuous terminal DM nucleus portion with analogous core/shell structure. LH4 cells indeed express markers related to the GABAergic phenotype such as *Slc32a1*, *Gad1*, and *Gad2*. Moreover, they co-express the genes *Cartpt*, *Gal*, *Nts*, and *Tac2* encoding respectively the neuropeptides cocaine- and amphetamine-regulated transcript, galanin, neurotensin, and tachykinin [52, 53]. *Dlk1* is also expressed in LH4 cells; this gene encodes the delta-like non-canonical notch ligand 1, a protein implicated in cell proliferation or differentiation depending on the local context, which is also involved in hormone-producing pituitary cells [128–134]. These cell phenotypes match with two GABAergic clusters (1 and 3) identified by Mickelsen et al. [42]; cluster 3 is characterized by co-expression of *Cartpt* and *Nts*, and cluster 1 contains cells expressing *Nts*, *Gal*, and *Dlk1* transcripts ([42]; their Fig. 6). Other markers found in both our LH4 cells and cell cluster 3 are *Gpr101*, *Nek7*, *Pdyn*, and *Penk* ([42]; their Fig. 6).

An important subpopulation of *Cartpt* neurons localize in LH4 ([135, 136]; present results). In the rat, 67% *Cartpt/*CART neurons co-express *Gad67* mRNA [136]. Moreover, anorexigenic galanin (Gal) neurons colocalize with *Cartpt* and *neurotensin* (*Nts*) transcripts in the lateral hypothalamus (Gal/*Cartpt* neurons and Gal/*Nts* neurons), including some perifornical subpopulations [137]. It is of interest that galanin neurons do not colocalize with hypocretin (HCRT) cells nor with melanin-concentrating hormone (MCH; [137]), two markers expressed specifically in the overlying LH5 cells.

*Trh*-positive cells appeared likewise associated with LH4 (present results). Mickelsen et al. [42] found two glutamatergic *Trh*-expressing clusters; one of them also contains *Cbln2* signal, a marker also found in LH4 cells (present results; Table 2); both cell clusters co-express *Otp* a characteristic marker of the paraventricular (Pa) and periretromamillary domains [17, 84, 111, 138]. According to these findings, *Trh*-positive glutamatergic cells in LH4 may have migrated ventralward from the *Otp*-positive peduncular PaV progenitor domain, as was previously suggested [104]. PaV contains mostly locally originated glutamatergic cells ([17]; present results).

*Hcrt*- and *Pmch*-expressing neurons characterize LH5, a component of the dorsal retrotuberal subdomain (RTuD), the dorsalmost part of the basal plate. Swanson et al. [139] also observed these cell types in the adult rat brain, describing them somewhat less precisely within their columnar paradigm as located in the “caudal part” of LH (roughly equivalent to our basal territory; note the basal notion allows a causal explanation). *Hcrt* and *Pmch* cells are distinct intermixed populations within LH5 [140]. 50% of rat hypocretin (HCRT) neurons were found to be perifornical [75]. Our data suggest that while both *Hcrt and Pmch* cell populations seem to have the same anatomic origin within the RTuD area, where they tend to distribute at least within the medial and lateral strata (note the medial stratum is shared also with the A13 dopaminergic neurons; [17, 89]), the *Pmch* cells secondarily spread out more than the *Hcrt* cells, invading parts of the subjacent RTuI area (DM-P area) and even the caudally neighboring prethalamic zona incerta (see also [141]). The *Hcrt and Pmch* genes encode hipocretin/orexin and pro-melanin-concentrating hormone, respectively; both peptides are implicated in arousal, feeding, and energy metabolism [9, 27, 28, 142–149]. Mouse *Pmch* and *Hcrt* subpopulations have been ratified as two molecularly distinct glutamatergic clusters by single-cell transcriptomic analysis [42]. Consistently with such data, the LH5 consists largely of *Slc17a6*-positive glutamatergic cells ([17, 141]; present results).

The cluster containing the large-sized *Hcrt-*expressing glutamatergic neurons co-expresses, among other markers, *Pdyn*, and *Nek7* [42]; these two phenotypes are also found in LH5 (present results; see Table 2). Interestingly, the *Lhx9* gene which encodes a transcription factor member of the LIM homeodomain family is co-expressed in all *Hcrt*-positive cells, whereas the transcription factor NKX2.1 is not expressed at all in mantle *Hcrt* cells [150]. Indeed, *Lhx9* is expressed early on in the *Nkx2.1*-negative mantle layer of the RTuD subregion lying just ventral to the *Lhx6*/*Nkx2.2*-positive alar liminar band [17, 36]. The LHX9 transcription factor has been implicated in the specification of hypocretin cells in mice [36, 151, 152] and zebrafish [153, 154]. Removal of this gene resulted in a 30% decrease of *Hcrt* neurons in the mouse [151]. *Hcrt* cells also co-express *Nptx2* (99%), and *Nek7* (84%) transcripts [42, 150], both also observed by us in LH5 cells. *Nptx2* encodes neuronal pentraxin-2 and *Nek7* (NIMA) encodes the *never in mitosis gene A*, a member of the serine/threonine kinases family.

*Pmch* neurons produce melanin-concentrating hormone (MCH). They are mostly distinct from *Hcrt* cells though they coincide within LH5 and the local medial stratum (our posterobasal nucleus combined with the A13 dopaminergic cell group). *Pmch* neurons possibly emerge within the *Nkx2.2*-positive and *Lhx9*-negative *subliminar subdomain* of RTuD in both rats and mice [17]; the underlying *Nkx2.2*-negative “main subdomain” of RTuD is where *Lhx9* signal is observed, coinciding with the mantle locus where *Nkx2.1* signal is absent. Croizier et al. [155, 156] referred to this location as the “intrahypothalamic diagonal” of Shimogori et al. [36], without noticing that this origin of *Pmch* neurons is largely restricted therein to what we call peduncular hypothalamus, a partition not recognized in their hypothalamic schema. The combined longitudinal TuD/RTuD domain of the prosomeric model was wrongly interpreted as “diagonal” because Shimogori et al. [36] naively combined the orthogonal columnar and prosomeric length axes, thus obtaining arbitrarily an extra diagonal direction in morphospace ([17]; see footnote in p.246).

Differently than the *Hcrt* cells, *Pmch*-positive glutamatergic neurons, identified as cluster 1 by Mickelsen et al. [42], co-express *Nkx2.1* in the rat embryo [155]. *Nkx2.1* signal was also found in 60% of MCH neurons in the adult mouse [150]. We thus propose that *Pmch*/MCH cells originate from the *Nkx2.1*-positive subliminar RTuD progenitor subdomain at early embryonic stages, while *Hcrt* neurons, known to be *Nkx2.1*-negative, probably arise within the underlying *Lhx9*-positive “main” RTuD progenitor subdomain, whose mantle later is devoid of *Nkx2.1* signal (not so the ventricular zone). Consistently with our conclusion, *Pmch/MCH* cells do not express *Lhx9* [155], a transcription factor related instead to *Hcrt* cell differentiation [36, 151–154]*.* Thus, NKX2.1 seems to be continuously transcribed during MCH cell differentiation, as a characteristic of the subliminar RTuD domain, while LHX9 signal, which is expressed selectively within the underlying main RTuD subdomain in combination with downregulation of *Nkx2.1* in postmitotic neurons, is clearly related to local HCRT cell differentiation. The apparent ventral displacement of some *Pmch* neurons beyond LH5 into LH4 may reflect the tendency of neurons derived from the RTuD band to migrate ventralwards (discussed in [17], pp.283–286).

The general expression of *Nkx2*.1 in the basal hypothalamus reportedly depends on early activation of GLI1 downstream of the SHH signal [94, 157]. Inhibition of the SHH pathway by cyclopamine in E11 mice produces a dramatical reduction of *Pmch* mRNA level and no *Pmch/*MCH signal was detected in embryonic mutant mice lacking *Shh* [155].

A subpopulation of *Cartpt* cells was found within the same LH5 subregion (present results). *Cartpt*/CART is also co-expressed in a subpopulation of *Pmch*/MCH neurons ([136, 141, 158, 159]; reviewed in [135]) that specifically project to the cerebral cortex [160]. 66% of MCH neurons co-express the *Cartpt* transcript in the mouse [150] and 70% in the rat [136]. Both *Cartpt* and *Pmch* cells were found in LH5 and LH4 in correlation with either GABAergic or glutamatergic phenotypes. Approximately 67% CART-expressing neurons express *Gad67* [136]; we estimate that this subtype probably appears mostly located within the LH4 subregion, rather than in LH5.

Interestingly, the *Dlk1* gene is likewise expressed in LH5 cells (present results), as well as in the related neuroepithelium; it codifies for DLK1, a protein expressed in virtually all hypocretin- (HCRT- or orexin-) and dynorphin-positive (DYN) neurons but not in MCH- or CART neurons in the rat LH [161]. This suggests again a dissociation in the cellular phenotypes of neurons born at the subliminar versus main RTuD subdomains. DLK1 promotes neurogenesis in progenitors by modulating Notch and BMP signaling [162, 163] and may thus be implicated in generating hypocretin/dynorphin cells. Furthermore, a small group of DLK1-positive neurons also express galanin (GAL) and neurotensin (NTS), though galanin is not expressed in hypocretin and MCH neurons [137].

According to present results, *Igf1*, which encodes the insulin-like growth factor 1 (IGF1), is also expressed selectively in many LH5 cells; all *Pmch*-expressing neurons contains also *Igf1* [141]. IGF1 is an essential growth factor involved in different neural processes including neurogenesis. *Igf1* overexpression produces an increase in brain size [164] and *Igf1* lack of function originates a reduction in brain size in the mouse [165]. Moreover, IGF1 is involved in proliferation and migration of GABAergic and glutamatergic neurons as well as in synaptogenesis [166, 167]. In the adult brain, IGF1 is also implicated functionally in the somatotropic axis, acting downstream of the growth hormone, as well as in hippocampal neurogenesis, neuronal circuit maturation, and cell neuroplasticity (see reviews in [168, 169]).

Wang et at [141] recently subdivided the tuberal LH (including perifornical populations) in nine spatially and molecularly distinct subregions analyzing markers from single-cell RNA sequencing transcriptomic (scRNA-sq) datasets by Expansion-Assisted Iterative Fluorescence In Situ Hybridization (EASI-FISH) in 300-µm-thick brain sections of the adult male mouse. For scRNA-sq analysis, they used transgenic male mice (*Agrp-IRES-Cre* x *Ai9*) and the authors identified the presumed boundaries of LH as the territory targeted by AGRP-positive axon terminals. The fact that they only studied the tuberal LH suggests that this criterium leaves out the alar parts of LH and is therefore imperfect. They found 17 glutamatergic (*Slc17a6*-expressing) and 17 GABAergic (*Sclc32a1*-expressing) cell clusters. Based on the expression of *Meis2*, *Otp*, *Slc17a6*, and *Sclc32a1* markers in thick sections, Wang et al. [141] proposed nine subregions in the lateral hypothalamic area (referring actually mainly to its tuberal/retrotuberal part according to our model) arranged in the mediolateral and dorsoventral dimensions of the columnar model (note their dorsoventral dimension in coronal sections corresponds to our prosomeric *caudorostral* dimension). Three of these subregions were depicted adjacent to the prethalamic incertal alar region and were identified as “dorsal diagonal band,” “hypocretin-positive diagonal band,” and “dorsolateral diagonal” LH parts. Another LH region (subdivided into lateral and medial subregions) was named “suprafornical diagonal band.” The descriptor “diagonal band” refers to the “intrahypothalamic diagonal” of Shimogori et al. [36], which corresponds to our alar *longitudinal* subparaventricular area [17]. However, we think that all these areas called “diagonal band”, and particularly the hypocretin-positive one, lie within the basal hypothalamus, at the dorsal retrotuberal region (see our Fig. 7A), and are thus wrongly referred to the “intrahypothalamic diagonal.” In addition, several mediolaterally disposed perifornical subregions -also in the basal hypothalamus, in our opinion—were distinguished, identified as “medial” and “lateral” fornical regions (the latter subdivided into medioventral, dorsolateral, and ventrolateral “lateral” parts). The “dorsal diagonal” and the three “lateral” fornical subregions contain mainly excitatory neurons (*Slc17a6* + /*Otp* +), as does the “dorsolateral diagonal” subregion (*Slc17a6* + /*Meis2* +), whereas inhibitory cells largely occupy the medial and lateral “suprafornical diagonal” subregions whose cells are *Slc32a1* + /*Meis2* + . The “medial fornical” subregion also shows inhibitory cells expressing *Slc32a1* + /*Otp* + . We understand the LH region analyzed by Wang et al. [141] as corresponding topographically in part to our LH5 domain (mainly containing glutamatergic cells) and in part to our LH4 domain (containing a mixture of glutamatergic and GABAergic cells) sectors. Both lie within the peduncular basal hypothalamic LH region which is classically referred to as “tuberal area,” though the prosomeric model more precisely identifies it within the hp1 prosomere and PHy as “retrotuberal area.” Indeed, the latter is restricted to the peduncular hypothalamus whereas “tuberal area” is a descriptor of the corresponding terminal hypothalamic basal domain, where no LH exists (Fig. 16). The coronal section illustrated in their Fig. 3A [141] largely corresponds to our Figs. 13E, F. This part of the basal LH possibly relates also to the lateral and medial “middle LH groups” described by Swanson [105] as juxtadorsomedial, perifornical, (supra- and subfornical), and lateral parvicellular LH subdivisions (see also Swanson et al. [170]; his “supporting information 9 and 10”); other LH subdivisions of Swanson, such as diverse “juxtaventromedial parts,” lie topographically within our terminal hypothalamus (THy), which according to us does not contain any lateral hypothalamic populations (and contains no part of the medial forebrain bundle). The “hypocretin-positive diagonal” subregion of Wang et al. [141] relates clearly to our LH5, since it typically contains a glutamatergic *Hcrt*-positive subpopulation whose neurons receive AGRP afferences from the arcuate nucleus [140, 171], jointly with a distinct *Pmch*-positive peptidergic subpopulation also detected by Wang et al. [141]. A high percentage of *Pmch*-expressing neurons are *Cartpt*-positive (77% according to Wang et al. [141]), a subpopulation which is also mainly related to our LH5 (present results). However, other peptidergic populations such as *Nts*- and *Trh*-expressing cells, and a group of *Cartpt*-positive cells, were associated by us with LH4. Wang et al. [141] reported *Trh*-positive neurons and *Otp*-positive cells in their fornical subregions. However, the presence of *Otp/Trh*-expressing neurons in the retrotuberal LH region may be due to tangential migrations reported from alar and/or basal *Otp-*positive progenitor domains [37, 84], i.e., the alar paraventricular area and/or the basal perimamillary/retromamillary domain, both of which produce glutamatergic cells (see e.g. [138] and our Fig. 4A). According to Wang et al. [141], in their “*Hcrt*-positive diagonal” territory there appear *Nts*-positive neurons co-expressing *Grp101* and *Gal* signals; both markers were detected in cell populations of the LH5 sector in our present work (see Table 2). On the whole, the Wang et al. [141] analysis was handicapped in its anatomical approach by an incomplete and possibly outdated model of the LH region. Transcriptomic studies are obviously doubly sensitive to this sort of problem since it affects both the dissection of material and the interpretation of results.

In summary, we subdivided the basal LH into five regions (see Fig. 16), three containing glutamatergic cells (LH1, LH2, LH5), a fourth including mostly GABAergic cells (LH4), and the fifth containing essentially only histaminergic cells (LH3). Each of these LH subregions is characterized by unique combinations of transcription factors, cell adhesive molecules, neuropeptides, and receptors, among other markers. Whereas our topographic analysis of cellular heterogeneity in the basal LH in correlation with postulated developmental units (basal dorsoventral progenitor domains of Puelles et al. [17]) led us to the LH1-LH5 compartments, the single-cell transcriptomic LH study of Mickelsen et al. [42] distinguished 15 glutamatergic and 15 GABAergic basal neuronal clusters (17 in the study of Wang et al. [141], referring mainly to our LH4 and LH5 domains). This implies that, as discussed amply in the case of LH5, several cell clusters apparently may coexist intermixed within our developmental units (with the possible exception of the simpler histaminergic LH3 unit). A salt-and-pepper generative pattern may need to be considered in some of these cases, since a temporal sequence is not likely given the equivalent spatial stratification of the different intermixed LH cells (overall outside-in neurogenetic pattern). With respect to glutamatergic cell clusters, the *Tac1-* and *Pitx2*-positive cell cluster 4 of Mickelsen et al. [42] may be a migrated subpopulation of LH1, the *Otp*/*Cbln2*/*Ebf3-*positive cell cluster 8 and *Otp*/*Mdga1*-positive cell cluster 11 may be subpopulations of LH2, and the *Pmch/Nkx2.1/Nkx2.2/Carpt-*expressing cell cluster 1 (MCH cluster) and the *Hcrt/Dlk1/Lhx9/Nptx2/Nek7/Plagl1/Pdyn*-positive cell cluster 5 (hypocretin cluster) are subpopulations of LH5. In this case, we discussed data suggesting that the related progenitor domain (RTuD) probably may be subdivided into a thin dorsal subliminar *Nkx2.2*-positive band (the source of *Pmch* cells) and an underlying main subdomain that lacks this signal and combines selectively *Lhx9* expression (origin of *Hcrt* cells). As regards GABAergic cell clusters, the cell clusters 1 (*Nts*/*Gal*/*Dlk1*-expressing) and 3 (*Carpt*/*Nts*/*Gpr101*/*Nek7/Pdyn*/*Penk-*expressing) of Mickelsen et al. [42] may represent distinct LH4 subpopulations. A histaminergic cell cluster (*Hdc/Prph*/*Wif1-*expressing) described by Chen et al. [43] matches the LH3 unit (but note that variously displaced *Hdc* cells appear at all radial levels of RTuV and TuV domains (and even within RM and M), this population thus not being totally restricted to LH [17]).

### Phenotype of LH Alar Subpopulations

Puelles et al. [17] tentatively subdivided the mouse alar LH in three dorsoventral domains named according to the estimated prethalamic neighbors as pre-incertal LH (with *Dlx5/6*- and *Nkx2.2/Lhx6*-positive GABAergic cells), pre-reticular LH (with *Tbr1*-expressing glutamatergic cells) and pre-eminential LH (with *Fezf2*- and *Tbr1*-positive glutamatergic cells). Our recent analysis of prethalamic genoarchitecture [40] disclosed an error in one of the cited associations: the incertal prethalamic territory is not continuous rostralward with the *Dlx5/6*- and *Nkx2.2/Lhx6*-positive subparaventricular hypothalamic domain, but with the *Dlx5/6*- and *Nkx2.2/Lhx6*-negative ventral part of the paraventricular domain. This means that all three initially postulated alar LH units are related to the tri-partite paraventricular complex (PaD, PaC, PaV). The underlying and molecularly distinct hypothalamic subparaventricular domain was found to be continuous backwards instead with the rostral liminar band (RLi) defined by us just above the prethalamic alar-basal boundary; the latter is distinguished by early expression of *Nkx2.2* and *Lhx6* jointly with *Dlx5/6* and *Arx* [40]. This correction of the hypothalamus model requires the proposal of a fourth alar LH component associated with the subparaventricular domain. In our present alphanumeric terminology, we thus subdivide the alar LH region into four subregions: LH6-LH9. LH6 is a component of the peduncular subparaventricular area (PSPa) which is generally characterized by *Dlx/Arx*-positive GABAergic cells. The LH7-LH9 units belong respectively to the ventral, central, and dorsal peduncular paraventricular area (PPa), which generally displays *Otp/Sim1*-positive glutamatergic cells ([17, 133]; present results).

Comparison with the cytoarchitectonic LH subdivisions of Geeraedts et al. [23, 24] suggests that their lateral hypothalamic nucleus (part 1), intermediate hypothalamic area (part 1), and dorsal hypothalamic area may correspond to our LH6-LH8, whereas our LH9 sector (included in the TOH domain) may be equated to a subarea of the lateral preoptic region in their account.

As mentioned above, LH6 is a component of the peduncular subparaventricular alar subdomain (PSPa) located rostral to the prethalamic RLi (see [40]). Its cells are mostly *Dlx5/6*-positive GABAergic ([17, 40]; present results), and some also express *Ntm*, *Pnoc*, or *Nkx2.2* mRNAs (present results). *Nkx2.2* cells observed within LH6, together with *Nkx2.2*-positive cells in the ventral and dorsal Pa subnuclei (PaD, PaV), are interpreted as originated in the *Nkx2.2*-positive liminar alar RLi progenitor band found just above the alar-basal boundary; both LH6 and alar RLi display *Dlx5/6* expression ([40]; present results).

Alar *Lhx6* signal appears just dorsal to basal *Lhx9* expression in the embryonic mouse hypothalamus [17, 36], with *Lhx9* signal being restricted to the TuD/RTuD area [17]. *Lhx6* signal accordingly relates with the peduncular and terminal SPa area. *Lhx6* mRNA is known to be expressed in *Cart* (*Cartpt*)-positive neurons at P6 [36]. However, *Cartpt*-expressing cells are not found in alar but in basal LH populations in the adult mouse (present results); a ventralwards migration of SPa-originated *Cartpt*/*Lhx6* cells would explain these findings (see [90]. Recently, Kim et al. [172] found three clusters of postnatal *Lhx6*-eGFP hypothalamic neurons whose cells were GABAergic (expressing GABA transporter *Slc32a1*), and showed either peptidergic or nonpeptidergic markers (*Calb1*, *Calb2*, *Cck*, *Gal*, *Nos1*, *Pnoc*, *Penk*, *Th*, *Trh*; their Fig. 3). These authors did not assess the possibly differential ascription of such neurons to the two hypothalamic sites known to express *Lhx6* [36], namely what the latter authors call “intrahypothalamic diagonal” (i.e., our longitudinal RLi band associated to SPa; [40]) and “tuberomammillary band” (i.e., our RTuV/TuV subdomain; [17]). *Pnoc*-positive cells are restricted to LH6 at E18.5, but the *Pnoc*-positive population is widely dispersed among hypothalamic cells at postnatal mouse stages (present results; [173]; Allen Developing Mouse Brain Atlas). Kim et al. [172] found *Pnoc* signal associated with postnatal *Lhx6*-expressing hypothalamic neurons in one of the cited clusters, which may thus correspond to the RLi/SPa progenitor domain. The *Pnoc* gene encodes the prepronociceptin peptide precursor. Some LH6 cells also express the *Th* transcript, encoding the enzyme tyrosine hydroxylase (TH) that indicates a dopaminergic cell phenotype; *Th* was one of the markers co-expressed in *Lhx6* cells in two of the mentioned clusters ([172]; see [89]).

LH7 is the ventralmost lateral hypothalamus component of the peduncular paraventricular alar subdomain (PPaV). Its cells are *Slc17a6*-positive (a vesicular glutamate transporter) and combine *Otp*/*Sim1* expression. The LH7 subregion contacts caudalwards the GABAergic incertal prethalamic complex ([40]; present results). According to our results, three markers are restricted to LH7 cells, *Trh* (encoding thyrotropin-releasing hormone), *Sst* (encoding somatostatin), and *Mdga1* (encoding MAM domain-containing glycosylphosphatidylinositol anchor 1)*.* A PaV source of *Trh*-expressing cells was previously suggested by Morales-Delgado et al. [104]. Additionally, *Sst*-positive cells were found in an *Otp*-expressing perifornical subpopulation of the PPaV area, next to the intrahypothalamic border ([133]; their Fig. 5B, C, M, N). *Mdga1* expression was not previously reported within the alar LH but was correlated instead with a basal LH cluster [42]. *Nkx2.2-*positive cells held to have migrated dorsalward into the Pa area apparently originate from the RLi band [17] and colonize preferentially the medial and lateral PPaV portions. Some of these cells are found in the LH7 where they were not previously identified. Numerous tangential cell migrations have been reported in the hypothalamus [17, 90, 104, 133, 174, 175], so that a measure of caution is needed when interpreting postnatal and adult data on cells located in postmigratory locations more or less distant from the progenitor areas where they apparently originated.

The overlying LH8 sector displays characteristically in the adult mouse *Meis2-*, *Lhx5*, and *Tbr1-*positive glutamatergic cell subpopulations. The three markers have an early embryonic expression in the prethalamic eminence of the diencephalon, from where cells migrate partly into the paraventricular area and other forebrain areas. There is a progressive increase in the number of superficial and intermediate *Tbr1-*, *Lhx5-*, and *Meis2-*positive cells in the central paraventricular area which contrasts with a decrease of eminential *Meis2*-, *Lhx5*-, and *Tbr1*-positive cells (see Allen Developing Mouse Brain Atlas). Descriptive and fate-mapping results in the chick showed that paraventricular *Tbr1*-expressing cells migrate tangentially rostralwards from the prethalamic eminence [91, 92] whose glutamatergic cells initially express *Tbr1*/TBR1, *Lhx5*, and *Meis2* [40, 93, 176]. Mouse *Tbr1*-, *Lhx5*-, and *Meis2*-positive LH8 cells accordingly may be likewise migrated eminential cells.

LH9 lies within the dorsal part of the PPa area (PPaD) which is characterized by *Sim1*-and *Otp* expression in its mantle layer [17], as is the rest of PPa, but also differentially expresses *Foxg1* [37]. Typically, it displays next to the hypothalamo-diencephalic boundary a dorsalward *Sim1*-positive expansion of PPaD into a curved spike that enters the telencephalon through the floor of the interventricular foramen and follows the bottom of the terminal sulcus to reach the pallial amygdala. This telencephalic extension was recently named the *hypothalamo-amygdalar corridor* (HyA; [93]), but also coincides with the telencephalo-opto-hypothalamic domain -TOH- identified by Morales et al. [37] and apparently contains *Sim1/Otp* cells migrated from underlying parts of the paraventricular area.

A new concept affects this alar domain where the hypothalamus limits with the telencephalon. The peduncular and terminal subdivisions of the dorsal paraventricular (PPaD/TPaD) domain were recently recognized as a telencephalo-opto-hypothalamic area due to its expression of *Foxg1* (TOH; [37]). This area generates glutamatergic cells co-expressing *Otp*, but not *Nkx2.1* or *Dlx* genes, indicating a lack of subpallial character; these excitatory cells invade partially the BST complex and the medial amygdala [37]. These authors proposed that TOH be considered a novel telencephalic radial domain transitional between subpallium and alar hypothalamus but acknowledged that it essentially corresponds topographically with the dorsal paraventricular hypothalamic subregion of Puelles et al. [17], the domain that builds the HyA (see also [70]). We continue to ascribe the TOH/HyA domain to the alar hypothalamus, given its fundamental genoarchitectonic difference with the *Dlx*-expressing telencephalic subpallium, and considering that ontogenetically the whole telencephalon is a hypothalamic derivative (we are speaking of evaginated and non-evaginated parts of the same alar plate, that of the hp1 and hp2 prosomeres). There is no difficulty in assuming that *Foxg1* expression extends dorsoventrally out of the topologically dorsal telencephalic alar region into the continuous hypothalamic one, coming to overlap there partly the field of paraventricular *Sim1/Otp* signal. This interpretation also keeps the definition of the telencephalon simple, limited to subpallium and pallium. We only must know now that the PPaD also expresses *Foxg1*, being thus partially distinct molecularly from the central (PPaC) and ventral (PPaV) paraventricular subregions devoid of this signal. TOH/PaD cells also lack *Brn2* (*Pou3f2*) expression, present in the central and ventral Pa subdomains [110].

Present data indicate that there are LH9 cells expressing the transcription factors *Fezf2*, *Foxg1*, *Otp*, *Satb2*, *Sim1*, and *Zic5* (*Fezf2* is a regulator of *Otp* expression in zebrafish oxytocinergic hypothalamic neurons; [177]). In addition, LH9 contains selectively *Nos1*–expressing cells. Puelles et al. [17] reported -but did not show- a large *Nos1*-positive perifornical population surrounding the retrocommissural sector of the fornix within their pre-eminential perifornical nucleus.

LH9 selectively contains numerous dispersed *Satb2*-expressing cells intermingled with the medial forebrain bundle (Fig. 9H; see also [178]), laterally to a dense *Satb2*-positive periventricular island (see Fig. 9I). This population lying apparently at the hypothalamo-telencephalic border zone (coinciding with the dorsal part of the hypothalamic paraventricular complex) should accordingly be considered an additional LH subregion representing the end of the lateral hypothalamus next to the subpallial telencephalon (e.g., substantia innominata).

### Differential Connectivity Pattern of the LH Cell Subregions

The LH is clearly genoarchitecturally heterogenous (LH1-LH9); it is of interest to explore how far it is also hodologically and functionally diverse, irrespective that much literature refers to LH as a unitary concept. Reportedly, the LH plays a critical role in coordinating several physiological and behavioral functions, such as arousal, feeding, stress, reward, and motivated behaviors [1, 4, 7–11, 28, 179–181]. The LH has extensive reciprocal connections with multiple brain regions. Ascending LH projections are implicated in cognitive and emotional processing, in contrast with descending projections to the brainstem and spinal cord related to wakefulness, feeding, and various autonomic and neuroendocrine functions (reviewed in [28]). A major problem in the study of LH connectivity using tracer techniques (apart from the structural map of LH subdivisions considered) is the distinction of potential co-labeling of medial forebrain bundle fibers of passage.

A good number of connectivity experiments centered at different dorsoventral LH levels (according to a prosomeric interpretation; note authors using the columnar model will describe them as anteroposterior or rostrocaudal partitions) have shown differential connections that can be related with our proposal of LH genoarchitectonic regionalization (e.g. [25, 75, 160, 182–192]). For instance, Pelosi et al. [191] showed a distinct dorsoventral regionalization (described as rostrocaudal) of the efferent LH projections to different parts of the dorsal periaqueductal gray in the rat. Likewise, Hahn and Swanson [25], comparing afferents and efferents of (alar) juxtaparaventricular and (basal) suprafornical LH subregions, demonstrated that these domains have different connectivity patterns and deduced different behavior roles (defensive versus ingestive). It was also reported that olfactory input reaches specifically the basal LH [193]. Unfortunately, interpretation and systematization of such interesting results within the columnar paradigm are difficult and usually approximative. The columnar authors are limited to crude preoptic, anterior, tuberal, and posterior hypothalamic LH subregions devoid of postulated molecularly distinct progenitor domains. The columnar concept contrarily presupposes longitudinal homogeneity (the essence of Herrick’s columns [194], as also conceived by Kuhlenbeck [195], an already false notion) and thus lacks from its origin a conceptual underpinning for the sort of subdivisions we deal with here (what developmental mechanism would pattern a *longitudinal column* into 9 subdomains?). What the alternative prosomeric model resolves as easily explainable dorsoventral pattern generated by well-established antagonism between floor-induced ventralization and roof-induced dorsalization is not easily imitated within columnar theory because no rostrocaudal patterning mechanism is admitted there and the floorplate is defined differently. The same theoretic antecedents lead to non-recognition of alar/basal subregions within the hypothalamus (the whole hypothalamus is newly postulated as a basal territory [20]). Another difference with our prosomeric approach is that the LH is conventionally thought to pertain to the whole hypothalamus, while the radial glial structure indicates that it essentially relates to our peduncular part of the hypothalamus, derived specifically from prosomere hp1 ([17], their Fig. 12; [26]; present results). We conclude that connectivity data need to be reinterpreted or repeated within the prosomeric paradigm.

## Supplementary Information

Below is the link to the electronic supplementary material.

Supplementary file1 (DOCX 18 KB)

## Data Availability

Most of our analysis was based on in situ hybridization images downloaded from the Allen Developing Mouse Brain Atlas (https://developingmouse.brain-map.org) and Allen Mouse Brain Atlas (https://mouse.brain-map.org/). Other data are available from the corresponding author on reasonable request.

## Abbreviations

a/b: Alar/basal border
A13: Dopaminergic group A13
ABas: Anterobasal area and nucleus
ABasW: Wing (terminal) portion of ABas
ac: Anterior commissure
AH: Anterior hypothalamic area
Ahí: Amygdalo-hippocampal transitional area
Ar: Arcuate nucleus
BST: Bed nucleus of the stria terminalis
BSTL: Lateral part of the BST
BSTM: Medial part of the BST
CLi: Caudal liminar band
Dg: Diagonal area
DM: Dorsomedial hypothalamic nucleus
DMcT: Core of the terminal dorsomedial hypothalamic nucleus
DM-P: Peduncular dorsomedial hypothalamic nucleus
DMs: Shell of the dorsomedial hypothalamic nucleus
DM-T: Terminal dorsomedial hypothalamic nucleus
DPM: Dorsal premamillary hypothalamic nucleus
EPA: Accessory entopeduncular nucleus
EPD: Dorsal entopeduncular nucleus
EPV: Ventral entopeduncular nucleus
f: Fornix tract
fi: Fimbria
GP: Globus pallidus
GPE: Globus pallidus externus
GPI: Globus pallidus internus
Hb: Habenula
HDB: Hypothalamo-diencephalic boundary
Hi: Hippocampus
hp1: Hypothalamo-telencephalic prosomere 1
hp2: Hypothalamo-telencephalic prosomere 2
HyA: Hypothalamo-amygdalar corridor
ic: Internal capsule
IHB: Intrahypothalamic boundary (hp1/hp2)
In: Infundibulum
ivf: Interventricular foramen
LA: Lateroanterior hypothalamic nucleus
LH1: Lateral hypothalamic subregion 1
LH2: Lateral hypothalamic subregion 2
LH3: Lateral hypothalamic subregion 3
LH4: Lateral hypothalamic subregion 4
LH5: Lateral hypothalamic subregion 5
LH6: Lateral hypothalamic subregion 6
LH7: Lateral hypothalamic subregion 7
LH8: Lateral hypothalamic subregion 8
LH9: Lateral hypothalamic subregion 9
LM: Lateral mamillary nucleus
LOT: Nucleus of the lateral olfactory tract
LPa: Lateral paraventricular nucleus
MA: Medial amygdala
ME: Medial eminence
mfb: Medial forebrain bundle
MH: Medial hypothalamus
ml: Medial lemniscus
MMl: Medial mamillary nucleus, lateral part
MMm: Medial mamillary nucleus, medio-medial part
mtg: Mamillotegmental tract
mth: Mamillothalamic tract
NHy: Neurohypophysis
ns: Nigrostriatal tract
och: Optic chiasma
os: Optic recess
ot: Optic tract
p2: Prosomere 2
p2tg: Tegmentum of prosomere 2
p3: Prosomere 3
p3tg: Tegmentum of prosomere 3
PaC: Paraventricular central nucleus
PaD: Paraventricular dorsal nucleus
PaL: Lateral paraventricular nucleus
Pall: Pallium
PaV: Paraventricular ventral nucleus
PBas: Posterobasal area and nucleus.
pe: Peduncle (lateral forebrain bundle)
Pf1: Perifornical subregion 1
Pf2: Perifornical subregion 2
Pf3: Perifornical subregion 3
Pf4: Perifornical subregion 4
Pf5: Perifornical subregion 5
Pf6: Perifornical subregion 6
Pf7: Perifornical subregion 7
Pf8: Perifornical subregion 8
Pf9: Perifornical subregion 9
PHy: Peduncular hypothalamus
POA: Preoptic area
PPa: Peduncular paraventricular domain
PPaC: Central part of PPa
PPaD: Dorsal part of PPa
PPaV: Ventral part of PPa
PRM: Periretromamillary domain
pRt: Prereticular nucleus
PSPa: Peduncular subparaventricular domain
PSTh: Parasubthalamic nucleus
PThE: Prethalamic eminence
Re: Reuniens nucleus
rf: Retroflexus tract
RLi: Rostral liminar band
RM: Retromamillary domain
RML: Lateral retromamillary nucleus
RMM: Medial retromamillary nucleus
RMS: Superficial retromamillary nucleus
RSTh: Retrosubthalamic nucleus
Rt: Reticular complex
RTu: Retrotuberal domain
RTuD: Dorsal part of RTu
RTuI: Intermediate part of RTu
RTuV: Ventral part of RTu
SCh: Suprachiasmatic nucleus
Se: Septum
sm: Stria medullaris
SN: Substantia nigra
SNC: Substantia nigra, compact part
SNR: Substantia nigra, reticular part
SO: Supraoptic nucleus
SPa: Subparaventricular domain
St: Striatum
st: Stria terminalis
STh: Subthalamic nucleus
Th: Thalamus
THy: Terminal hypothalamus
TOH: Telencephalo-opto-hypothalamic area
TSPa: Terminal subparaventricular domain
Tu: Tuberal area
TuL: Lateral tuberal nucleus
TuM: Medial tuberal nucleus
TuSbO: Tuberal suboptic nucleus
TuV: Ventral part of tuberal domain
v: Ventricle
VM: Ventromedial hypothalamic nucleus
VMdm: Ventromedial hypothalamic nucleus, dorsomedial part
VPM: Ventral premamillary hypothalamic nucleus
VTA: Ventral tegmental area
ZI: Zona incerta complex
zl: Zona limitans
