# Supplementary material for: Dorsoventral Arrangement of Lateral Hypothalamus Populations in the Mouse Hypothalamus: a Prosomeric Genoarchitectonic Analysis
Source: Mol Neurobiol. 2022 Nov 11;60(2):687–731. doi: 10.1007/s12035-022-03043-7 (PMC9849321; doi:10.1007/s12035-022-03043-7)
Supplement: Supplementary file 1 — Supplementary file1 (DOCX 18 KB) [file 12035_2022_3043_MOESM1_ESM.docx]

**Supplementary Table 1 List of genes illustrated in given figures, which correspond as indicated to experiment images downloaded from the Allen Developing Mouse Brain Atlas (developingmouse.brain-map.org).**

| **Gene marker** | **Image number** | **Experiment** |
| --- | --- | --- |
| **Figure 3** |  |  |
| ***Tac1*** | **29** | **P56 1038** |
|  | **30** | **P56 1038** |
|  | **13** | **P56 1039** |
| ***Chrm3*** | **25** | **P56 79912556** |
|  | **28** | **P56 79912557** |
|  | **14** | **P56 77464862** |
| ***Pitx2*** | **10** | **P56 100145380** |
|  | **32** | **P56 79556607** |
| ***Nnat*** | **27** | **P56 77887874** |
| ***Cbln2*** | **66** | **P56 70231306** |
| ***Erbb4*** | **32** | **P56 72472797** |
|  | **31** | **P56 72472798** |
| **Figure 4** |  |  |
| ***Otp*** | **9** | **E11.5 100085515** |
| ***Sim1*** | **7** | **E11.5 100077795** |
| ***Irs4*** | **11** | **E13.5 100046578** |
| ***Penk*** | **13** | **E18.5 100055886** |
|  | **18** | **P56 69116554** |
|  | **60** | **P56 74881286** |
| ***Ebf3*** | **9** | **E18.5 100077734** |
|  | **14** | **P56 73712894** |
| ***Mdga1*** | **7** | **P4 100057320** |
| ***Peg10*** | **10** | **E18.5 100071652** |
|  | **12** | **P56 73497633** |
|  | **80** | **P56 74357549** |
| **Figure 5** |  |  |
| ***Hdc*** | **13** | **P56 68667587** |
|  | **14** | **P56 68667588** |
|  | **58** | **P56 71016663** |
|  | **59** | **P56 71016664** |
| ***Prph*** | **59** | **P56 70232000** |
| ***Wif1*** | **56** | **P56 74511885** |
| **Figure 6** |  |  |
| ***Nts*** | **14** | **P56 71234690** |
|  | **49** | **P56 73788032** |
| ***Tac2*** | **51** | **P56 72339556** |
| ***Cbln2*** | **59** | **P56 70231306** |
| ***Cartpt*** | **13** | **P56 79677369** |
|  | **26** | **P56 72077479** |
| ***Peg10*** | **60** | **P56 74357549** |
| ***Ecel1*** | **53** | **P56 70231305** |
| ***Nek7*** | **49** | **P56 72339542** |
| ***Pdyn*** | **55** | **P56 72339542** |
| ***Penk*** | **17** | **P56 100082378** |
|  | **57** | **P56 74881286** |
| **Figure 7** |  |  |
| ***Hcrt*** | **11** | **P28 100045534** |
|  | **13** | **P28 100045535** |
| ***Irs4*** | **29** | **P1 100091988** |
| ***Nnat*** | **23** | **P56 77887874** |
| ***Gpx3*** | **27** | **P56 75080750** |
| ***Pmch*** | **9** | **P4 100084463** |
|  | **15** | **P28 100083563** |
|  | **23** | **P56 55** |
| ***Gal*** | **52** | **P56 70231997** |
| ***Pdyn*** | **14** | **P56 69782969** |
|  | **52** | **P56 71717084** |
| ***Penk*** | **55** | **P56 74881286** |
| **Figure 8** |  |  |
| ***Pnoc*** | **10** | **E18.5 100071453** |
| ***Sst*** | **23** | **P56 1001** |
| ***Slc17a6*** | **47** | **P56 73818754** |
| ***Meis2*** | **14** | **P56 1232** |
|  | **22** | **P56 1231** |
| ***Cacna2d1*** | **24** | **P56 72119649** |
| ***Slc17a6*** | **44** | **P56 73818754** |
| ***Lhx5*** | **19** | **E18.5 100059231** |
| ***Tbr1*** | **23** | **P56 79591351** |
| **Figure 9** |  |  |
| ***Nkx2.1*** | **11** | **E13.5 100093267** |
| ***Otp*** | **10** | **E13.5 100075818** |
| ***Foxg1*** | **13** | **E13.5 100055651** |
| ***Sim1*** | **9** | **E13.5 100029661** |
| ***Satb2*** | **7** | **E13.5 100036914** |
| ***Foxg1*** | **15** | **E18.5 100072314** |
| ***Sim1*** | **13** | **E18.5 100088173** |
| ***Satb2*** | **7** | **E18.5 100055962** |
|  | **18** | **E18.5 100092410** |
| **Figure 10** |  |  |
| ***Fezf2*** | **11** | **P4 100032251** |
| ***Nos1*** | **43** | **P56 75147762** |
| ***Zic5*** | **10** | **E18.5 100071380** |
|  | **11** | **E18.5 100071381** |
